# Interventions for patent ductus arteriosus (PDA) in preterm infants: an overview of Cochrane Systematic Reviews

**DOI:** 10.1002/14651858.CD013588.pub2

**Published:** 2023-04-11

**Authors:** Souvik Mitra, Willem P Boode, Dany E Weisz, Prakeshkumar S Shah

**Affiliations:** Departments of Pediatrics, Community Health & EpidemiologyDalhousie University & IWK Health CentreHalifaxCanada; Department of Perinatology, Division of NeonatologyRadboud UMC Amalia Children’s HospitalNijmegenNetherlands; Department of Newborn and Developmental PaediatricsSunnybrook Health Sciences CentreTorontoCanada; Department of Paediatrics and Institute of Health Policy, Management and EvaluationUniversity of Toronto Mount Sinai HospitalTorontoCanada

**Keywords:** Humans, Infant, Newborn, Acetaminophen, Acetaminophen/therapeutic use, Cyclooxygenase Inhibitors, Cyclooxygenase Inhibitors/adverse effects, Ductus Arteriosus, Patent, Ductus Arteriosus, Patent/drug therapy, Ibuprofen, Ibuprofen/adverse effects, Indomethacin, Indomethacin/therapeutic use, Infant, Premature, Prostaglandin Antagonists, Prostaglandin Antagonists/therapeutic use, Systematic Reviews as Topic

## Abstract

**Background:**

Patent ductus arteriosus (PDA) is associated with significant morbidity and mortality in preterm infants. Several non‐pharmacological, pharmacological, and surgical approaches have been explored to prevent or treat a PDA.

**Objectives:**

To summarise Cochrane Neonatal evidence on interventions (pharmacological or surgical) for the prevention of PDA and related complications, and interventions for the management of asymptomatic and symptomatic PDA in preterm infants.

**Methods:**

We searched the Cochrane Database of Systematic Reviews on 20 October 2022 for ongoing and published Cochrane Reviews on the prevention and treatment of PDA in preterm (< 37 weeks' gestation) or low birthweight (< 2500 g) infants. We included all published Cochrane Reviews assessing the following categories of interventions: pharmacological therapy using prostaglandin inhibitor drugs (indomethacin, ibuprofen, and acetaminophen), adjunctive pharmacological interventions, invasive PDA closure procedures, and non‐pharmacological interventions. Two overview authors independently checked the eligibility of the reviews retrieved by the search, and extracted data from the included reviews using a predefined data extraction form. Any disagreements were resolved by discussion with a third overview author. Two overview authors independently assessed the methodological quality of the included reviews using the AMSTAR 2 (A MeaSurement Tool to Assess systematic Reviews) tool. We reported the GRADE certainty of evidence as assessed by the respective review authors using summary of findings tables.

**Main results:**

We included 16 Cochrane Reviews, corresponding to 138 randomised clinical trials (RCT) and 11,856 preterm infants, on the prevention and treatment of PDA in preterm infants. One of the 16 reviews had no included studies, and therefore, did not contribute to the results. Six reviews reported on prophylactic interventions for the prevention of PDA and included pharmacological prophylaxis with prostaglandin inhibitor drugs, prophylactic surgical PDA ligation, and non‐pharmacologic interventions (chest shielding during phototherapy and restriction of fluid intake); one review reported on the use of indomethacin for the management of asymptomatic PDA; nine reviews reported on interventions for the management of symptomatic PDA, and included pharmacotherapy with prostaglandin inhibitor drugs in various routes and dosages, surgical PDA ligation, and adjunct therapies (use of furosemide and dopamine in conjunction with indomethacin). The quality of reviews varied. Two reviews were assessed to be high quality, seven reviews were of moderate quality, five of low quality, while two reviews were deemed to be of critically low quality.

For prevention of PDA, prophylactic indomethacin reduces severe intraventricular haemorrhage (IVH; relative risk (RR) 0.66, 95% confidence interval (CI) 0.53 to 0.82; 14 RCTs, 2588 infants), and the need for invasive PDA closure (RR 0.51, 95% CI 0.37 to 0.71; 8 RCTs, 1791 infants), but it does not appear to affect the composite outcome of death or moderate/severe neurodevelopmental disability (RR 1.02, 95% CI 0.90 to 1.15; 3 RCTs, 1491 infants). Prophylactic ibuprofen probably marginally reduces severe IVH (RR 0.67, 95% CI 0.45 to 1.00; 7 RCTs, 925 infants; moderate‐certainty evidence), and the need for invasive PDA closure (RR 0.46, 95% CI 0.22 to 0.96; 7 RCTs, 925 infants; moderate‐certainty evidence). The evidence is very uncertain on the effect of prophylactic acetaminophen on severe IVH (RR 1.09, 95% CI 0.07 to 16.39; 1 RCT, 48 infants). Necrotising enterocolitis (NEC) was lower with both prophylactic surgical ligation (RR 0.25, 95% CI 0.08 to 0.83; 1 RCT, 84 infants), and fluid restriction (RR 0.43, 95% CI 0.21 to 0.87; 4 RCTs, 526 infants).

For treatment of asymptomatic PDA, indomethacin appears to reduce the development of symptomatic PDA post‐treatment (RR 0.36, 95% CI 0.19 to 0.68; 3 RCTs, 97 infants; quality of source review: critically low).

For treatment of symptomatic PDA, all available prostaglandin inhibitor drugs appear to be more effective in closing a PDA than placebo or no treatment (indomethacin: RR 0.30, 95% CI 0.23 to 0.38; 10 RCTs, 654 infants; high‐certainty evidence; ibuprofen: RR 0.62, 95% CI 0.44 to 0.86; 2 RCTs, 206 infants; moderate‐certainty evidence; early administration of acetaminophen: RR 0.35, 95% CI 0.23 to 0.53; 2 RCTs, 127 infants; low‐certainty evidence). Oral ibuprofen appears to be more effective in PDA closure than intravenous (IV) ibuprofen (RR 0.38, 95% CI 0.26 to 0.56; 5 RCTs, 406 infants; moderate‐certainty evidence). High‐dose ibuprofen appears to be more effective in PDA closure than standard‐dose ibuprofen (RR 0.37, 95% CI 0.22 to 0.61; 3 RCTs, 190 infants; moderate‐certainty evidence). With respect to adverse outcomes, compared to indomethacin administration, NEC appears to be lower with ibuprofen (any route; RR 0.68, 95% CI 0.49 to 0.94; 18 RCTs, 1292 infants; moderate‐certainty evidence), oral ibuprofen (RR 0.41, 95% CI 0.23 to 0.73; 7 RCTs, 249 infants; low‐certainty evidence), and with acetaminophen (RR 0.42, 95% CI 0.19 to 0.96; 4 RCTs, 384 infants; low‐certainty evidence). However, NEC appears to be increased with a prolonged course of indomethacin versus a shorter course (RR 1.87, 95% CI 1.07 to 3.27; 4 RCTs, 310 infants).

**Authors' conclusions:**

This overview summarised the evidence from 16 Cochrane Reviews of RCTs regarding the effects of interventions for the prevention and treatment of PDA in preterm infants.

Prophylactic indomethacin reduces severe IVH, but does not appear to affect the composite outcome of death or moderate/severe neurodevelopmental disability. Prophylactic ibuprofen probably marginally reduces severe IVH (moderate‐certainty evidence), while the evidence is very uncertain on the effect of prophylactic acetaminophen on severe IVH. All available prostaglandin inhibitor drugs appear to be effective in symptomatic PDA closure compared to no treatment (high‐certainty evidence for indomethacin; moderate‐certainty evidence for ibuprofen; low‐certainty evidence for early administration of acetaminophen). Oral ibuprofen appears to be more effective in PDA closure than IV ibuprofen (moderate‐certainty evidence). High dose ibuprofen appears to be more effective in PDA closure than standard‐dose ibuprofen (moderate‐certainty evidence).

There are currently two ongoing reviews, one on fluid restriction for symptomatic PDA, and the other on invasive management of PDA in preterm infants.

## Plain language summary

**Treatments to manage patent ductus arteriosus in premature babies**

**Review question**

What treatments are effective and safe in preventing or treating a common heart condition, called patent ductus arteriosus (PDA) in premature babies?

**Background**

PDA is a common complication among premature and low birthweight babies. PDA is an open blood vessel channel between the lungs and the heart, which usually closes shortly after birth. In premature and low birthweight babies, the PDA may remain open, and may contribute to life‐threatening complications. We wanted to see what treatments can safely and effectively prevent or treat a PDA and its related problems.

**Study characteristics**

We included 16 Cochrane Reviews. Out of them, six reviews provided evidence on preventing a PDA with drugs, surgery, or other means that do not involve drugs or surgery. One review provided evidence of treating a PDA before the babies experience symptoms, while the rest of the reviews provided evidence on treating babies who are experiencing symptoms from their PDA, with either drugs or surgery.

**Key Results**

This overview found that both indomethacin and ibuprofen may reduce severe brain bleeding and the need for PDA surgery, when given to premature babies before they experienced symptoms from a PDA. When babies are experiencing symptoms from a PDA, all available drug therapies, that is, indomethacin, ibuprofen, and acetaminophen (specifically when given early) are effective in closing a PDA. If using ibuprofen therapy, giving the medication by mouth appears to be better than giving it by intravenous route; and higher doses of ibuprofen appears to be more effective in closing a PDA than standard doses.

**Certainty of the evidence**

According to GRADE (a method to score the certainty of the trials supporting each outcome), the certainty of the evidence varied from very low to high. According to the AMSTAR 2 criteria (a method to rate the quality of reviews), the quality of the included Cochrane Reviews also varied from high to critically low, but were mostly between moderate and low.

**How up to date is the search evidence**

The search is up to date as of 20 October 2022.

## Background

### Description of the condition

The ductus arteriosus is a blood vessel that connects the main pulmonary artery to the proximal descending aorta. It plays an important role in maintaining foetal circulation by allowing a significant proportion of right ventricular output to bypass the pulmonary circulation (Gournay 2011). Following birth, with establishment of respiration and separation of low‐resistance placenta, closure of the ductus arteriosus begins. This closure is triggered by physiological mechanisms, such as increased oxygen tension and decreased circulating prostaglandin (PGE₂) and prostacyclin (PGI₂ (Hundscheid 2019)). Functional closure of the ductus arteriosus occurs over the next 24 to 72 hours in term infants (Benitz 2016). In preterm infants, closure is often delayed, leading to the ductus arteriosus remaining patent beyond the first few days of life. In healthy preterm neonates, born at > 30 weeks' gestation, the patent ductus arteriosus (PDA) closes by day four in 90%, and by day seven in 98% of infants (Clyman 2012). In extremely preterm infants, born at < 24 weeks' gestation, spontaneous PDA closure rates are only about 8% by day four and 13% by day seven (Clyman 2012).

Therefore, a PDA often persists beyond the first few days of life in a preterm neonate, but may remain asymptomatic, without inducing any adverse haemodynamic consequences in the neonate. However, with progressive decline in pulmonary vascular resistance, blood flow from the aorta into the pulmonary arteries is increased through the PDA. Consequently, the proportion of aortic blood flow that is diverted into the pulmonary circulation is correspondingly increased (Benitz 2016). This 'ductal steal' may result in excessive blood flow through the lungs, predisposing the development of pulmonary congestion, pulmonary oedema, worsening respiratory failure, and eventually, chronic lung disease (CLD (Benitz 2016)). At the same time, diversion of blood flow away from the systemic circulation may lead to systemic hypoperfusion, resulting in compromised perfusion to the bowel, kidney, and brain. When a PDA is associated with clinical and echocardiographic signs of pulmonary hyperperfusion and systemic hypoperfusion, this is labelled a symptomatic PDA, or a haemodynamically significant PDA. A persistently symptomatic PDA may be associated with numerous adverse outcomes, including higher rates of death (Dice 2007), bronchopulmonary dysplasia (BPD (Brown 1979)), necrotising enterocolitis (NEC (Dollberg 2005)), impaired renal function (Benitz 2016), intraventricular haemorrhage (IVH (Ballabh 2010)), periventricular leukomalacia (PVL (Chung 2005)), and cerebral palsy (Drougia 2007). However, the causal link between these associations has not been demonstrated (Benitz 2010).

### Description of the interventions

A PDA can be closed through medical or surgical interventions. Pharmacotherapeutic agents include non‐steroidal anti‐inflammatory drugs (NSAIDs), such as ibuprofen or indomethacin, and acetaminophen, which is a derivative of acetanilide with anti‐inflammatory properties. Surgical interventions include surgical ligation and transcatheter occlusion. In this overview, we will focus on both pharmacotherapeutic and surgical interventions for prevention and treatment of preterm infants with PDA.

NSAIDs act by inhibiting the cyclo‐oxygenase (COX) enzyme, thereby leading to down regulation of PGE₂, a potent relaxant of the PDA (Mahony 1982). However, use of indomethacin in preterm infants has been associated with transient or permanent derangement of renal function (Seyberth 1983), NEC (Coombs 1990), gastrointestinal haemorrhage or perforation (Wolf 1989), alteration of platelet function (Friedman 1976), and impairment of cerebral blood flow/cerebral blood flow velocity (Ohlsson 1993). Therefore, variations in indomethacin therapy have been attempted to mitigate the said adverse effects while maximising therapeutic benefit. These include using continuous infusion of indomethacin rather than intermittent bolus doses, which may reduce its adverse effects on cerebral oxygenation (Hammerman 1995), and use of a prolonged course of indomethacin, which may provide increased therapeutic benefit compared to a short course of indomethacin (Rennie 1991).

Ibuprofen exerts its action through inhibition of the COX enzyme, but appears to be associated with lower risk of NEC and transient renal insufficiency, compared to indomethacin (Ohlsson 2020b). However, variation in dosage and route of administration of ibuprofen may impact medication effectiveness. It has been demonstrated that to achieve optimal concentrations of ibuprofen for successful PDA closure, irrespective of gestational age, progressively higher doses are required with increasing postnatal age (Hirt 2008). Similar pharmacokinetic studies have shown that peak serum concentrations following oral ibuprofen therapy are significantly higher than previously demonstrated intravenous levels, suggesting a potential for greater responsiveness to oral ibuprofen compared to the intravenous formulation (Barzilay 2012).

Acetaminophen is postulated to exert its action through inhibition of the peroxidase enzyme, thereby leading to down regulation of PGE₂ production (Gillam‐Krakauer 2018; Grèen 1989). No short‐term adverse effects have been noted with acetaminophen. However, data on the safety and long‐term neurodevelopmental effects of acetaminophen in preterm infants are limited (Ohlsson 2020; Van den Anker 2018).

With increasing emphasis on conservative management, surgical PDA ligation is primarily reserved for infants with persistent symptomatic PDA following the failure of medical management. Surgical PDA ligation is associated with reduced mortality, but surviving infants were found to be at increased risk of neurodevelopmental impairment, which could be due to lack of studies addressing survival bias and confounding by indication (Weisz 2014).

### How the intervention might work

Prevention and treatment of a PDA via the most effective modality may help to avoid clinical complications associated with persistent PDA, such as mortality, CLD, NEC, and renal failure. Prevention of PDA includes prophylactic medical or surgical closure of the PDA within the first 24 hours after birth, before the development of clinical symptoms (Benitz 2016). Although one of the earliest randomised trials on PDA management used prophylactic surgical PDA ligation, this is no longer a preferred modality in the current clinical context, given its associated risks and the availability of pharmacotherapeutic options (Cassady 1989). Hence, prophylactic management of the PDA essentially involves the use of pharmacotherapeutic agents within the first 24 hours of life, without knowledge of PDA status. Prophylactic use of intravenous indomethacin has been shown to reduce the incidence of symptomatic PDA, surgical PDA ligation, and the incidence of severe intraventricular haemorrhage, but has no effect on mortality, nor on a composite of death or severe neurodevelopmental disability, compared to placebo or no treatment (Fowlie 2010). Prophylactic ibuprofen, compared to placebo or no intervention, has also been shown to reduce the need for rescue treatment with COX inhibitors, and for surgical PDA closure (Ohlsson 2020a). However, both prophylactic indomethacin and prophylactic ibuprofen have been shown to be associated with increased risk of oliguria (Fowlie 2010; Ohlsson 2020a). Prophylactic ibuprofen is further associated with increased risk of gastrointestinal haemorrhage (Ohlsson 2020a). Therefore, interest in expectant management of the PDA in preterm infants is growing, and the safety of this approach remains to be established through large randomised controlled trials (Hundscheid 2018).

On the other hand, treatment of PDA entails pharmacotherapeutic or surgical closure of a PDA, the diagnosis of which was based on characteristic clinical symptoms, echocardiographic findings, or both. NSAIDs and acetaminophen have been shown to be more effective in closing a symptomatic PDA compared to placebo (Mitra 2018). Ibuprofen appears to be as effective as indomethacin in closing a symptomatic PDA, while reducing the risk of NEC and transient renal insufficiency (Ohlsson 2020b). Moderate‐quality evidence shows that acetaminophen is as effective as ibuprofen, and low‐quality evidence suggests that acetaminophen is as effective as indomethacin in closing a symptomatic PDA (Ohlsson 2020). Data are inconclusive regarding the efficacy and safety of surgery as the initial modality of treatment for a symptomatic PDA in a preterm infant compared to pharmacotherapeutic management (Malviya 2013).

### Why it is important to do this overview

Management of the PDA is one of the most controversial topics in neonatal medicine. Prophylactic treatment with indomethacin reduces the need for surgical PDA ligation and severe periventricular and intraventricular haemorrhage, but does not improve the rate of survival without neurosensory impairment at 18 months (Schmidt 2001). There are also concerns about the increased incidence of spontaneous gastrointestinal perforation with prophylactic indomethacin (Stavel 2017). On the other hand, prophylactic use of ibuprofen for PDA in preterm infants has been associated with severe hypoxaemia, pulmonary hypertension, and gastrointestinal haemorrhage (Gournay 2002). Therefore, debate on whether NSAIDs should be routinely used to prevent PDA in preterm infants is ongoing. To date, four Cochrane Neonatal Reviews have examined prophylactic medical or surgical management of PDA in preterm infants (Fowlie 2010; Mosalli 2008; Ohlsson 2020; Ohlsson 2020a). There is also debate about whether treatment of an asymptomatic PDA before the development of a significant left‐to‐right shunt improves clinical outcomes. One Cochrane Neonatal Review explored the question of treatment for asymptomatic PDA (Cooke 2003). Similarly, when it comes to treatment for a symptomatic PDA, the availability of multiple management strategies contributes to the dilemma among clinicians. In a recent systematic review and network meta‐analysis, 15 different pharmacotherapeutic options were identified that have been explored in randomised clinical trials for the management of symptomatic PDA (Mitra 2018). The Cochrane Reviews published so far on this topic tackled the problem from a narrow perspective, as all of them compared only two out of several available interventions against each other. Some of these reviews lacked an assessment of the quality of the evidence, using GRADE, and reviews showed variation in the definitions of symptomatic PDA, interventions, and outcomes described. Therefore, an overview of available Cochrane Neonatal Reviews was justified, as it helped to summarise the evidence generated so far on the management strategies available for PDA in preterm infants with respect to the most important outcomes, including the quality of the evidence, and also highlighted important gaps in knowledge that may guide future research on PDA management.

### Is an overview the right approach?

We followed the Editorial Decision Tree proposed by the Cochrane Comparing Multiple Intervention Methods Group to establish whether our review would better fit an overview format or an intervention review format. We decided that for the purposes of this review, to (Shepherd 2018):

1. review only systematic reviews published in the Cochrane Database of Systematic Reviews, instead of individual trials;
2. not compare multiple interventions with the intention of drawing inferences about the comparative effectiveness of these interventions, as we cannot draw conclusions on the transitivity assumption from systematic reviews only; and
3. present a map of evidence from systematic reviews, but with no attempt to rank the interventions.

On the basis of these points, the Editorial Decision Tree recommended that an overview was the appropriate format for this review.

## Objectives

To summarise Cochrane Neonatal evidence on:

1. interventions (pharmacological or surgical) for prevention of patent ductus arteriosus and related complications in preterm infants; and
2. interventions (pharmacological or surgical) for management of patent ductus arteriosus in preterm infants, including:
  - interventions for management of asymptomatic patent ductus arteriosus in preterm infants; and
  - interventions for management of symptomatic (haemodynamically significant) patent ductus arteriosus in preterm infants.

## Methods

### Criteria for considering reviews for inclusion

#### Types of studies

In this overview of systematic reviews, we included only published Cochrane Systematic Reviews on the management of patent ductus arteriosus (PDA) in a preterm infant.

#### Types of participants

##### For objective 1 (prevention of PDA)

Preterm (gestational age < 37 weeks at birth) or low‐birth‐weight infants (< 2500 g).

##### For objective 2 (management of PDA)

Preterm (gestational age < 37 weeks at birth) or low‐birth‐weight infants (< 2500 g) with PDA diagnosed clinically, or via echocardiography, or both, in the neonatal period (< 28 days).

We defined an asymptomatic PDA clinically by the presence of a precordial murmur or echocardiographically (presence of left‐to‐right PDA shunt), without clinical signs of a moderate‐ to high‐volume left‐to‐right shunt (hyperdynamic precordial impulse, tachycardia, bounding pulses, widened pulse pressure, worsening respiratory status, hypotension, or cardiac failure).

We defined a symptomatic PDA clinically by the presence of a precordial murmur, along with one or more of the following signs: hyperdynamic precordial impulse, tachycardia, bounding pulses, widened pulse pressure, worsening respiratory status, hypotension, or cardiac failure. We defined a symptomatic PDA echocardiographically by a moderate to large transductal diameter, with or without evidence of pulmonary over‐circulation, with or without evidence of systemic hypoperfusion. We also defined a symptomatic PDA as a combination of left‐to‐right PDA shunt on echocardiography, along with clinical signs of a high‐volume left‐to‐right shunt (hyperdynamic precordial impulse, tachycardia, bounding pulses, widened pulse pressure, worsening respiratory status, hypotension, or cardiac failure).

#### Types of interventions

In this overview, we specifically included reviews of therapies primarily intended to prevent or manage a PDA.

##### For objective 1 (prevention of PDA)

Interventions included prophylactic (not guided by knowledge of PDA status) pharmacological or surgical treatment of PDA within 24 hours of birth. Pharmacological treatments included indomethacin, ibuprofen, and acetaminophen compared against each other, or placebo, or no treatment. There were no restrictions on dose, route, or duration of treatment. Surgical interventions included surgical or transcatheter PDA closure compared against medical treatment, or placebo, or no treatment.

##### For objective 2 (management of PDA)

Interventions included pharmacological and surgical treatments for an asymptomatic or a symptomatic PDA. Pharmacological treatments included indomethacin, ibuprofen, and acetaminophen compared against each other, or placebo, or no treatment. There were no restrictions on dose, route, or duration of treatment. Surgical interventions included surgical or transcatheter PDA closure compared against medical treatment, or placebo, or no treatment.

#### Types of outcome measures

##### For objective 1 (prevention of PDA)

###### Primary outcomes

1. Severe intraventricular haemorrhage (IVH; grade III/IV (Papile 1978))
2. Death or moderate/severe neurodevelopmental disability (assessed by a standardised and validated assessment tool, a child developmental specialist, or both) at any age reported (outcome data grouped at 12, 18, and 24 months, if available). Individual components of a neurodevelopmental outcome, defined in individual reviews, were reported if data were available.

###### Secondary outcomes

####### PDA‐related outcomes

1. Symptomatic PDA confirmed on echocardiogram
2. Proportion of infants receiving open‐label medical treatment (cyclo‐oxygenase inhibitor or paracetamol/acetaminophen dosing, or both)
3. Proportion of infants requiring surgical ligation or transcatheter occlusion

####### Other outcomes

1. Chronic lung disease (CLD), defined as oxygen requirement at 36 weeks’ postmenstrual age (Ehrenkranz 2005)
2. Intraventricular haemorrhage (IVH; grade I to IV (Papile 1978))
3. Pulmonary haemorrhage, defined as blood‐stained respiratory secretions with a significant change in respiratory requirements and chest X‐ray (CXR) changes in the presence of echocardiographic evidence of significant left‐to‐right ductal shunting (Kluckow 2014)
4. Retinopathy of prematurity (ROP), defined according to the international classification of ROP (ICCROP 2005)
5. Duration of hospitalisation, defined as total length of hospitalisation from birth to discharge home or mortality, in days
6. Moderate/severe neurodevelopmental disability, assessed by a standardised and validated assessment tool, a child developmental specialist, or both, at any age reported (outcome data grouped at 12, 18, and 24 months, if available). Individual components of a neurodevelopmental outcome were reported if data were available.
7. All‐cause mortality any time before neonatal intensive care unit (NICU) discharge

####### Safety outcomes

1. Necrotising enterocolitis (NEC; stage 2 or greater (Bell 1978))
2. Gastrointestinal perforation, defined by the presence of free air in peritoneal cavity on an abdominal X‐ray (Ohlsson 2020a)
3. Gastrointestinal bleeding within seven days of the first dose of pharmacotherapy
4. Oliguria, defined as less than 1 mL/kg/hour
5. Serum/plasma levels of creatinine (μmol/L) after treatment
6. Increase in serum/plasma levels of creatinine (μmol/L) after treatment
7. Serum/plasma levels of bilirubin (μmol/L) after treatment
8. Increase in serum/plasma levels of bilirubin (μmol/L) after treatment

##### For objective 2 (management of PDA)

###### Primary outcomes

1. Failure of PDA closure after completion of allocated treatment, defined as persistence of symptomatic PDA confirmed clinically, or by echocardiography, or both
2. Death or moderate/severe neurodevelopmental disability, assessed by a standardised and validated assessment tool, a child developmental specialist, or both, at any age reported (outcome data grouped at 12, 18, and 24 months, if available). Individual components of a neurodevelopmental outcome, as defined in individual reviews, will be reported if data are available.

###### Secondary outcomes

####### PDA‐related outcomes

1. Proportion of infants receiving open‐label medical treatment (repeated COX inhibitor or paracetamol/acetaminophen dosing, or both).
2. Proportion of infants requiring surgical ligation or transcatheter occlusion.
3. Proportion of infants receiving open‐label medical or surgical treatment in the placebo/no treatment group.

####### Other outcomes

1. CLD, defined as oxygen requirement at 36 weeks’ postmenstrual age (Ehrenkranz 2005)
2. Pulmonary haemorrhage, defined as blood‐stained respiratory secretions with a significant change in respiratory requirements and chest X‐ray (CXR) changes in the presence of echocardiographic evidence of significant left‐to‐right ductal shunting (Kluckow 2014)
3. Severe intraventricular haemorrhage (IVH; grade III/IV; for studies of asymptomatic treatment (Papile 1978))
4. Retinopathy of prematurity (ROP; according to the international classification of ROP (ICCROP 2005))
5. Duration of hospitalisation, defined as total length of hospitalisation from birth to discharge home or mortality, in days
6. Moderate/severe neurodevelopmental disability, assessed by a standardised and validated assessment tool, a child developmental specialist, or both, at any age reported (outcome data grouped at 12, 18, and 24 months, if available). Individual components of a neurodevelopmental outcome will be reported if data are available.
7. All‐cause mortality any time before NICU discharge

####### Safety outcomes

1. Necrotising enterocolitis (NEC; stage 2 or greater (Bell 1978))
2. Gastrointestinal perforation, defined by the presence of free air in peritoneal cavity on an abdominal X‐ray (Ohlsson 2020a)
3. Gastrointestinal bleeding within seven days of the first dose of pharmacotherapy
4. Oliguria, defined as less than 1 mL/kg/hour
5. Serum/plasma levels of creatinine (μmol/L) after treatment
6. Increase in serum/plasma levels of creatinine (μmol/L) after treatment
7. Serum/plasma levels of bilirubin (μmol/L) after treatment
8. Increase in serum/plasma levels of bilirubin (μmol/L) after treatment

### Search methods for identification of reviews

We searched the Cochrane Database of Systematic Reviews, using the term ‘patent ductus arteriosus’, on 20 October 2022. We used the search term to search ‘all text’, not limited to ‘title, abstract, or keywords’. We did not apply any language or date restrictions. We did not search any other databases.

### Data collection and analysis

We used the standard methods of Cochrane Neonatal.

#### Selection of reviews

Two overview authors (SM and DW) independently assessed for inclusion, all potential systematic reviews identified by the search. Disagreements were resolved through discussion, or if required, a third member of the overview team was consulted (PS).

#### Data extraction and management

Two overview authors (SM and DW) independently extracted data from the reviews, using a standardised form developed in Microsoft Excel. Discrepancies were resolved through discussion, or if needed, through consultation with a third overview author (PS). In the event information regarding review outcomes was unclear or missing, individual studies were accessed for further details.

We extracted data on the following.

1. *Review characteristics.*
  - Review title and authors
  - Date that the review was last assessed as up‐to‐date
  - Number of included trials and numbers of participants in the trials and their characteristics
  - Risk of bias of the included trials, as reported by the review authors; see Quality of studies included within reviews, under Assessment of methodological quality of included reviews
  - Interventions and comparisons relevant to this overview
  - All prespecified outcomes relevant to this overview (their definitions, and whether they were primary or secondary outcomes in the included reviews)
  - Any other characteristics required to assess and report on review quality; see Quality of included reviews, under Assessment of methodological quality of included reviews
2. *Statistical summaries.*
  - Summary intervention effects, including pooled effects (e.g. risk ratios (RRs), odds ratios (ORs), mean differences (MDs), as reported in the individual reviews), 95% confidence intervals (CIs), numbers of studies and participants contributing data to each pooled effect, from comparisons, and for outcomes relevant to this overview, including relevant subgroup analyses
  - Information required to assess and report on the quality of evidence for the intervention effects extracted; see Quality of evidence in included reviews, under Assessment of methodological quality of included reviews

#### Assessment of methodological quality of included reviews

We assessed the methodological quality of each systematic review using the updated AMSTAR 2 (A Measurement Tool to Assess Reviews) instrument (Shea 2017). AMSTAR 2 evaluates the methods used in a review against 16 distinct criteria and assesses the degree to which review methods are unbiased. These criteria are as follows.

1. Did the research questions and inclusion criteria for the review include the components of PICO (Participants, Intervention, Comparison, Outcomes)?
2. Did the report of the review contain an explicit statement that the review methods were established prior to the conduct of the review, and did the report justify any significant deviations from the protocol?
3. Did the review authors explain their selection of the study designs for inclusion in the review?
4. Did the review authors use a comprehensive literature search strategy?
5. Did the review authors perform study selection in duplicate?
6. Did the review authors perform data extraction in duplicate?
7. Did the review authors provide a list of excluded studies and justify the exclusions?
8. Did the review authors describe the included studies in adequate detail?
9. Did the review authors use a satisfactory technique for assessing the risk of bias (RoB) in individual studies that were included in the review?
10. Did the review authors report on the sources of funding for the studies included in the review?
11. If meta‐analysis was performed, did the review authors use appropriate methods for statistical combination of results?
12. If meta‐analysis was performed, did the review authors assess the potential impact of RoB in individual studies on the results of the meta‐analysis or other evidence synthesis?
13. Did the review authors account for RoB in individual studies when interpreting/discussing the results of the review?
14. Did the review authors provide a satisfactory explanation for, and discussion of, any heterogeneity observed in the results of the review?
15. If they performed quantitative synthesis, did the review authors carry out an adequate investigation of publication bias (small‐study bias) and discuss its likely impact on the results of the review?
16. Did the review authors report any potential sources of conflict of interest, including any funding they received for conducting the review?

Two overview authors (SM and PS) independently assessed the quality of the included reviews using the online AMSTAR 2 tool (Shea 2017). A third overview author (WdB) verified the assessment. We resolved differences through discussion.

##### Quality of included studies within reviews

We did not reassess the risk of bias of included studies within reviews. Instead, we reported study quality according to the review authors' assessment. When individual studies were included in two or more Cochrane Reviews, we reported any variation in the review authors' assessments of study quality.

##### Certainty of evidence in included reviews

We used the GRADE approach, as outlined in the *GRADE Handbook* to assess the certainty of evidence for the following (clinically relevant) outcomes (Schünemann 2013).

###### Prevention in at‐risk infants

1. Death or moderate/severe neurodevelopmental disability
2. Symptomatic PDA confirmed on echocardiogram
3. Proportion of infants requiring surgical ligation or transcatheter occlusion
4. All‐cause mortality any time prior to NICU discharge
5. CLD, defined as oxygen requirement at 36 weeks’ postmenstrual age
6. Necrotising enterocolitis (NEC; stage 2 or greater)
7. Severe intraventricular haemorrhage (IVH; grade III/IV)
8. Moderate/severe neurodevelopmental disability, assessed by a standardised and validated assessment tool, a child developmental specialist, or both, at any age reported (outcome data grouped at 12, 18, and 24 months, if available)

###### Treatment of asymptomatic infants

1. Death or moderate/severe neurodevelopmental disability
2. Failure of PDA closure after completion of allocated treatment
3. Proportion of infants requiring surgical ligation or transcatheter occlusion
4. All‐cause mortality any time prior to NICU discharge
5. CLD, defined as oxygen requirement at 36 weeks’ postmenstrual age
6. Necrotising enterocolitis (NEC; stage 2 or greater
7. Severe intraventricular haemorrhage (IVH; grade III/IV)
8. Moderate/severe neurodevelopmental disability, assessed by a standardised and validated assessment tool, a child developmental specialist, or both, at any age reported (outcome data grouped at 12, 18, and 24 months, if available)

###### Treatment of symptomatic infants

1. Death or moderate/severe neurodevelopmental disability
2. Failure of PDA closure after completion of allocated treatment
3. Proportion of infants requiring surgical ligation or transcatheter occlusion
4. All‐cause mortality any time prior to NICU discharge
5. CLD, defined as oxygen requirement at 36 weeks’ postmenstrual age
6. Necrotising enterocolitis (NEC; stage 2 or greater)
7. Moderate/severe neurodevelopmental disability, assessed by a standardised and validated assessment tool, a child developmental specialist, or both, at any age reported (outcome data grouped at 12, 18, and 24 months, if available)

We reported the certainty of evidence as assessed by the review authors (who were in the best position to assess certainty given their familiarity with the study level data), using summary of findings tables from the reviews if provided.

The GRADE approach results in an assessment of the certainty of a body of evidence as one of four grades.

1. High certainty: further research is very unlikely to change our confidence in the estimate of effect
2. Moderate certainty: further research is likely to have an important impact on our confidence in the estimate of effect, and may change the estimate
3. Low certainty: further research is very likely to have an important impact on our confidence in the estimate of effect, and is likely to change the estimate
4. Very low certainty: we are very uncertain about the estimate

#### Data synthesis

We provided a narrative description of the characteristics of the included Cochrane Reviews. We then summarised the main results of the included reviews by categorising their findings, based on outcomes. We did not attempt to quantitatively synthesise the results using indirect comparison techniques, such as network meta‐analysis.

#### Subgroup analysis

If the information was available, we planned to separately report outcome results for the following subgroups.

1. Gestational age (less than 28 weeks, 28 weeks or more)
2. Birthweight (less than 1000 g, 1000 g or more)
3. Timing of initiation of treatment for asymptomatic PDA (less than 24 hours, 24 hours or longer)
4. Timing of initiation of treatment for symptomatic PDA (less than 72 hours, 72 hours or longer)
5. Method used to diagnose a symptomatic PDA (by echocardiographic criteria or only by clinical criteria)
6. Degree of haemodynamic significance of the PDA (based on echocardiographic criteria)

## Results

Our search (October 2022) identified 17 relevant Cochrane Reviews and two Cochrane Review protocols under review (Figure 1). Out of these 19 reviews, we included 16 reviews in this overview. The review by Mitra 2022 was identified to be a Bayesian network meta‐analysis of data obtained from studies, most of which were already included in the reviews of the respective pharmacoprophylactic interventions (Fowlie 2010; Jasani 2022; Ohlsson 2020a). Since the latter three reviews were already included in this overview, and to avoid duplication while summarising the results, we excluded the network meta‐analysis by Mitra 2022. One of the 16 reviews had no included studies and therefore did not contribute to the results (Anabrees 2011).

**1:**
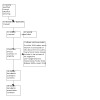
Study selection flow diagram

### Description of included reviews

We included the following reviews in this overview (Table 1).

**1:** Characteristics of included reviews

**Review ID and title**	**Date of search and date assessed as up‐to‐date**	**No. included trials (number of participants)**	**Types of studies**	**Types of participants**	**Types of Interventions**	**Relevant comparisons**
Anabrees 2011 Fluid restriction and prophylactic indomethacin vs prophylactic indomethacin alone for prevention of morbidity and mortality in extremely low birth weight infants	1966 to December 2010	None	RCTs and quasi‐RCTs	Infants, < 1000 g at birth, who received prophylactic indomethacin in the first 24 hours of life.	Fluid restriction (to achieve at least 10% weight loss in the first week of life) plus indomethacin prophylaxis (starting within the first 24 hours for 3 doses) versus indomethacin prophylaxis alone	Not applicable
Barrington 2002 Dopamine versus no treatment to prevent renal dysfunction in indomethacin‐treated preterm newborn infants	1966 to 2009	3 trials (75 infants)	RCTs and quasi‐RCTs	Preterm infants, ≤ 36 weeks' gestation at birth, receiving indomethacin for either PDA closure or prophylaxis, or prophylaxis against intraventricular haemorrhage, during the first month of life	Dopamine started before, simultaneously with, or after indomethacin administration	Dopamine started before, simultaneously with, or after indomethacin administration vs indomethacin only for: 1) infants given indomethacin as prophylaxis of intraventricular haemorrhage 2) infants given indomethacin as treatment for PDA
Bell 2014 Restricted versus liberal water intake for preventing morbidity and mortality in preterm infants	1966 to October 2014	5 trials (582 infants)	RCTs and quasi‐RCTs	Preterm infants, < 37 weeks' completed gestation	Fluid restriction defined as restriction of primarily or entirely intravenous fluid intake. No cutoff for restriction was specified.	Restricted fluid intake vs liberal fluid intake (standard or control therapy)
Bhola 2015 Chest shielding for prevention of a haemodynamically significant patent ductus arteriosus in preterm infants receiving phototherapy	1966 to March 2015	2 trials (128 infants)	RCTs, quasi‐RCTs, and cluster‐RCTs	Preterm infants. < 37 weeks' completed gestation, receiving phototherapy	Chest shielding with photo‐opaque material	1) Chest shielding with photo‐opaque material vs no shielding 2) chest shielding vs sham shielding (sham shielding defined as a simulated shield that is not photo‐opaque
Brion 2001 Furosemide for prevention of morbidity in indomethacin‐treated infants with patent ductus arteriosus	1966 to January 1998 Updated September 2003; April 2007	3 trials (70 participants)	RCTs	Preterm infants with a symptomatic patent ductus arteriosus who were to receive at least one dose of indomethacin	Infants randomly allocated to receive either indomethacin alone or indomethacin preceded by, or immediately followed with furosemide	1) Furosemide vs control (protocol including 1 to 3 doses) 2) Furosemide vs control (protocol including 3 doses)
Cooke 2003 Indomethacin for asymptomatic patent ductus arteriosus in preterm infants	1966 to September 2002	3 trials (97 participants)	RCTs	Preterm infants, born < 37 weeks' gestation, with asymptomatic PDA, who received treatment after 24 hours of age	Indomethacin administered either enterally or parenterally vs either placebo or no treatment	Indomethacin vs placebo
Evans 2021 Indomethacin for symptomatic patent ductus arteriosus in preterm infants	1946 to July 2020	14 trials (880 participants)	RCTs, quasi‐RCTs, cluster‐RCTs, and randomised cross‐over trials	Preterm infants, born at < 37 weeks' gestation, and low birth weight infants (< 2500 g), treated for symptomatic PDA, enrolled within the first 28 days of life	Indomethacin (any dose, any route) vs placebo or no treatment	1) Indomethacin vs placebo or control 2) Indomethacin vs placebo or control (sensitivity analysis)
Fowlie 2010 Prophylactic intravenous indomethacin for preventing mortality and morbidity in preterm infants	1966 to April 2010	19 trials (2872 participants)	RCTs and quasi‐RCTs	Preterm neonates, < 37 weeks’ completed gestation	Prophylactic (not guided by knowledge of PDA status) treatment with indomethacin given within 24 hours of birth vs placebo or no treatment. Specific dose regimens were not prespecified	Prophylactic indomethacin vs control
Görk 2008 Continuous infusion versus intermittent bolus doses of indomethacin for patent ductus arteriosus closure in symptomatic preterm infants	1966 to March 2007	2 trials (50 participants)	RCTs and quasi‐RCTs	Infants, < 37 weeks' estimated gestation, with a symptomatic PDA diagnosis made clinically, by echocardiography, or both, in the neonatal period (< 28 days)	*Experimental group:* continuous infusion of indomethacin after 24 hours of life (all doses and durations included) *Control group:* indomethacin administered as a bolus dose of no longer than 20 minutes in any dosing schedule after 24 hours of life	Continuous vs bolus indomethacin
Herrera 2007 Prolonged versus short course of indomethacin for the treatment of patent ductus arteriosus in preterm infants	1966 to December 2006	5 trials (431 participants)	RCTs and quasi‐RCTs	Preterm infants, < 37 weeks' completed gestation, with a PDA diagnosed on clinical, echocardiographic examination, or both	Indomethacin treatment by any route given as a long course (4 or more doses) or a short course 3 or fewer doses)	Prolonged‐ vs short‐course indomethacin
Jasani 2022 Paracetamol (acetaminophen) for patent ductus arteriosus in preterm or low birth weight infants	1946 to October 2021	27 trials (2278 participants)	RCTs and quasi‐RCTs	Infants born preterm (< 37 weeks' PMA) or with low birth weight (< 2500 g at birth) who had an echocardiographic diagnosis of a PDA, regardless of their postnatal age	Acetaminophen (given via any route for the purpose of closure of PDA) in any dose versus placebo or no intervention or versus another prostaglandin inhibitor.	1) Acetaminophen vs ibuprofen 2) Acetaminophen vs indomethacin 3) Prophylactic acetaminophen vs placebo or no intervention 4) Early acetaminophen versus placebo or no intervention 5) Late acetaminophen vs placebo or no intervention 6) Acetaminophen + ibuprofen vs ibuprofen + placebo or no intervention
Malviya 2013 Surgical versus medical treatment with cyclooxygenase inhibitors for symptomatic patent ductus arteriosus in preterm infants	2000 to February 2012	1 trial (154 participants)	RCTs and quasi‐RCTs	Preterm infants born at < 37 weeks' gestational age or low birth weight (< 2500 g) with symptomatic PDA, diagnosed either clinically or by ECHO criteria in the neonatal period (< 28 days)	Surgical ligation for closure of PDA vs medical treatment with cyclooxygenase inhibitors, each used as the initial treatment	Surgical vs medical treatment with indomethacin
Mitra 2020a Early treatment versus expectant management of haemodynamically significant patent ductus arteriosus for preterm infants	1980 to May 2019	14 trials (910 participants)	RCTs, quasi‐RCTs, and cluster‐RCTs	Preterm infants born at < 37 weeks' gestation or low birth weight infants (< 2500 g) with a haemodynamically significant PDA, diagnosed either clinically or via echocardiography, in the first 7 days of life	Early pharmacological treatment and expectant management for a haemodynamically significant PDA	1) Early treatment vs expectant management (no treatment in the first 7 days after birth) 2) Very early treatment (treatment by 72 hours of age) vs expectant management (no treatment in the first 72 hours after birth) 3) Very early treatment (treatment by 72 hours of age) vs early treatment (treatment by 7 days of age)
Mosalli 2008 Prophylactic surgical ligation of patent ductus arteriosus for prevention of mortality and morbidity in extremely low birth weight infants	1966 to December 2006 Updated March 2010	1 trial (84 participants)	RCTs and quasi‐RCTs	Infants born at < 28 weeks' gestation or less than 1000 g at birth who were on assisted ventilation with or without supplemental oxygen, without clinical signs of a haemodynamically significant PDA	Prophylactic surgical ligation of the patent ductus arteriosus (i.e. procedure done during first 72 hours of life in asymptomatic, extremely low birth weight infants) vs no prophylactic intervention or medical prophylaxis (cyclooxygenase inhibitors) without dose specification	1) Prophylactic surgical ligation vs no prophylactic treatment 2) Prophylactic surgical ligation vs prophylactic cyclooxygenase inhibitors
Ohlsson 2020a Ibuprofen for the prevention of patent ductus arteriosus in preterm and/or low birth weight infants	1966 to October 2018	9 trials (1070 participants)	RCTs and quasi‐RCTs	Preterm infants, < 37 weeks’ gestational age and low birth weight infants (< 2500 g), in their first 72 hours of life (3 days)	Prophylactic use of ibuprofen for prevention of PDA vs control (no intervention, placebo, or other cyclooxygenase inhibitor drugs (indomethacin, mefenamic acid), or rescue treatment with ibuprofen)	1) Ibuprofen (IV or oral) vs placebo or none 2) Ibuprofen (oral) vs placebo or none 3) Ibuprofen (IV) vs placebo or none 4) Ibuprofen (oral) vs indomethacin (oral)
Ohlsson 2020b Ibuprofen for the treatment of patent ductus arteriosus in preterm or low birth weight (or both) infants	1966 to November 2017	39 trials (2843 participants)	RCTs and quasi‐RCTs	Preterm infants, born < 37 weeks' gestational age or low birth weight (< 2500 g), with a PDA diagnosed either clinically or by echocardiographically‐guided criteria in the neonatal period (< 28 days)	Ibuprofen (given via any route for the purpose of closure of PDA) in any dose vs placebo, no intervention, or another prostaglandin inhibitor	1) IV ibuprofen vs placebo 2) Oral ibuprofen vs placebo 3) IV or oral ibuprofen vs IV or oral indomethacin 4) Oral ibuprofen vs IV or oral indomethacin 5) Oral ibuprofen vs IV ibuprofen 6) High‐dose oral or IV vs standard‐dose ibuprofen 7) Early vs expectant administration of IV ibuprofen 8) Echocardiography‐guided IV ibuprofen vs standard IV ibuprofen 9) Continuous infusion ibuprofen vs intermittent boluses ibuprofen 10) Rectal ibuprofen vs oral ibuprofen

ECHO: echocardiography; IV: intravenous; PDA: patent ductus arteriosus; PMA: postmenstrual age; RCT: randomised controlled trial; vs: versus

1. Anabrees 2011 (no included trials) included extremely low birthweight infants (< 1000 g at birth) who received prophylactic indomethacin in the first 24 hours of life, and compared fluid restriction (to achieve at least 10% weight loss in the first week of life) plus indomethacin prophylaxis (starting within the first 24 hours for three doses) versus indomethacin prophylaxis alone.
2. Barrington 2002 (3 RCTs, 75 infants) included preterm infants (≤ 36 weeks' gestation at birth) receiving indomethacin for either PDA closure or prophylaxis, or prophylaxis against intraventricular haemorrhage, during the first month of life. Given all three included trials compared dopamine with indomethacin versus dopamine alone for treatment of symptomatic PDA, we summarised the results from this review under 'Interventions for management of symptomatic PDA'.
3. Bell 2014 (5 trials, 582 infants) included predominantly preterm infants (< 37 weeks' completed gestation), and compared restricted fluid intake versus liberal fluid intake (standard or control therapy).
4. Bhola 2015 (2 RCTs, 128 infants) included preterm infants (< 37 weeks' completed gestation) receiving phototherapy, and compared chest shielding with photo‐opaque material versus no shielding, or chest shielding versus sham shielding (sham shielding defined as a simulated shield that is not photo‐opaque).
5. Brion 2001 (3 RCTs, 70 infants) included preterm infants with a symptomatic PDA who were to receive at least one dose of indomethacin, and compared indomethacin alone versus indomethacin preceded by, or immediately followed with furosemide.
6. Cooke 2003 (3 RCTs, 97 infants) included preterm infants (< 37 weeks' gestation) with an asymptomatic PDA who received treatment after 24 hours of age, and compared indomethacin administered either enterally or parenterally, versus placebo or no treatment.
7. Evans 2021 (14 RCTs, 880 infants) included preterm infants (< 37 weeks' gestational age) and low birthweight infants (< 2500 g) treated for symptomatic PDA, enroled within the first 28 days of life, and compared indomethacin (any dose, any route) versus placebo or no treatment.
8. Fowlie 2010 (19 RCTs, 2872 infants) included preterm infants (< 37 weeks' gestational age), and compared prophylactic (not guided by knowledge of PDA status) treatment with indomethacin given within 24 hours of birth versus either placebo or no treatment
9. Görk 2008 (2 RCTs, 50 infants) included preterm infants (< 37 weeks' estimated gestation) with a symptomatic PDA, diagnosed clinically, or by echocardiographic examination, or both, in the neonatal period (< 28 days), and compared continuous infusion of indomethacin versus indomethacin administered as a bolus dose of no longer than 20 minutes in any dosing schedule, after 24 hours of life, for closure of a symptomatic PDA.
10. Herrera 2007 (5 RCTs, 431 infants) included preterm infants (< 37 weeks' gestation) with a PDA diagnosed on clinical, or echocardiographic examination, or both, and compared indomethacin treatment by any route given as a long course (four or more doses) versus a short course (defined as three or fewer doses).
11. Jasani 2022 (27 RCTs, 2278 infants) included preterm infants (< 37 weeks' gestational age) and low birthweight infants (< 2500 g). The interventions included acetaminophen (given via any route for the purpose of closure of a PDA) administered alone or in combination, in any dose, versus placebo or no intervention, or versus another prostaglandin inhibitor. For prophylactic administration of acetaminophen, eligible infants were required to be within 24 hours of birth echocardiographic confirmation of PDA was not required. For therapeutic administration of acetaminophen, eligible infants were required to have an echocardiographic confirmation of the PDA, regardless of their postnatal age. This review is an update of the Ohlsson 2020 review, and is currently under editorial review.
12. Malviya 2013 (1 RCT, 154 infants) included preterm infants (< 37 weeks' gestational age) or low birthweight infants (< 2500 g) with a symptomatic PDA, diagnosed either clinically or by echocardiography in the neonatal period (less than 28 days), and compared surgical PDA ligation versus medical treatment with cyclooxygenase inhibitors, each used as the initial treatment.
13. Mitra 2020a (14 RCTs, 910 infants) included preterm (< 37 weeks' gestational age) or low birthweight infants (less than 2500 g) with a haemodynamically significant PDA diagnosed clinically or via echocardiography (or both) in the first seven days of life, and compared early treatment (treatment of a PDA by seven days of age) versus expectant management, and very early treatment (treatment of a PDA by 72 hours of age) versus expectant management.
14. Mosalli 2008 (1 RCT, 84 infants) included infants < 28 weeks' gestation or < 1000 g at birth who were on assisted ventilation, or supplemental oxygen, or both, without clinical signs of a haemodynamically significant PDA, and compared prophylactic surgical ligation of the PDA (i.e. procedure done during the first 72 hours) versus no prophylactic intervention or medical prophylaxis (cyclooxygenase inhibitors) without dose specification.
15. Ohlsson 2020a (9 RCTs, 1070 infants) included preterm infants (< 37 weeks' gestational age) and low birthweight infants (< 2500 g) in their first 72 hours of life (three days), and compared prophylactic use of ibuprofen for prevention of PDA versus control, consisting of no intervention, placebo, other cyclooxygenase inhibitor drugs (indomethacin, mefenamic acid), or rescue treatment with ibuprofen.
16. Ohlsson 2020b (39 RCTs, 2843 infants) included preterm infants (< 37 weeks' gestational age) or low birthweight infants (< 2500 g) with a PDA, diagnosed either clinically or by echocardiography in the neonatal period (less than 28 days), and compared ibuprofen (in different routes and dosages) versus indomethacin, other cyclo‐oxygenase inhibitor(s), placebo, or no intervention.

### Methodological quality of included reviews

The AMSTAR 2 assessment of the quality of the included reviews is presented in Table 2.

**2:** AMSTAR 2 assessment of the quality of the included reviews

**AMSTAR 2 question**	**Investigated review**															
	**Anabrees 2011**	**Barrington 2002**	**Bell 2014**	**Bhola 2015**	**Brion 2001**	**Cooke 2003**	**Evans 2021**	**Fowlie 2010**	**Görk 2008**	**Herrera 2007**	**Jasani 2022**	**Malviya 2013**	**Mitra 2020a**	**Mosalli 2008**	**Ohlsson 2020a**	**Ohlsson 2020b**
1. Did the research questions and inclusion criteria for the review include the components of PICO?	Yes	Yes	Yes	Yes	Yes	Yes	Yes	Yes	Yes	Yes	Yes	Yes	Yes	Yes	Yes	Yes
2. Did the report of the review contain an explicit statement that the review methods were established prior to the conduct of the review and did the report justify any significant deviations from the protocol?	Yes	Yes	Yes	Yes	Yes	Yes	Yes	Yes	Yes	Yes	Yes	Yes	Yes	Yes	Yes	Yes
3. Did the review authors explain their selection of the study designs for inclusion in the review?	No	No	No	No	No	No	No	No	No	No	No	No	No	No	No	No
4. Did the review authors use a comprehensive literature search strategy?	Yes	Yes	Yes	Yes	Yes	Yes	Yes	Yes	Yes	Yes	Yes	Yes	Yes	Yes	Yes	Yes
5. Did the review authors perform study selection in duplicate?	Yes	Yes	Yes	Yes	Yes	Yes	Yes	Yes	Yes	Yes	Yes	Yes	Yes	Yes	Yes	Yes
6. Did the review authors perform data extraction in duplicate?	Yes	Yes	Yes	Yes	Yes	Yes	Yes	Yes	Yes	Yes	Yes	Yes	Yes	Yes	Yes	Yes
7. Did the review authors provide a list of excluded studies and justify the exclusions?	Not applicable^a^	Yes	Yes	Yes	Yes	No	Yes	Yes	No	No	Yes	Yes	Yes	Yes	Yes	Yes
8. Did the review authors describe the included studies in adequate detail?	Not applicable^a^	Yes	Yes	Yes	Yes	Yes	Yes	Yes	Yes	Yes	Yes	Yes	Yes	Yes	Yes	Yes
9. Did the review authors use a satisfactory technique for assessing the risk of bias (RoB) in individual studies that were included in the review?	Not applicable^a^	Yes	Yes	Yes	No^b^	No^b^	Yes	Yes	Yes	No^b^	Yes	Yes	Yes	Yes	Yes	Yes
10. Did the review authors report on the sources of funding for the studies included in the review?	Not applicable^a^	No	No	No	No	No	No	No	No	No	No	No	Yes	Yes	No	No
11. If meta‐analysis was performed, did the review authors use appropriate methods for statistical combination of results?	Not applicable^a^	Yes	Yes	Yes	Yes	Yes	Yes	Yes	Yes	Yes	Yes	Yes	Yes	Yes	Yes	Yes
12. If meta‐analysis was performed, did the review authors assess the potential impact of RoB in individual studies on the results of the meta‐analysis or other evidence synthesis?	Not applicable^a^	No^c^	No^c^	Yes	Yes	Yes	Yes	No^c^	No^c^	No^c^	No^c^	Yes	Yes	Yes	No^c^	Yes
13. Did the review authors account for RoB in individual studies when interpreting/ discussing the results of the review?	Not applicable^a^	No^d^	No^d^	Yes	Yes	Yes	Yes	No^d^	Yes	No^d^	Yes	Yes	Yes	Yes	Yes	Yes
14. Did the review authors provide a satisfactory explanation for, and discussion of, any heterogeneity observed in the results of the review?	Not applicable^a^	Yes	Yes	Yes	Yes	Yes	Yes	Yes	Yes	Yes	Yes	Yes	Yes	Yes	Yes	Yes
15. If they performed quantitative synthesis, did the review authors carry out an adequate investigation of publication bias (small study bias) and discuss its likely impact on the results of the review?	Not applicable^a^	Yes	Yes	Yes	Yes	Yes	Yes	Yes	Yes	Yes	Yes	Yes	Yes	Yes	Yes	Yes
16. Did the review authors report any potential sources of conflict of interest, including any funding they received for conducting the review?	Yes	Yes	Yes	Yes	Yes	Yes	Yes	Yes	Yes	Yes	Yes	Yes	Yes	Yes	Yes	Yes
Overall Quality	Moderate	Low	Low	Moderate	Low	Critically low	Moderate	Low	Low	Critically low	Moderate	Moderate	High	High	Moderate	Moderate

^a^No studies included in the review ^b^ROB assessed only for one domain: allocation concealment (selection bias) ^c^Neither sensitivity analysis of low ROB studies provided nor explanation for omitting sensitivity analysis provided ^d^Neither discussion on impact of ROB of individual studies on the overall results provided nor GRADE certainty of evidence assessed

We assessed two reviews at high quality (Mitra 2020a; Mosalli 2008), seven reviews at moderate quality (Anabrees 2011; Bhola 2015; Evans 2021; Jasani 2022; Malviya 2013; Ohlsson 2020a Ohlsson 2020b), five at low quality (Barrington 2002; Bell 2014; Brion 2001; Fowlie 2010; Görk 2008), and two reviews at critically low quality (Cooke 2003; Herrera 2007).

Risk of bias in the included trials, as assessed by the respective review authors, is reported in Table 3. The certainty of the evidence for the primary outcomes of this overview (as available from the respective reviews) is summarised in Table 4 and Table 5.

**3:** Risk of bias of individual trials included in the reviews

**Review**	**Primary studies in the review**	**Risk of bias domains**						
		**Random** **sequence** **generation** **(selection** **bias)**	**Allocation concealment (selection bias)**	**Blinding of participants and personnel (performance bias)**	**Blinding of outcome assessment (detection bias)**	**Incomplete outcome data (attrition bias)**	**Selective reporting (reporting bias)**	**Other bias**
Barrington 2002	Baenziger 1999	Unclear	Unclear	High	High	Unclear	NA	NA
	Fajardo 1992	Unclear	Unclear	Low	Unclear	Low	NA	NA
	Seri 1984	Low	Low	High	High	High	NA	NA
Bell 2014	Bell 1980	Low	Low	High	High	Low	Low	NA
	Kavvadia 2000	Unclear	Unclear	High	High	NA	NA	NA
	Lorenz 1982	Unclear	Unclear	High	High	High	NA	NA
	Tammela 1992	Unclear	Low	High	High	Unclear	NA	NA
	von Stockhausen 1980	Unclear	Unclear	High	High	Unclear	NA	NA
Bhola 2015	Rosenfeld 1986	Low	High	High	High	Unclear	Low	High
	Travadi 2006	Low	Low	High	Low	Low	Low	High
Brion 2001	Romagnoli 1997	NA	Unclear	NA	NA	NA	NA	NA
	Vargas‐Origel 1986	NA	Unclear	NA	NA	NA	NA	NA
	Yeh 1982	NA	Low	NA	NA	NA	NA	NA
Cooke 2003	Hammerman 1987	NA	Low	NA	NA	NA	NA	NA
	Mahony 1982	NA	Low	NA	NA	NA	NA	NA
	Weesner 1987	NA	Low	NA	NA	NA	NA	NA
Evans 2021	Cotton 1980	Unclear	Unclear	High	Unclear	Low	Unclear	Unclear
	Gersony 1983	Low	Low	Low	Low	Unclear	Unclear	Low
	Kluckow 2014	Low	Low	Low	Low	Low	Low	Unclear
	Knight 2011	Unclear	Unclear	Unclear	Unclear	Unclear	Unclear	Unclear
	Krauss 1989	Low	Unclear	High	Unclear	Unclear	Unclear	Low
	Merritt 1981	Low	Unclear	High	High	High	Unclear	Unclear
	Monset‐Couchard 1983	Unclear	High	High	Unclear	Low	Unclear	Unclear
	Nestrud 1980	Low	Low	Low	Unclear	Low	Unclear	Low
	Neu 1981	Unclear	Unclear	Low	Low	Low	Unclear	Low
	Osborn 2003	Unclear	Low	Low	Unclear	Unclear	Unclear	Low
	Rudd 1983	Low	Unclear	Low	Unclear	Low	Unclear	Low
	Valaes 1980	Unclear	Unclear	Unclear	Unclear	Low	Unclear	Low
	Yanagi 1981	Low	Low	Low	Low	Low	Unclear	Low
	Yeh 1981	Low	Low	Low	Low	Low	Unclear	Low
Fowlie 2010	Bada 1989	NA	NA	NA	Low	Low	NA	NA
	Bandstra 1988	NA	Low	NA	Low	Low	NA	NA
	Couser 1996	Low	Low	NA	Low	Low	NA	NA
	Domanico 1994	NA	NA	NA	Low	Low	NA	NA
	Gutierrez 1987	Low	Low	NA	Low	Low	NA	NA
	Hanigan 1988	Low	Low	NA	Low	Low	NA	NA
	Krueger 1987	NA	NA	NA	High	Low	NA	NA
	Mahony 1985	Low	Low	NA	Low	Low	NA	NA
	Ment 1985	Low	Low	NA	Low	Low	NA	NA
	Ment 1988	Low	Low	NA	Low	Low	NA	NA
	Ment 1994a	Low	Low	NA	Low	Low	NA	NA
	Ment 1994b	Low	Low	NA	Low	Low	NA	NA
	Morales‐Suarez 1994	NA	NA	NA	Low	Low	NA	NA
	Puckett 1985	NA	NA	NA	Unclear	High	NA	NA
	Rennie 1986	NA	NA	NA	Unclear	Low	NA	NA
	Supapannachart 1999	Low	Low	NA	Low	Low	NA	NA
	Schmidt 2001	Low	Low	NA	Low	Low	NA	NA
	Vincer 1987	NA	NA	NA	Low	Low	NA	NA
	Yaseen 1997	Low	Low	NA	Low	Low	NA	NA
Görk 2008	Christmann 2002	NA	Unclear	NA	Unclear	High	NA	NA
	Hammerman 1995	NA	Unclear	NA	Low	High	NA	NA
Herrera 2007	Hammerman 1990	NA	Low	NA	NA	NA	NA	NA
	Lee 2003	NA	Low	NA	NA	NA	NA	NA
	Rennie 1991	NA	Low	NA	NA	NA	NA	NA
	Rhodes 1988	NA	Unclear	NA	NA	NA	NA	NA
	Tammela 1999	NA	Unclear	NA	NA	NA	NA	NA
Jasani 2022	Al‐Lawama 2017	Low	Low	High	Unclear	Low	Unclear	Low
	Asadpour 2018	High	Low	Unclear	Unclear	Low	Low	Low
	Asbagh 2015	Low	Unclear	Unclear	Unclear	Low	Unclear	Low
	Babaei 2018	Unclear	Unclear	Unclear	Unclear	Low	Low	Low
	Bagheri 2016	Low	Unclear	High	Low	High	Unclear	Low
	Bagheri 2018	Low	High	High	Low	Low	Low	Low
	Balachander 2020	Low	Low	High	Low	Low	Unclear	Low
	Dang 2013	Low	Low	High	High	Low	Low	Low
	Dani 2021	Low	Low	High	Low	Low	Low	Low
	Dash 2015	Low	Low	High	High	Low	Unclear	Low
	Davidson 2021	Low	Low	High	Low	Low	Low	Low
	El‐Farrash 2019	Low	Low	Low	Low	Low	Low	Low
	El‐Mashad 2017	Low	Low	High	Low	Low	Unclear	Low
	Ghaderian 2019a	High	Unclear	High	Low	Low	Low	Low
	Ghaderian 2019b	Low	Unclear	High	Low	Low	Low	Low
	Härkin 2016	Low	Low	Low	Low	Low	Low	Low
	Hochwald 2018	Low	Low	Low	Low	Low	Low	Low
	Jafari 2019	Low	Unclear	Unclear	Unclear	Low	Low	Low
	Kluckow 2019	Low	Low	Low	Low	Low	Low	Low
	Kumar 2020	Low	Low	Low	Low	Low	Low	Low
	Meena 2020	Unclear	Unclear	Unclear	Unclear	Low	Unclear	Low
	Oboodi 2020	Low	Unclear	Unclear	Unclear	Low	Low	Low
	Oncel 2014	Low	Low	High	High	Low	Low	Low
	Schindler 2021	Low	Low	Low	Unclear	Low	Low	Low
	Shahmirzadi 2021	Low	Unclear	Unclear	Unclear	Low	Unclear	Low
	Tauber 2020	Unclear	Unclear	High	Unclear	Low	Low	Low
	Yang 2016	Low	Unclear	High	Unclear	Low	Unclear	Low
Malviya 2013	Gersony 1983	Low	Low	High	High	Low	Unclear	Low
Mitra 2020a	Bagnoli 2013	Unclear	Unclear	Unclear	Unclear	Low	Unclear	Low
	CTRI/2009/091/000041	Low	Low	High	High	Unclear	Unclear	Unclear
	De Waal 2019	Low	Low	Low	Low	Low	Low	Low
	El‐Khuffash 2021	Low	Low	Low	Low	Low	Low	Low
	Gersony 1983	Low	Low	Low	Low	Low	Unclear	Low
	Ghanem 2010	High	High	Unclear	Unclear	Low	Unclear	Low
	Kaapa 1983	Unclear	Low	High	High	High	Unclear	Unclear
	Kluckow 2014	Low	Unclear	Low	Low	Low	Low	Unclear
	Knight 2011	Unclear	Unclear	Unclear	Unclear	Unclear	Unclear	Unclear
	Krauss 1989	Low	Unclear	High	High	Low	Unclear	Low
	Lin 2012	Low	Low	Unclear	Unclear	Low	Unclear	Low
	Merritt 1981	Low	Unclear	High	High	Unclear	Unclear	High
	Sosenko 2012	Low	Low	Low	Low	Low	Low	Unclear
	Van Overmeire 2001	Unclear	Low	High	High	Low	Unclear	Low
Mosalli 2008	Cassady 1989	NA	Low	NA	Low	Low	NA	NA
*Ohlsson 2020a	Dani 2000	Unclear	Low	High	High	Low	Unclear	Low
	Dani 2005	Unclear	Low	Low	Low	Low	Unclear	Low
	De Carolis 2000	Unclear	Unclear	High	High	Low	Unclear	Low
	Gournay 2004	Unclear	Low	Low	Low	Low	Unclear	Low
	Kalani 2016	Unclear	Unclear	Unclear	Unclear	Low	Unclear	Low
	Kanmaz 2013	Low	Low	High	High	Low	Unclear	Low
	Sangtawesin 2006	Unclear	Unclear	Low	Low	Low	Unclear	Low
	Sangtawesin 2008	Unclear	Unclear	Low	Low	Low	Unclear	Low
	Van Overmeire 2004	Low	Low	Low	Low	Low	Unclear	Unclear
Ohlsson 2020b	Adamska 2005	Unclear	Low	Low	Low	Low	Unclear	Low
	Akar 2017	Unclear	Low	High	High	Low	Unclear	Low
	Akisu 2001	Unclear	Unclear	High	High	Low	Unclear	Low
	Aly 2007	Unclear	Low	High	Low	Low	Unclear	Low
	Aranda 2009	Low	Low	Low	Low	Unclear	Unclear	Low
	Bagnoli 2013	Unclear	Unclear	Unclear	Unclear	Low	Unclear	Low
	Bravo 2014	Low	Low	High	Low	Low	Unclear	Low
	Cherif 2008	Unclear	Low	High	Low	Low	Low	Low
	Chotigeat 2003	Unclear	Unclear	High	High	Low	Unclear	Low
	Dani 2012	Unclear	Low	Unclear	Low	Low	Low	Low
	Demir 2016	Unclear	Unclear	High	High	Low	Unclear	Low
	Ding 2014	Unclear	Unclear	Unclear	Low	Low	Unclear	Unclear
	El‐Mashad 2017	Low	Low	High	Low	Low	Unclear	Low
	Erdeve 2012	Unclear	Low	High	Low	High	Low	Low
	Fakhraee 2007	Unclear	Unclear	Unclear	Low	Low	Unclear	Low
	Fesharaki 2012	Unclear	Unclear	Unclear	Unclear	Low	Unclear	Low
	Gimeno 2005	Low	Low	High	Unclear	Low	Unclear	Low
	Gokmen 2011	Unclear	Low	High	Low	Low	Unclear	Low
	Hammerman 2008	Low	Unclear	High	Low	Low	Low	Low
	Lago 2002	Unclear	Low	High	High	Low	Unclear	Low
	Lago 2014	Low	Low	High	Unclear	Low	Unclear	Low
	Lin 2012	Unclear	Unclear	Unclear	Unclear	Unclear	Unclear	Unclear
	Lin 2017	Low	Low	Unclear	Low	High	Unclear	Low
	Mosca 1997	Unclear	Unclear	High	Unclear	Low	Unclear	Low
	Patel 1995	Unclear	Unclear	High	High	Low	Unclear	Low
	Patel 2000	Unclear	Low	Low	Low	Low	Unclear	Low
	Pezzati 1999	Unclear	Unclear	Unclear	Unclear	Low	Unclear	Low
	Pistulli 2014	Unclear	Unclear	High	High	High	Unclear	Unclear
	Plavka 2001	Unclear	Unclear	Unclear	Unclear	Low	Unclear	Low
	Pourarian 2008	Unclear	High	High	High	Low	Unclear	Low
	Pourarian 2015	Unclear	Low	Unclear	Low	Low	Unclear	Low
	Salama 2008	Low	Unclear	High	High	Low	Unclear	Low
	Sosenko 2012	Low	Low	Low	Low	Low	Low	Low
	Su 2003	Unclear	Unclear	High	Low	Low	Unclear	Low
	Su 2008	Low	Low	Low	Low	Low	Unclear	Low
	Supapannachart 2002	Unclear	Low	High	High	Low	Unclear	Low
	Van Overmeire 1997	Unclear	Low	High	Unclear	Low	Unclear	Low
	Van Overmeire 2000	Unclear	Low	High	Low	Low	Unclear	Low
	Yadav 2014	Low	Low	High	High	Low	Unclear	Low

*Both blinding domains (performance and detection bias) were grouped together as “Blinding (performance bias and detection bias).” NA: Not assessed in the review

**4:** Summary of findings – interventions for prevention of PDA and related complications in preterm infants

**Outcomes**	**Intervention vs comparison** **(Review, year)**	**Anticipated absolute effects (95% CI)**		**Relative risk** **(95% CI)**	**No. of participants** **(studies)**	**Certainty** **of the evidence** **(GRADE)**
		**Risk with comparison**	**Risk with intervention**			
**PDA confirmed on echocardiogram**	Ibuprofen vs control (Ohlsson 2020a)	424 per 1000	166 per 1000 (132 to 204)	RR 0.39 (0.31 to 0.48)	1029 (9)	Moderate^a^
	Acetaminophen vs control (Jasani 2022)	612 per 1000	165 per 1000 (110 to 257)	RR 0.27 (0.18 to 0.42)	240 (3)	Low^b^
**Invasive PDA closure**	Ibuprofen vs control (Ohlsson 2020a)	43 per 1000	20 per 1000 (9 to 41)	RR 0.46 (0.22 to 0.96)	925 (7)	Moderate^a^
**All‐cause mortality**	Acetaminophen vs control (Jasani 2022)	91 per 1000	54 per 1000 (22 to 131)	RR 0.59 (0.24 to 1.44)	240 (3)	Low^c^
**Necrotising enterocolitis**	Ibuprofen vs control (Ohlsson 2020a)	64 per 1000	61 per 1000 (39 to 96)	RR 0.96 (0.61 to 1.50)	1028 (9)	Moderate^a^
**Severe intraventricular haemorrhage (grade III/IV)**	Ibuprofen vs control (Ohlsson 2020a)	114 per 1000	76 per 1000 (51 to 114)	RR 0.67 (0.45 to 1.00)	925 (7)	Moderate^a^

^a^Downgraded one level due to serious risk of bias ^b^Downgraded two levels due to serious risk of bias and high heterogeneity ^c^Downgraded two levels due to very serious risk of bias **CI**: confidence interval; **PDA**: patent ductus arteriosus; **RR**: risk ratio; **vs**: versus

**5:** Summary of findings – interventions for management of symptomatic (haemodynamically significant) PDA in preterm infants

**Outcomes**	**Intervention vs comparison** **(Review, year)**	**Anticipated absolute effects (95% CI)**		**Relative effect** **(95% CI)**	**No. of participants** **(studies)**	**Certainty** **of the evidence** **(GRADE)**
		**Risk with comparison**	**Risk with intervention**			
**Failure of PDA closure after completion of allocated treatment**	Indomethacin vs placebo/ no treatment (Evans 2021)	732 per 1000	220 per 1000 (168 to 278)	RR 0.30 (0.23 to 0.38)	654 (10)	High
	IV Ibuprofen vs placebo/ no treatment (Ohlsson 2020b)	471 per 1000	294 per 1000 (29 to 432)	RR 0.62 (0.44 to 0.86)	206 (2)	Moderate^a^
	Ibuprofen vs indomethacin (Ohlsson 2020b)	280 per 1000	305 per 1000 (0 to 708)	RR 1.07 (0.92 to 1.24)	1590 (24)	Moderate^a^
	Oral ibuprofen vs indomethacin (Ohlsson 2020b)	386 per 1000	393 per 1000 (0 to 708)	RR 0.96 (0.73 to 1.27)	272 (8)	Low^b^
	Oral ibuprofen vs IV ibuprofen (Ohlsson 2020b)	363 per 1000	139 per 1000 (115 to 156)	RR 0.38 (0.26 to 0.56)	406 (5)	Moderate^a^
	High‐dose vs standard dose ibuprofen (Ohlsson 2020b)	411 per 1000	147 per 1000 (0 to 300)	RR 0.37 (0.22 to 0.61)	190 (3)	Moderate^a^
	Acetaminophen vs ibuprofen (Jasani 2022)	299 per 1000	308 per 1000 (266 to 355)	RR 1.02 (0.88 to 1.18)	1535 (18)	Moderate^a^
	Acetaminophen vs indomethacin (Jasani 2022)	297 per 1000	303 per 1000 (232 to 395)	RR 1.02 (0.78 to 1.33)	380 (4)	Low^c^
	Early acetaminophen vs placebo (Jasani 2022)	790 per 1000	277 per 1000 (182 to 419)	RR 0.35 (0.23 to 0.53)	127 (2)	Low^c^
	Late acetaminophen vs placebo (Jasani 2022)	1000 per 1000	850 per 1000 (720 to 1000)	RR 0.85 (0.72 to 1.01)	55 (1)	Low^d^
	Ibuprofen plus acetaminophen vs Ibuprofen plus placebo or no intervention (Jasani 2022)	313 per 1000	241 per 1000 (134 to 425)	RR 0.77 (0.43 to 1.36)	111 (2)	Low^e^
**Invasive PDA closure**	Indomethacin vs placebo/ no treatment (Evans 2021)	113 per 1000	75 per 1000 (37 to 146)	RR 0.66 (0.33 to 1.29)	275 (7)	Moderate^f^
	Ibuprofen vs indomethacin (Ohlsson 2020b)	135 per 1000	144 per 1000 (0 to 250)	RR 1.06 (0.81 to 1.39)	1275 (16)	Moderate^a^
	Oral ibuprofen vs indomethacin (Ohlsson 2020b)	188 per 1000	181 per 1000 (0 to 250)	RR 0.93 (0.50 to 1.74)	174 (4)	Low^b^
	Oral ibuprofen vs IV ibuprofen (Ohlsson 2020b)	51 per 1000	19 per 1000 (0 to 31)	RR 0.41 (0.41 to 1.21)	406 (5)	Moderate^a^
**All‐cause mortality**	Indomethacin vs placebo/ no treatment (Evans 2021)	164 per 1000	128 per 1000 (75 to 217)	RR 0.78 (0.46 to 1.33)	314 (8)	Moderate^f^
	Acetaminophen vs ibuprofen (Jasani 2022)	166 per 1000	181 per 1000 (133 to 245)	RR 1.09 (0.80 to 1.48)	734 (8)	Moderate^a^
	Acetaminophen vs indomethacin (Jasani 2022)	186 per 1000	160 per 1000 (73 to 358)	RR 0.86 (0.39 to 1.92)	114 (2)	Low^e^
**Chronic lung disease (oxygen requirement at 36 weeks’ postmenstrual age)**	Indomethacin vs placebo/ no treatment (Evans 2021)	313 per 1000	250 per 1000 (128 to 484)	RR 0.80 (0.41 to 1.55)	92 (1)	Low^g^
**Necrotising enterocolitis**	Indomethacin vs placebo/ no treatment (Evans 2021)	53 per 1000	68 per 1000 (19 to 243)	RR 1.27 (0.36 to 4.55)	147 (2)	Low^g^
	IV ibuprofen vs placebo/ no treatment (Ohlsson 2020b)	68 per 1000	129 per 1000 (119 to 139)	RR 1.84 (0.87 to 3.90)	264 (2)	Moderate^a^
	Ibuprofen vs indomethacin (Ohlsson 2020b)	111 per 1000	73 per 1000 (0 to 400)	RR 0.68 (0.49 to 0.94)	1292 (18)	Moderate^a^
	^a^Oral ibuprofen vs indomethacin (Ohlsson 2020b)	224 per 1000	83 per 1000 (0 to 400)	RR 0.41 (0.23 to 0.73)	249 (7)	Low^b^
	High‐dose vs standard dose ibuprofen (Ohlsson 2020b)	123 per 1000	123 per 1000 (114 to 133)	RR 1.00 (0.40 to 2.50)	130 (2)	Low^e^
	Acetaminophen vs ibuprofen (Jasani 2022)	70 per 1000	91 per 1000 (61 to 136)	RR 1.30 (0.87 to 1.94)	1015 (10)	Moderate^a^
	Acetaminophen vs indomethacin (Jasani 2022)	93 per 1000	39 per 1000 (18 to 89)	RR 0.42 (0.19 to 0.96)	384 (4)	Low^e^
	Late Acetaminophen vs placebo (Jasani 2022)	36 per 1000	37 per 1000 (3 to 563)	RR 1.04 (0.07 to 15.76)	55 (1)	Low^d^
	Ibuprofen plus acetaminophen vs Ibuprofen plus placebo or no intervention (Jasani 2022)	83 per 1000	28 per 1000 (1 to 621)	RR 0.33 (0.01 to 7.45)	24 (1)	Low^e^

^a^Downgraded one level due to serious risk of bias ^b^Downgraded two levels due to very serious risk of bias ^c^Downgraded two levels due to serious risk of bias and high heterogeneity ^d^Downgraded two levels due to imprecision and indirectness ^e^Downgraded two levels due to serious risk of bias and imprecision ^f^Downgraded one level due to serious imprecision ^g^Downgraded two levels due to very serious imprecision **CI**: confidence interval; **PDA**: patent ductus arteriosus; **RR**: risk ratio; **vs**: versus

### Effect of interventions

#### Interventions (pharmacological or surgical) for prevention of PDA and related complications in preterm infants

The results for the following outcomes are summarised in Table 6

**6:** Interventions (pharmacological or surgical) for prevention of PDA and related complications in preterm infants

**Interventions** ** **			**Prophylactic indomethacin**	**Prophylactic ibuprofen**	**Prophylactic acetaminophen**	**Prophylactic surgical ligation**	**Chest shielding during phototherapy**	**Restriction of fluid intake**
**Outcomes**								
**Severe intraventricular haemorrhage**			0.66, 95% CI 0.53 to 0.82; 14 RCTs, N = 2588	0.67, 95% CI 0.45 to 1.00; 7 RCTs, N = 925; moderate‐certainty evidence	1.0, 95% CI 9 0.07 to 16.39; 1 RCT, N = 48	0.81, 95% CI 0.52, to 1.28; 1 RCT, N = 76	0.64, 95% CI 0.22 to 1.85; 2 RCTs, N = 128	‐
**Death or moderate/severe neurodevelopmental disability**			1.02, 95% CI 0.90 to 1.15; 3 RCTs, N = 1491	‐	‐	‐	‐	‐
**PDA confirmed on echocardiogram**			0.29, 95% CI 0.22 to 0.38; 7 RCTs, N = 965	0.39, 95% CI 0.31 to 0.48; 9 RCTs, N = 1029	0.27, 95% CI 0.18 to 0.42; 3 RCTs, N = 240	‐	0.92, 95% CI 0.52 to 1.64; 1 RCT, N = 54	0.52, 95% CI 0.37 to 0.73; 4 RCTs, N = 526
**Haemodynamically significant PDA**			‐	‐	‐	‐	0.23, 95% CI 0.05 to 1.01; 1 RCT, N = 74	‐
**Need for open‐label medical treatment**			‐	0.17, 95% CI 0.11 to 0.26; 6 RCTs, N = 776	‐	‐	0.12, 95% CI 0.02 to 0.88; 1 RCT, N = 74	‐
**Need for surgical ligation or transcatheter occlusion**			0.51, 95% CI 0.37 to 0.71; 8 RCTs, N = 1791	0.46, 95% CI 0.22 to 0.96; 7 RCTs, N = 925	‐	‐	0.35, 95% CI 0.01 to 8.36; 1 RCT, N = 74	‐
**Chronic lung disease**	**Oxygen at 28 postnatal days**		1.08, 95% CI 0.92 to 1.26; 9 RCTs, N = 1022	0.88, 95% CI 0.32 to 2.42; 1 RCT, N = 41	0.69, 95% CI 0.32 to 1.48; 1 RCT, N = 48	‐	‐	‐
	**Oxygen at 36 weeks' PMA**		1.06, 95% CI 0.92 to 1.22; 1 RCT, N = 999	1.06, 95% CI 0.89 to 1.26; 5 RCTs, N = 817	0.36, 95% CI 0.02 to 8.45; 1 RCT, N = 48	1.07, 95% CI 0.68 to 1.69; 1 RCT, N = 48		0.85, 95% CI 0.63 to 1.14; 4 RCTs, N = 526
	**Unspecified age at diagnosis**		‐	0.94, 95% CI 0.51 to 1.72; 2 RCTs, N = 99	‐	‐		‐
**Intraventricular haemorrhage (any degree)**			0.88, 95% CI 0.80 to 0.98; 14 RCTs, N = 2532	0.96, 95% CI 0.78 to 1.17); 6 RCTs, N = 901	‐	‐	0.53, 95% CI 0.10 to 2.71; 1 RCT, N = 74	0.74, 95% CI 0.48 to 1.14; 3 RCTs, N = 356
**Pulmonary haemorrhage**			0.84, 95% CI 0.66 to 1.07; 4 RCTs, N = 1591	‐	‐	‐	‐	‐
**Retinopathy of prematurity**	**Any stage**		1.02, 95% CI 0.92 to 1.12; 5 RCTs, N = 1571	1.01, 95% CI 0.73 to 1.38; 5 RCTs, N = 369	‐	0.67, 95% CI 0.31 to 1.43; 1 RCT, N = 43	0.53, 95% CI 0.10 to 2.71; 1 RCT, N = 74	‐
	**Severe/requiring treatment**		1.75, 95% CI 0.92 to 3.34; 2 RCTs, N = 289	‐	3.25, 95% CI 0.14 to 76.01; 1 RCT, N = 48	0.32, 95% CI 0.04 to 2.82; 1 RCT, N = 43	‐	‐
**Duration of hospitalisation (days)**			‐	MD 1.30 days, 95% CI ‐3.07 to 5.67; 6 RCTs, N = 447	‐	‐	MD ‐8.05 days, 95% CI ‐18.04 to 1.94;2 RCTs, N = 128	‐
**Moderate/severe neurodevelopmental disability**			0.96, 95% CI 0.79 to 1.17; 3 RCTs, N = 1286	‐	‐	‐	‐	‐
**All‐cause mortality**		**Mortality during hospital stay**	0.82, 95% CI 0.65 to 1.03; 17 RCTs, N = 1567	0.90, 95% CI 0.62 to 1.30; 4 RCTs, N = 700	0.59, 95% CI 0.24 to 1.44; 3 RCTs, N = 240; low‐certainty evidence	‐	1.68, 95% CI 0.75 to 3.78; 2 RCTs, N = 128	0.81, 95% CI 0.54 to 1.23; 5 RCTs, N = 582
		**Neonatal mortality (< 28 days)**	‐	0.93, 95% CI 0.50 to 1.74; 6 RCTs, N = 342	‐	0.88, 95% CI 0.53 to 1.45; 1 RCT, N = 84	1.06, 95% CI 0.16 to 7.10; 1 RCT, N = 74	‐
		**Mortality at 1‐year, or at latest follow‐up**	0.96, 95% CI 0.81 to 1.12; 18 RCTs, N = 2769	‐	‐	1.06, 95% CI 0.75 to 1.49; 1 RCT, N = 84	‐	‐
		**Mortality before 36 weeks' postmenstrual age**	‐	0.96, 95% CI 0.56 to 1.66; 1 RCT, N = 131	‐	‐	‐	‐
**Necrotising enterocolitis**			1.09, 95% CI 0.82 to 1.46; 12 RCTs, N = 2401	0.96, 95% CI 0.61 to 1.50; 9 RCTs, N = 1028	0.36, 95% CI 0.02 to 8.45; 1 RCT, N = 48	0.25, 95% CI 0.08 to 0.83; 1 RCT, N = 84	‐	0.43, 95% CI 0.21 to 0.87; 4 RCTs, N = 526
**Gastrointestinal perforation**			1.13, 95% CI 0.71 to 1.79; 1 RCT, N = 1202	4.88, 95% CI 0.87 to 27.36; 2 RCTs, N = 167	‐	‐	‐	‐
**Gastrointestinal bleeding**			‐	2.05, 95% CI 1.19 to 3.51; 5 RCTs, N = 282; low‐certainty evidence	‐	‐	‐	‐
**Oliguria**			1.90, 95% CI 1.45 to 2.47; 8 RCTs, N = 2115	1.45, 95% CI 1.04 to 2.02; 4 RCTs, N = 747; high‐certainty evidence	0.78, 95% CI 0.29 to 2.11; 1 RCT, N = 48	‐	‐	‐
**Serum/plasma levels of creatinine after treatment**			‐	WMD* 0.09, 95% CI 0.05 to 0.13; 6 RCTs, N = 800	‐	‐	‐	‐
**Increase in serum/plasma creatinine after treatment**			1.09, 95% CI 0.47 to 1.79; 4 RCTs, N = 618	3.70, 95% CI 1.05 to 12.98; 2 RCTs, N = 285	‐	‐	‐	‐
**Serum/plasma bilirubin after treatment**			‐	‐	MD 1 µmol/L, 95% CI ‐10.35 to 12.35; 1 RCT, N = 48	‐	‐	‐

**CI**: confidence interval; **MD**: mean difference; **PDA**: patent ductus arteriosus; **PMA**: post menstrual age; **RCT**: randomised controlled trials; **WMD**: weighted mean difference Comparisons presented are intervention versus placebo. Data presented as risk ratio (RR), unless otherwise specified; certainty of evidence added when available from review. *WMD presented as reported in the respective review

##### Severe intraventricular haemorrhage (IVH; grade III/IV)

Five Cochrane Reviews reported on the outcome of severe IVH. They included the following interventions.

###### Prophylactic indomethacin

Prophylactic indomethacin versus placebo: the review by Fowlie 2010 showed that compared to control, prophylactic indomethacin reduced severe IVH (risk ratio (RR) 0.66, 95% confidence interval (CI) 0.53 to 0.82; 14 RCTs, 2588 infants).

###### Prophylactic ibuprofen

Prophylactic ibuprofen versus placebo: the review by Ohlsson 2020a showed that compared to placebo or no intervention, prophylactic ibuprofen possibly reduced severe IVH (RR 0.67, 95% CI 0.45 to 1.00; 7 RCTs, 925 infants; moderate‐certainty evidence).

###### Prophylactic acetaminophen

Prophylactic acetaminophen versus placebo: the review by Jasani 2022 showed that there was no evidence of a difference between prophylactic acetaminophen and placebo or no intervention for severe IVH (RR 1.09, 95% CI 0.07 to 16.39; 1 RCT, 48 infants).

###### Prophylactic surgical PDA ligation

Prophylactic surgical PDA ligation versus control (prophylactic cyclooxygenase inhibitor drugs only): the review by Mosalli 2008 showed that there was no evidence of a difference between prophylactic surgical PDA ligation and control (prophylactic cyclooxygenase inhibitor drugs) for severe IVH (RR 0.81, 95% CI 0.52 to 1.28; 1 RCT, 76 infants).

###### Chest shielding during phototherapy

Chest shielding during phototherapy versus control: the review by Bhola 2015 showed that there was no evidence of a difference between chest shielding during phototherapy and control for severe IVH (RR 0.64, 95% CI 0.22 to 1.85; 2 RCTs, 128 infants).

##### Death or moderate/severe neurodevelopmental disability

Only one Cochrane Review reported on the composite outcome of death or moderate/severe neurodevelopmental disability. It included the following intervention.

###### Prophylactic indomethacin

Prophylactic indomethacin versus placebo: the review by Fowlie 2010 showed that there was no evidence of a difference between prophylactic indomethacin and control for the composite outcome of death or moderate/severe neurodevelopmental disability (RR 1.02, 95% CI 0.90 to 1.15; 3 RCTs, 1491 infants).

##### PDA confirmed on echocardiogram

Five Cochrane Reviews reported on the outcome of echocardiogram‐confirmed PDA post‐prophylaxis. They included the following interventions.

###### Prophylactic indomethacin

Prophylactic indomethacin versus placebo: the review by Fowlie 2010 showed that compared to control, prophylactic indomethacin reduced the presence of PDA (RR 0.29, 95% CI 0.22 to 0.38; 7 RCTs, 965 infants).

###### Prophylactic ibuprofen

Prophylactic ibuprofen versus placebo: the review by Ohlsson 2020a showed that compared to placebo or no intervention, prophylactic ibuprofen reduced the presence of PDA (RR 0.39, 95% CI 0.31 to 0.48; 9 RCTs, 1029 infants; moderate‐certainty evidence).

###### Prophylactic acetaminophen

Prophylactic acetaminophen versus placebo: the review by Jasani 2022 showed that compared to placebo or no intervention, prophylactic acetaminophen reduced the presence of PDA (RR 0.27, 95% CI 0.18 to 0.42; 3 RCTs, 240 infants).

###### Chest shielding during phototherapy

Chest shielding during phototherapy versus control: the review by Bhola 2015 showed that there was no evidence of a difference between chest shielding during phototherapy and control for any PDA detected by echocardiography (RR 0.92, 95% CI 0.52 to 1.64; 1 RCT, 54 infants), or detection of a haemodynamically significant PDA (RR 0.23, 95% CI 0.05 to 1.01; 1 RCT, 74 infants).

###### Restriction of fluid intake

Restricted versus liberal fluid intake: the review by Bell 2014 showed that compared to liberal fluid intake, restriction of predominantly intravenous (IV) fluids reduced the persistence of PDA (RR 0.52, 95% CI 0.37 to 0.73; 4 RCTs, 526 infants).

##### Proportion of infants receiving open‐label medical treatment

Two Cochrane Reviews reported on the outcome of receipt of open‐label medical treatment for PDA. They included the following interventions.

###### Prophylactic ibuprofen

Prophylactic ibuprofen versus placebo: the review by Ohlsson 2020a showed that compared to placebo or no intervention, prophylactic ibuprofen reduced subsequent open‐label therapy for PDA (RR 0.17, 95% CI 0.11 to 0.26; 6 RCTs, 776 infants).

###### Chest shielding during phototherapy

Chest shielding during phototherapy versus control: the review by Bhola 2015 showed that compared to control, chest shielding during phototherapy reduced the subsequent need for open‐label treatment for PDA (RR 0.12, 95% CI 0.02 to 0.88; 1 RCT, 74 infants).

##### Proportion of infants requiring surgical ligation or transcatheter occlusion

Three Cochrane Reviews reported on the outcome invasive PDA closure by surgical ligation or transcatheter occlusion. They included the following interventions.

###### Prophylactic indomethacin

Prophylactic indomethacin versus placebo: the review by Fowlie 2010 showed that compared to control, prophylactic indomethacin reduced the need for invasive PDA closure (RR 0.51, 95% CI 0.37 to 0.71; 8 RCTs, 1791 infants).

###### Prophylactic ibuprofen

Prophylactic ibuprofen versus placebo: the review by Ohlsson 2020a showed that compared to placebo or no intervention, prophylactic ibuprofen reduced the need for invasive PDA closure (RR 0.46, 95% CI 0.22 to 0.96; 7 RCTs, 925 infants; moderate‐certainty evidence).

###### Chest shielding during phototherapy

Chest shielding during phototherapy versus control: the review by Bhola 2015 showed that there was no evidence of a difference between chest shielding during phototherapy and control for invasive PDA closure (RR 0.35, 95% CI 0.01 to 8.36; 1 RCT, 74 infants).

##### Chronic lung disease (CLD)

Five Cochrane Reviews reported on the outcome of CLD (all definitions included). They included the following interventions.

###### Prophylactic indomethacin

Prophylactic indomethacin versus placebo: the review by Fowlie 2010 showed that there was no evidence of a difference between prophylactic indomethacin and control for CLD, defined as oxygen requirement at 28 postnatal days (RR 1.08, 95% CI 0.92 to 1.26; 9 RCTs, 1022 infants), or CLD, defined as oxygen requirement at 36 weeks' postmenstrual age (RR 1.06, 95% CI 0.92 to 1.22; 1 RCT, 999 infants).

###### Prophylactic ibuprofen

Prophylactic ibuprofen versus placebo: the review by Ohlsson 2020a showed that there was no evidence of a difference between prophylactic ibuprofen and placebo or no intervention for CLD, defined as oxygen requirement at 28 postnatal days (RR 0.88, 95% CI 0.32 to 2.42; 1 RCT, 41 infants), CLD, defined as oxygen requirement at 36 weeks' postmenstrual age (RR 1.06, 95% CI 0.89 to 1.26; 5 RCTs, 817 infants), or CLD with an unspecified age at diagnosis (RR 0.94, 95% CI 0.51 to 1.72; 2 RCTs, 99 infants).

###### Prophylactic acetaminophen

Prophylactic acetaminophen versus placebo: the review by Jasani 2022 showed that there was no evidence of a difference between prophylactic acetaminophen and placebo or no intervention for CLD, defined as oxygen requirement at 28 postnatal days (RR 0.69, 95% CI 0.32 to 1.48; 1 RCT, 48 infants), or CLD, defined as oxygen requirement at 36 weeks' postmenstrual age (RR 0.36, 95% CI 0.02 to 8.45; 1 RCT, 48 infants).

###### Prophylactic surgical PDA ligation

Prophylactic surgical PDA ligation versus control (prophylactic cyclooxygenase inhibitor drugs only): the review by Mosalli 2008 showed that there was no evidence of a difference between prophylactic surgical PDA ligation and control for CLD, defined as oxygen requirement at 36 weeks' postmenstrual age (RR 1.07, 95% CI 0.68 to 1.69; 1 RCT, 48 infants).

###### Restriction of fluid intake

Restricted versus liberal fluid intake: the review by Bell 2014 showed that there was no evidence of a difference between restriction of predominantly IV fluids and liberal fluid intake for CLD (no definition specified for CLD; RR 0.85, 95% CI 0.63 to 1.14; 4 RCTs, 526 infants).

##### Intraventricular haemorrhage (IVH; grades I to IV)

Four Cochrane Reviews reported on the outcome of IVH (grades I to IV). They included the following interventions.

###### Prophylactic indomethacin

Prophylactic indomethacin versus placebo: the review by Fowlie 2010 showed that compared to control, prophylactic indomethacin reduced all grades of IVH (RR 0.88, 95% CI 0.80 to 0.98; 14 RCTs, 2532 infants).

###### Prophylactic ibuprofen

Prophylactic ibuprofen versus placebo: the review by Ohlsson 2020a showed that there was no evidence of a difference between prophylactic ibuprofen and placebo or no intervention for all grades of IVH (RR 0.96, 95% CI 0.78 to 1.17; 6 RCTs, 901 infants).

###### Chest shielding during phototherapy

Chest shielding during phototherapy versus control: the review by Bhola 2015 showed that there was no evidence of a difference between chest shielding during phototherapy and control for all grades of IVH (RR 0.53, 95% CI 0.10 to 2.71; 1 RCT, 74 infants).

###### Restriction of fluid intake

Restricted versus liberal fluid intake: the review by Bell 2014 showed that there was no evidence of a difference between restriction of predominantly IV fluids and liberal fluid intake with respect to all grades of IVH (RR 0.74, 95% CI 0.48 to 1.14; 3 RCTs, 356 infants).

##### Pulmonary haemorrhage

One Cochrane Review reported on the outcome pulmonary haemorrhage. It included the following intervention.

###### Prophylactic indomethacin

Prophylactic indomethacin versus placebo: the review by Fowlie 2010 showed that there was no evidence of a difference between prophylactic indomethacin and control for pulmonary haemorrhage (RR 0.84, 95% CI 0.66 to 1.07; 4 RCTs, 1591 infants).

##### Retinopathy of prematurity (ROP)

Five Cochrane Reviews reported on the outcome of ROP. They included the following interventions.

###### Prophylactic indomethacin

Prophylactic indomethacin versus placebo: the review by Fowlie 2010 showed that there was no evidence of a difference between prophylactic indomethacin and control for any stage of ROP (RR 1.02, 95% CI 0.92 to 1.12; 5 RCTs, 1571 infants), or for severe ROP (RR 1.75, 95% CI 0.92 to 3.34; 2 RCTs, 289 infants).

###### Prophylactic ibuprofen

Prophylactic ibuprofen versus placebo: the review by Ohlsson 2020a showed that there was no evidence of a difference between prophylactic ibuprofen and placebo or no intervention for ROP (RR 1.01, 95% CI 0.73 to 1.38; 5 RCTs, 369 infants).

###### Prophylactic acetaminophen

Prophylactic acetaminophen versus placebo: the review by Jasani 2022 showed that there was no evidence of a difference between prophylactic acetaminophen and placebo or no intervention for ROP (defined as any ROP that required treatment; RR 3.25, 95% CI 0.14 to 76.01; 1 RCT, 48 infants).

###### Prophylactic surgical PDA ligation

Prophylactic surgical PDA ligation versus control (prophylactic cyclooxygenase inhibitor drugs only): the review by Mosalli 2008 showed that there was no  difference between prophylactic surgical PDA ligation and control for any stage of ROP (RR 0.67, 95% CI 0.31 to 1.43; 1 RCT, 43 infants), or for severe ROP (RR 0.32, 95% CI 0.04 to 2.82; 1 RCT, 43 infants).

###### Chest shielding during phototherapy

Chest shielding during phototherapy versus control: the review by Bhola 2015 showed that there was no evidence of a difference between chest shielding during phototherapy and control for any stage of ROP (RR 0.53, 95% CI 0.10 to 2.71; 1 RCT, 74 infants).

##### Duration of hospitalisation (days)

Two Cochrane Reviews reported on duration of hospitalisation. They included the following interventions.

###### Prophylactic ibuprofen

Prophylactic ibuprofen versus placebo: the review by Ohlsson 2020a showed that there was no evidence of a difference between prophylactic ibuprofen and placebo or no intervention for duration of hospitalisation (mean difference (MD) 1.30 days, 95% CI ‐3.07 to 5.67; 6 RCTs, 447 infants).

###### Chest shielding during phototherapy

Chest shielding during phototherapy versus control: the review by Bhola 2015 showed that there was no evidence of a difference between chest shielding during phototherapy and control for duration of hospitalisation (MD ‐8.05 days, 95% CI ‐18.04 to 1.94; 2 RCTs, 128 infants).

##### Moderate/severe neurodevelopmental disability

One Cochrane Review reported on the outcome of moderate/severe neurodevelopmental disability. It included the following intervention.

###### Prophylactic indomethacin

Prophylactic indomethacin versus placebo: the review by Fowlie 2010 showed that there was no evidence of a difference between prophylactic indomethacin and control for moderate/severe neurodevelopmental disability (RR 0.96, 95% CI 0.79 to 1.17; 3 RCTs, 1286 infants).

##### All‐cause mortality

Six Cochrane Reviews reported on mortality (all time points included). They included the following interventions.

###### Prophylactic indomethacin

Prophylactic indomethacin versus placebo: the review by Fowlie 2010 showed that there was no evidence of a difference between prophylactic indomethacin and control for mortality during the hospital stay (RR 0.82, 95% CI 0.65 to 1.03; 17 RCTs, 1567 infants), or mortality at the latest follow‐up (RR 0.96, 95% CI 0.81 to 1.12; 18 RCTs, 2769 infants).

###### Prophylactic ibuprofen

Prophylactic ibuprofen versus placebo: the review by Ohlsson 2020a showed that there was no evidence of a difference between prophylactic ibuprofen and placebo or no intervention for neonatal mortality (RR 0.93, 95% CI 0.50 to 1.74; 6 RCTs, 342 infants), mortality during the hospital stay (RR 0.90, 95% CI 0.62 to 1.30; 4 RCTs, 700 infants), or mortality before 36 weeks' postmenstrual age (RR 0.96, 95% CI 0.56 to 1.66; 1 RCT, 131 infants).

###### Prophylactic acetaminophen

Prophylactic acetaminophen: the review by Jasani 2022 showed that there was no evidence of a difference between prophylactic acetaminophen and control for mortality during the hospital stay (RR 0.59, 95% CI 0.24 to 1.44; 3 RCTs, 240 infants; low‐certainty evidence).

###### Prophylactic surgical PDA ligation

Prophylactic surgical PDA ligation versus control (prophylactic cyclooxygenase inhibitor drugs only): the review by Mosalli 2008 showed that there was no evidence of a difference between prophylactic surgical PDA ligation and control for neonatal mortality (< 28 days of life; RR 0.88, 95% CI 0.53 to 1.45; 1 RCT, 84 infants), or for mortality at one year (RR 1.06, 95% CI 0.75 to 1.49; 1 RCT, 84 infants).

###### Chest shielding during phototherapy

Chest shielding during phototherapy versus control: the review by Bhola 2015 showed that there was no evidence of a difference between chest shielding during phototherapy and control for mortality during the hospital stay (RR 1.68, 95% CI 0.75 to 3.78; 2 RCTs, 128 infants), or for neonatal mortality (< 28 days of age; RR 1.06, 95% CI 0.16 to 7.10; 1 RCT, 74 infants).

###### Restriction of fluid intake

Restricted versus liberal fluid intake: the review by Bell 2014 showed that there was no evidence of a difference between restriction of predominantly IV fluids and liberal fluid intake for mortality before discharge (RR 0.81, 95% CI 0.54 to 1.23; 5 RCTs, 582 infants).

##### Necrotising enterocolitis (NEC)

Five Cochrane Reviews reported on NEC. They included the following interventions.

###### Prophylactic indomethacin

Prophylactic indomethacin versus placebo: the review by Fowlie 2010 showed that there was no evidence of a difference between prophylactic indomethacin and control for NEC (RR 1.09, 95% CI 0.82 to 1.46; 12 RCTs, 2401 infants).

###### Prophylactic ibuprofen

Prophylactic ibuprofen versus placebo: the review by Ohlsson 2020a showed that there was no evidence of a difference between prophylactic ibuprofen and placebo or no intervention for NEC (RR 0.96, 95% CI 0.61 to 1.50; 9 RCTs, 1028 infants; moderate‐certainty evidence).

###### Prophylactic acetaminophen

Prophylactic acetaminophen versus placebo: the review by Jasani 2022 showed that there was no evidence of a difference between prophylactic acetaminophen and placebo or no intervention for NEC (RR 0.36, 95% CI 0.02 to 8.45; 1 RCT, 48 infants).

###### Prophylactic surgical PDA ligation

Prophylactic surgical PDA ligation versus control (prophylactic cyclooxygenase inhibitor drugs only): the review by Mosalli 2008 showed that compared to control prophylactic cyclooxygenase inhibitor drugs, prophylactic surgical PDA ligation reduced NEC (RR 0.25, 95% CI 0.08 to 0.83; 1 RCT, 84 infants).

###### Restriction of fluid intake

Restricted versus liberal fluid intake: the review by Bell 2014 showed that compared to liberal fluid intake, restriction of predominantly IV fluids reduced NEC (RR 0.43, 95% CI 0.21 to 0.87; 4 RCTs, 526 infants).

##### Gastrointestinal perforation

Two Cochrane Reviews reported on gastrointestinal perforation. They included the following interventions.

###### Prophylactic indomethacin

Prophylactic indomethacin versus placebo: the review by Fowlie 2010 showed that there was no evidence of a difference between prophylactic indomethacin and control for gastrointestinal perforation (RR 1.13, 95% CI 0.71 to 1.79; 1 RCT, 1202 infants).

###### Prophylactic ibuprofen

Prophylactic ibuprofen versus placebo: the review by Ohlsson 2020a showed that there was no evidence of a difference between prophylactic ibuprofen and placebo or no intervention for gastrointestinal perforation (RR 4.88, 95% CI 0.87 to 27.36; 2 RCTs, 167 infants).

##### Gastrointestinal bleeding

One Cochrane Review reported on gastrointestinal bleeding. It included the following intervention.

###### Prophylactic ibuprofen

Prophylactic ibuprofen versus placebo: the review by Ohlsson 2020a showed that compared to placebo or no intervention, gastrointestinal bleeding increased with prophylactic ibuprofen (RR 2.05, 95% CI 1.19 to 3.51; 5 RCTs, 282 infants; low‐certainty evidence).

##### Oliguria

Three Cochrane Reviews reported on the outcome of oliguria. They included the following interventions.

###### Prophylactic indomethacin

Prophylactic indomethacin versus placebo: the review by Fowlie 2010 showed that compared to control, prophylactic indomethacin increased oliguria (RR 1.90, 95% CI 1.45 to 2.47; 8 RCTs, 2115 infants).

###### Prophylactic ibuprofen

Prophylactic ibuprofen versus placebo: the review by Ohlsson 2020a showed that compared to placebo or no intervention, prophylactic ibuprofen increased oliguria (RR 1.45, 95% CI 1.04 to 2.02; 4 RCTs, 747 infants; high‐certainty evidence).

###### Prophylactic acetaminophen

Prophylactic acetaminophen versus placebo: the review by Jasani 2022 showed that there was no evidence of a difference between prophylactic acetaminophen and placebo or no intervention on oliguria (RR 0.78, 95% CI 0.29 to 2.11; 1 RCT, 48 infants).

##### Serum/plasma levels of creatinine after treatment

One Cochrane Review reported on this outcome. It included the following intervention.

###### Prophylactic ibuprofen

Prophylactic ibuprofen versus placebo: the review by Ohlsson 2020a showed that there was no evidence of a difference between prophylactic ibuprofen and placebo or no intervention on serum creatinine levels post‐treatment (weighted mean difference (WMD)** 0.09 mg/dL, 95% CI 0.05 to 0.13; 6 RCTs, 800 infants; low‐certainty evidence).

** *Please note that Cochrane now uses mean difference.*

##### Increase in serum/plasma levels of creatinine after treatment

Two Cochrane Reviews reported on this outcome. They included the following interventions.

###### Prophylactic indomethacin

Prophylactic indomethacin versus placebo. The review by Fowlie 2010 showed that there was no evidence of a difference between prophylactic indomethacin and control on serum/plasma levels of creatinine after treatment (RR 1.09, 95% CI 0.47 to 1.79; 4 RCTs, 618 infants).

###### Prophylactic ibuprofen

Prophylactic ibuprofen versus placebo: the review by Ohlsson 2020a showed that compared to placebo or no intervention, prophylactic ibuprofen increased serum/plasma levels of creatinine after treatment (RR 3.70, 95% CI 1.05 to 12.98; 2 RCTs, 285 infants).

##### Serum/plasma levels of bilirubin after treatment

One Cochrane Review reported on this outcome. It included the following intervention.

###### Prophylactic acetaminophen

Prophylactic acetaminophen versus placebo: the review by Jasani 2022 showed that there was no evidence of a difference between prophylactic acetaminophen and placebo or no intervention on serum/plasma levels of bilirubin after treatment (MD 1 µmol/L, 95% CI ‐10.35 to 12.35; 1 RCT, 48 infants).

#### Interventions (pharmacological or surgical) for management of asymptomatic PDA in preterm infants

Only one Cochrane Review, which compared indomethacin for asymptomatic PDA versus placebo addressed this objective (Cooke 2003). It included the following outcomes (Table 7).

**7:** Interventions (pharmacological or surgical) for management of asymptomatic PDA in preterm infants

**Intervention**	**Indomethacin (vs placebo)**
**Outcome**	
**Symptomatic PDA**	0.36, 95% CI 0.19 to 0.68; 3 RCTs, N = 97
**Need for invasive PDA closure** (surgical ligation or transcatheter occlusion)	0.45, 95% CI 0.17 to 1.21; 2 RCTs, N = 73
**Chronic lung disease**	0.91, 95% CI 0.62 to 1.35; 2 RCTs, N = 45
**Retinopathy of prematurity**	0.68, 95% CI 0.26 to 1.78; 2 RCTs, N = 55
**Duration of hospitalisation**	MD 11 days, 95% CI ‐45.21 to 23.21; 1 RCT, N = 26
**Mortality**	1.32, 95% CI 0.45 to 3.86; 2 RCTs, N = 73
**Necrotising enterocolitis**	0.41, 95% CI 0.05 to 3.68; 1 RCT, N = 47

**CI**: confidence interval; **MD**: mean difference; **PDA**: patent ductus arteriosus; **RCT**: randomised controlled trials Data presented as risk ratio (RR), unless otherwise specified.

##### Symptomatic PDA

Compared to placebo, treatment of asymptomatic PDA with indomethacin reduced the development of symptomatic PDA post‐treatment RR 0.36, 95% CI 0.19 to 0.68; (3 RCTs, 97 infants).

##### Proportion of infants requiring invasive PDA closure (surgical ligation or transcatheter occlusion)

There was no evidence of a difference between the treatment of asymptomatic PDA with indomethacin and placebo on the need for invasive PDA closure (RR 0.45, 95% CI 0.17 to 1.21; 2 RCTs, 73 infants).

##### Chronic lung disease (CLD)

There was no evidence of a difference between treatment of asymptomatic PDA with indomethacin and placebo for CLD (RR 0.91, 95% CI 0.62 to 1.35; 2 RCTs, 45 infants).

##### Retinopathy of prematurity (ROP)

There was no evidence of a difference between treatment of asymptomatic PDA with indomethacin and placebo for any stage of ROP (RR 0.68, 95% CI 0.26 to 1.78; 2 RCTs, 55 infants).

##### Duration of hospitalisation (days)

There was no evidence of a difference between treatment of asymptomatic PDA with indomethacin and placebo on the duration of hospitalisation (MD 11 days, 95% CI ‐45.21 to 23.21; 1 RCT, 26 infants).

##### Mortality

There was no evidence of a difference between treatment of asymptomatic PDA with indomethacin and placebo for mortality (RR 1.32, 95% CI 0.45 to 3.86; 2 RCTs, 73 infants).

##### Necrotising enterocolitis (NEC)

There was no evidence of a difference between treatment of asymptomatic PDA with indomethacin and placebo on NEC (RR 0.41, 95% CI 0.05 to 3.68; 1 RCT, 47 infants).

#### Interventions (pharmacological or surgical) for management of symptomatic (haemodynamically significant) PDA in preterm infants

##### Failure of PDA closure after completion of allocated treatment

Eight Cochrane Reviews reported on the outcome of failure of PDA closure. They included the following interventions (Table 8).

**8:** Interventions for symptomatic PDA: failure of PDA closure after completion of allocated treatment

** **		**Comparison**		
		**Indomethacin**	**Ibuprofen**	**Placebo/no treatment**
**Therapy**	**Indomethacin**	Continuous vs intermittent bolus* PDA closure day 2: 1.57, 95% CI 0.54 to 4.60; 2 RCTs, N = 48 PDA closure day 5: 2.77, 95% CI 0.33 to 23.14; (1 RCT, N = 25	‐	0.30, 95% CI 0.23 to 0.28; 10 RCTs, N = 654; high‐certainty evidence
		Prolonged vs short course* 0.82, 95% CI 0.51 to 1.33; 4 RCTs, N = 361		
	**Ibuprofen**	Ibuprofen IV/PO vs indomethacin IV/PO* 1.07, 95% CI 0.92 to 1.24; 24 RCTs, N = 1590; moderate‐certainty evidence	Ibuprofen PO vs ibuprofen IV* 0.38, 95% CI 0.26 to 0.56; 5 RCTs, N = 406; moderate‐certainty evidence	*Ibuprofen IV* 0.62, 95% CI 0.44 to0.86; 2 RCTs, N = 206; moderate‐certainty evidence
			Ibuprofen high‐dose vs standard‐dose* 0.37, 95% CI 0.22 to 0.61; 3 RCTs, N = 190; moderate‐certainty evidence	
		Ibuprofen PO vs indomethacin IV/PO* 0.96, 95% CI 0.73 to 1.27;8 RCTs, N = 272; low‐certainty evidence	Ibuprofen IV: echocardiography‐guided vs standard administration* 1.31, 95% CI 0.44 to 3.91; 1 RCT, N = 49	*Ibuprofen PO* 0.26, 95% CI 0.11 to 0.62; 1 RCT, N = 64
			Ibuprofen IV: continuous infusion vs Intermittent bolus* 1.18, 95% CI 0.88 to 1.5; 1 RCT, N = 111	
			Ibuprofen PR vs PO* 0.83, 95% CI 0.28 to 2.4; RCT, N = 72	
	**Acetaminophen**	1.02, 95% CI 0.78 to 1.33; 4 RCTs, N = 380; low‐certainty evidence	1.02, 95% CI 0.88 to 1.18; 18 RCTs, N = 1535; moderate‐certainty evidence	*Early treatment(< day 7)* 0.35, 95% CI 0.23 to 0.53; 2 RCTs, N = 127; low‐certainty evidence
				*Late treatment (≥ day 14)* 0.85, 95% CI 0.72 to 1.01; 1 RCT, N = 55; low‐certainty evidence
	**Ibuprofen + Acetaminophen**	‐	0.77, 95% CI 0.43 to 1.36; 2 RCTs, N = 111; low‐certainty evidence	‐
	**Surgical Ligation**	0.04, 95% CI 0.01 to 0.27; 1 RCT, N = 154	‐	‐
	**Furosemide with indomethacin**	1.25, 95% CI 0.62 to 2.52; 3 RCTs, N = 70	‐	‐
	**Dopamine with indomethacin**	1.11, 95% CI 0.56 to 2.19; 3 RCTs, N = 74	‐	‐

**CI**: confidence interval; **IV**: intravenous; **PO**: per os; **PDA**: patent ductus arteriosus; **PR**: per rectum; **RCT**: randomised controlled trials; **vs**: versus Reference is the listed comparison, therapy unless otherwise indicated by * Differences between intervention and comparison provided as risk ratio (RR), unless otherwise specified; certainty of evidence added when available from review.

###### Indomethacin

Indomethacin versus placebo or no treatment: the review by Evans 2021 showed that compared to placebo or no treatment, indomethacin reduced failure of PDA closure post‐treatment (RR 0.30, 95% CI 0.23 to 0.38; 10 RCTs, 654 infants; high‐certainty evidence).

Prolonged versus short course of indomethacin: the review by Herrera 2007 showed that there was no evidence of a difference between a prolonged and short course of indomethacin on failure of PDA closure post‐treatment (RR 0.82, 95% CI 0.51 to 1.33; 4 RCTs, 361 infants).

Continuous infusion versus intermittent bolus of indomethacin: the review by Görk 2008 showed that there was no evidence of a difference between continuous infusion and intermittent bolus of indomethacin on failure of PDA closure post‐treatment by day two (RR 1.57, 95% CI 0.54 to 4.60; 2 RCTs, 48 infants), or by day five (RR 2.77, 95% CI 0.33 to 23.14; 1 RCT, 25 infants).

###### Ibuprofen

Intravenous ibuprofen versus placebo or no treatment: the review by Ohlsson 2020b showed that compared to placebo or no treatment, IV ibuprofen reduced failure of PDA closure post‐treatment (RR 0.62, 95% CI 0.44 to 0.86; 2 RCTs, 206 infants; moderate‐certainty evidence).

Oral ibuprofen versus placebo or no treatment: the review by Ohlsson 2020b showed that compared to placebo or no treatment, oral ibuprofen reduced failure of PDA closure post‐treatment (RR 0.26, 95% CI 0.11 to 0.62; 1 RCT, 64 infants).

Ibuprofen (IV or oral) versus indomethacin (IV or oral): the review by Ohlsson 2020b showed that there was no evidence of a difference between ibuprofen and indomethacin on failure of PDA closure post‐treatment (RR 1.07, 95% CI 0.92 to 1.24; 24 RCTs, 1590 infants; moderate‐certainty evidence).

Oral ibuprofen versus indomethacin (IV or oral): the review by Ohlsson 2020b showed that there was no evidence of a difference between oral ibuprofen and indomethacin on failure of PDA closure post‐treatment (RR 0.96, 95% CI 0.73 to 1.27; 8 RCTs, 272 infants; low‐certainty evidence).

Oral ibuprofen versus IV ibuprofen: the review by Ohlsson 2020b showed that compared to IV ibuprofen, oral ibuprofen reduced failure of PDA closure post‐treatment (RR 0.38, 95% CI 0.26 to 0.56; 5 RCTs, 406 infants; moderate‐certainty evidence).

High‐dose ibuprofen versus standard dose ibuprofen: the review by Ohlsson 2020b showed that compared to standard dose ibuprofen, high dose ibuprofen reduced failure of PDA closure post‐treatment (RR 0.37, 95% CI 0.22 to 0.61; 3 RCTs, 190 infants; moderate‐certainty evidence).

Echocardiogram‐guided versus standard IV ibuprofen: the review by Ohlsson 2020b showed that there was no evidence of a difference between echocardiogram‐guided and standard IV ibuprofen on failure of PDA closure post‐treatment (RR 1.31, 95% CI 0.44 to 3.91; 1 RCT, 49 infants).

Continuous infusion of ibuprofen versus intermittent boluses of ibuprofen: the review by Ohlsson 2020b showed that there was no evidence of a difference between continuous infusion of ibuprofen and intermittent boluses of ibuprofen on failure of PDA closure post‐treatment (RR 1.18, 95% CI 0.88 to 1.5; 1 RCT, 111 infants).

Rectal versus oral ibuprofen: the review by Ohlsson 2020b showed that there was no evidence of a difference between rectal and oral ibuprofen on failure of PDA closure post‐treatment (RR 0.83, 95% CI 0.28 to 2.4; 1 RCT, 72 infants).

###### Acetaminophen

Acetaminophen versus ibuprofen: the review by Jasani 2022 showed that there was no evidence of a difference between acetaminophen and ibuprofen on failure of PDA closure post‐treatment (RR 1.02, 95% CI 0.88 to 1.18; 18 RCTs, 1535 infants; moderate‐certainty evidence).

Acetaminophen versus indomethacin: the review by Jasani 2022 showed that there was no evidence of a difference between acetaminophen and indomethacin on failure of PDA closure post‐treatment (RR 1.02, 95% CI 0.78 to 1.33; 4 RCTs, 380 infants; low‐certainty evidence).

Early acetaminophen versus placebo: the review by Jasani 2022 showed that compared to placebo, early acetaminophen reduced failure of PDA closure post‐treatment (RR 0.35, 95% CI 0.23 to 0.53; 2 RCTs, 127 infants; low‐certainty evidence).

Late acetaminophen versus placebo: the review by Jasani 2022 showed that there was no evidence of a difference between late acetaminophen and placebo on failure of PDA closure post‐treatment (RR 0.85, 95% CI 0.72 to 1.01; 1 RCT, 55 infants; low‐certainty evidence).

Acetaminophen and ibuprofen combination therapy versus ibuprofen alone: the review by Jasani 2022 showed that there was no evidence of a difference between acetaminophen and ibuprofen combination therapy and ibuprofen alone on failure of PDA closure post‐treatment (RR 0.77, 95% CI 0.43 to 1.36; 2 RCTs, 111 infants; low‐certainty evidence).

###### Surgical ligation

Surgical PDA ligation versus medical treatment with indomethacin: the review by Malviya 2013 showed that compared to medical therapy, surgical ligation reduced failure of PDA closure post‐treatment (RR 0.04, 95% CI 0.01 to 0.27; 1 RCT, 154 infants).

###### Adjunct therapies

Furosemide versus control: the review by Brion 2001 showed that there was no evidence of a difference between the combination of furosemide and indomethacin versus indomethacin alone on failure of PDA closure post‐treatment (RR 1.25, 95% CI 0.62 to 2.52; 3 RCTs, 70 infants).

Dopamine versus control: the review by Barrington 2002 showed that there was no evidence of a difference between the combination of dopamine and indomethacin versus indomethacin alone on failure of PDA closure post‐treatment (RR 1.11, 95% CI 0.56 to 2.19; 3 RCTs, 74 infants).

##### Death or moderate/severe neurodevelopmental disability

No reviews reported on the combined outcome of death or moderate/severe neurodevelopmental disability.

##### Proportion of infants receiving open‐label medical treatment

Four Cochrane Reviews reported on the outcome of receipt of open‐label treatment. They included the following interventions (Table 9).

**9:** Interventions for symptomatic PDA: proportion of infants receiving open‐label medical treatment

		**Comparison**	
		**Indomethacin**	**Placebo/no treatment**
**Therapy**	**Indomethacin**	Prolonged vs short course* 0.95, 95% CI 0.67 to 1.34; 5 RCTs, N = 431	*Indomethacin vs placebo/no treatment* 0.35, 95% CI 0.23 to 0.54; 6 RCTs, N = 211
			Very early treatment (≤ day 3) vs expectant management* 0.52, 95% CI 0.26 to 1.02; 1 RCT, N = 92
			Early treatment (≤ day 7) vs expectant management* 0.33, 95% CI 0.01 to 7.91; 1 RCT, N = 127
	**Ibuprofen**	‐	Ibuprofen IV vs placebo/no treatment* 1.20, 95% CI 0.76 to 1.90; 7 RCTs, N = 241
			Very early treatment (≤ day 3) vs expectant management* 1.06, 95% CI 0.07 to 16.26; 1 RCT, N = 72
			Early treatment (≤ day 7) vs expectant management* 0.66, 95% CI 0.27 to 1.60; 1 RCT, N = 105

**CI**: confidence interval; **IV**: intravenous; **PDA**: patent ductus arteriosus; **RCT**: randomised controlled trials; **vs:** versus Reference is the listed comparison, therapy unless otherwise indicated by * Differences between intervention and comparison provided as risk ratio (RR), unless otherwise specified.

###### Indomethacin

Indomethacin versus placebo or no treatment: the review by Evans 2021 showed that compared to placebo or no treatment, indomethacin reduced the need for open‐label treatment (RR 0.35, 95% CI 0.23 to 0.54; 6 RCTs, 211 infants).

Prolonged versus short course of indomethacin: the review by Herrera 2007 showed that there was no evidence of a difference between prolonged and short course of indomethacin on the need for open‐label treatment (RR 0.95, 95% CI 0.67 to 1.34; 5 RCTs, 431 infants).

Early treatment (initiated within seven days) versus expectant management: the review by Mitra 2020a showed that there was no evidence of a difference between early treatment with indomethacin and expectant management on the need for open‐label treatment (RR 0.33, 95% CI 0.01 to 7.91; 1 RCT, 127 infants).

Very early treatment (initiated within three days) versus expectant management: the review by Mitra 2020a showed that there was no evidence of a difference between very early treatment with indomethacin and expectant management on the need for open‐label treatment (RR 0.52, 95% CI 0.26 to 1.02; 1 RCT, 92 infants).

###### Ibuprofen

Intravenous ibuprofen versus placebo or no treatment: the review by Ohlsson 2020b showed that there was no evidence of a difference between IV ibuprofen and placebo or no treatment on the need for open‐label treatment (RR 1.20, 95% CI 0.76 to 1.90; 7 RCTs, 241 infants).

Early treatment (initiated within seven days) versus expectant management: the review by Mitra 2020a showed that there was no evidence of a difference between early treatment with ibuprofen and expectant management on the need for open‐label treatment (RR 0.66, 95% CI 0.27 to 1.60; 1 RCT, 105 infants).

Very early treatment (initiated within three days) versus expectant management: the review by Mitra 2020a showed that there was no evidence of a difference between very early treatment with ibuprofen and expectant management on the need for open‐label treatment (RR 1.06, 95% CI 0.07 to 16.26; 1 RCT, 72 infants).

##### Proportion of infants requiring invasive PDA closure (surgical ligation or transcatheter occlusion)

Five reviews reported on the outcome of invasive PDA closure. They included the following interventions (Table 10).

**10:** Interventions for symptomatic PDA: proportion of infants requiring PDA closure (surgical ligation or transcatheter occlusion)

** **		**Comparison**		
		**Indomethacin**	**Ibuprofen**	**Placebo/notreatment**
**Therapy**	**Indomethacin**	Prolonged vs short course* 0.86, 95% CI 0.49 to 1.51; 4 RCTs, N = 310	‐	Indomethacin vs placebo/no treatment* 0.66, 95% CI 0.33 to 1.29; 6 RCTs, N = 275; moderate‐certainty evidence
				Very early treatment (≤ day 3) vs expectant management* 0.54, 95% CI 0.07 to 3.93; 3 RCTs, N = 161
				Early treatment (≤ day 7) vs expectant management* 0.74, 95% CI 0.17 to 3.17; 1 RCT, N =127
	**Ibuprofen**	Ibuprofen IV/PO vs indomethacinIV/PO* 1.06, 95% CI 0.81 to 1.39; 16 RCTs, N = 1275; moderate‐certainty evidence	Ibuprofen PO vs Ibuprofen IV* 0.41, 95% CI 0.14 to 1.21; 5 RCTs, N = 406; moderate‐certainty evidence	Ibuprofen IV vs placebo/no treatment* 1.89, 95% CI 0.91to 3.93; 1 RCT, N = 134
			Ibuprofen high‐dose vs standard‐dose* 1.00, 95% CI 0.15 to 6.71; 1 RCT, N = 70	
		Ibuprofen PO vs indomethacin IV/PO* 0.93, 95% CI 0.50 to 1.74; 4 RCTs, N = 174; low‐certainty evidence	Ibuprofen IV: continuous infusion vs intermittent bolus* 0.28, 95% CI 0.08 to 0.94; 1 RCT, N = 111	Very early treatment (≤ day 3) vs expectant management* 1.00, 95% CI 0.36 to 2.75; 1 RCT, N = 60
			Ibuprofen PR vs PO* 1.00, 95% CI 0.15 to 6.72; 1 RCT, N = 72	Early treatment (≤ day 7) vs expectant management* 1.14, 95% CI 0.66 to 1.96; 3 RCTs, N = 305
	**Acetaminophen**	1.31, 95% CI (0.72 to 2.38); (2 RCTs, N =237)	0.61, 95% CI 0.34 to 1.08; 6 RCTs, N = 603	*Late treatment (≥ day 14)* 3.11, 95% CI 0.13 to 73.11; 1 RCT, N = 55

**CI**: confidence interval; **IV**: intravenous; **PO:** per os; **PDA**: patent ductus arteriosus; **PR**: per rectum; **RCT**: randomised controlled trials; **vs:** versus Reference is the listed comparison, therapy unless otherwise indicated by * Differences between intervention and comparison provided as risk ratio (RR), unless otherwise specified: certainty of evidence added when available from review

###### Indomethacin

Indomethacin versus placebo or no treatment: the review by Evans 2021 showed that there was no evidence of a difference between indomethacin and placebo or no treatment on the need for invasive PDA closure (RR 0.66, 95% CI 0.33 to 1.29; 6 RCTs, 275 infants; moderate‐certainty evidence).

Prolonged versus short course of indomethacin: the review by Herrera 2007 showed that there was no evidence of a difference between prolonged and short course of indomethacin on the need for invasive PDA closure (RR 0.86, 95% CI 0.49 to 1.51; 4 RCTs, 310 infants).

Early treatment (initiated within seven days) versus expectant management: the review by Mitra 2020a showed that there was no evidence of a difference between early treatment with indomethacin and expectant management on the need for invasive PDA closure (RR 0.74, 95% CI 0.17 to 3.17; 1 RCT, 127 infants).

Very early treatment (initiated within three days) versus expectant management: the review by Mitra 2020a showed that there was no evidence of a difference between very early treatment with indomethacin and expectant management on the need for invasive PDA closure (RR 0.54, 95% CI 0.07 to 3.93; 3 RCTs, 161 infants).

###### Ibuprofen

Intravenous ibuprofen versus placebo or no treatment: the review by Ohlsson 2020b showed that there was no evidence of a difference between IV ibuprofen and placebo or no treatment on the need for invasive PDA closure (RR 1.89, 95% CI 0.91 to 3.93; 1 RCT, 134 infants).

Ibuprofen versus indomethacin: the review by Ohlsson 2020b showed that there was no evidence of a difference between ibuprofen and indomethacin on the need for invasive PDA closure (RR 1.06, 95% CI 0.81 to 1.39; 16 RCTs, 1275 infants; moderate‐certainty evidence).

Oral ibuprofen versus indomethacin: the review by Ohlsson 2020b showed that there was no evidence of a difference between oral ibuprofen and indomethacin on the need for invasive PDA closure (RR 0.93, 95% CI 0.50 to 1.74; 4 RCTs, 174 infants; low‐certainty evidence).

Oral ibuprofen versus IV ibuprofen: the review by Ohlsson 2020b showed that there was no evidence of a difference between oral ibuprofen and IV ibuprofen on the need for invasive PDA closure (RR 0.41, 95% CI 0.14 to 1.21; 5 RCTs, 406 infants; moderate‐certainty evidence).

High‐dose ibuprofen versus standard‐dose ibuprofen: the review by Ohlsson 2020b showed that there was no evidence of a difference between high‐dose ibuprofen and standard‐dose ibuprofen on the need for invasive PDA closure (RR 1.00, 95% CI 0.15 to 6.71; 1 RCT, 70 infants).

Early treatment (initiated within seven days) versus expectant management: the review by Mitra 2020a showed that there was no evidence of a difference between early treatment with ibuprofen and expectant management on the need for invasive PDA closure (RR 1.14, 95% CI 0.66 to 1.96; 3 RCTs, 305 infants).

Very early treatment (initiated within three days) versus expectant management: the review by Mitra 2020a showed that there was no evidence of a difference between very early treatment with ibuprofen and expectant management on the need for invasive PDA closure (RR 1.00, 95% CI 0.36 to 2.75; 1 RCT, 60 infants).

Continuous infusion versus intermittent bolus of ibuprofen: the review by Ohlsson 2020b showed that compared to intermittent bolus of ibuprofen, continuous infusion of ibuprofen reduced the need for invasive PDA closure (RR 0.28, 95% CI 0.08 to 0.94; 1 RCT, 111 infants).

Rectal versus oral ibuprofen: the review by Ohlsson 2020b showed that there was no evidence of a difference between rectal ibuprofen and oral ibuprofen on the need for invasive PDA closure (RR 1.00, 95% CI 0.15 to 6.72; 1 RCT, 72 infants).

###### Acetaminophen

Acetaminophen versus ibuprofen: the review by Jasani 2022 showed that there was no evidence of a difference between acetaminophen and ibuprofen on the need for invasive PDA closure (RR 0.61, 95% CI 0.34 to 1.08; 6 RCTs, 603 infants).

Acetaminophen versus indomethacin: the review by Jasani 2022 showed that there was no evidence of a difference between acetaminophen and indomethacin on the need for invasive PDA closure (RR 1.31, 95% CI 0.72 to 2.38; 2 RCTs, 237 infants).

Late acetaminophen (initiated on day 14 or later) versus placebo: the review by Jasani 2022 showed that there was no evidence of a difference between late acetaminophen and placebo on the need for invasive PDA closure (RR 3.11, 95% CI 0.13 to 73.11; 1 RCT, 55 infants).

##### Proportion of infants receiving open‐label medical or surgical treatment in the placebo or no treatment group

No reviews reported on this outcome.

##### Chronic lung disease

Six reviews reported on the outcome of CLD (all definitions included). They include the following interventions (Table 11).

**11:** Interventions for symptomatic PDA: chronic lung disease

		**Comparison**		
		**Indomethacin**	**Ibuprofen**	**Placebo/no treatment**
**Therapy**	**Indomethacin**	Prolonged vs short course* 1.35, 95% CI 0.78 to 2.36; 2 RCTs, N = 201	‐	Indomethacin vs placebo/no treatment* Supplemental oxygen at 28 days of age 1.45, 95% CI 0.60 to 3.51; 1 RCT, N = 55 *Supplemental oxygen at 36 weeks' PMA* 0.80, 95% CI 0.41 to 1.55; 1 RCT, N = 92; low‐certainty evidence
				Very early treatment (≤ day 3) vs expectant management* 1.06, 95% CI 0.61 to 1.83; 4 RCTs, N = 188
				Early treatment (≤ day 7) vs expectant management* 0.84, 95% CI 0.52 to 1.37; 2 RCT, N = 168
	**Ibuprofen**	Ibuprofen IV/PO vs indomethacin IV/PO* Supplemental oxygen at 36 weeks' PMA 1.12, 95% CI 0.77 to 1.61; 3 RCTs, N = 357 *Supplemental oxygen at 28 days of age* 1.20, 95% CI 0.93 to 1.55; 5 RCTs, N = 292	Ibuprofen PO vs Ibuprofen IV* 0.82, 95% CI 0.56 to 1.20; 3 RCTs, N = 236	Ibuprofen IV vs placebo/no treatment* Supplemental oxygen at 36 weeks' PMA 0.99, 95% CI 0.88 to 1.11; 1 RCT, N = 98 *Supplemental oxygen at 28 days of age* 1.09, 95% CI 0.95 to 1.26; 1 RCT, N = 130
			Ibuprofen high‐dose vs standard‐dose* 1.60, 95% CI 0.85 to 3.02; 1 RCT, N = 70	
		Ibuprofen PO vs indomethacin IV/PO* Supplemental oxygen at 28 days of age RD 0.07, 95% CI ‐0.42 to 0.29; 1 RCT, N = 30 *Chronic lung disease (no definition specified)* ‐0.00, 95% CI ‐0.44 to 0.44; 1 RCT, N = 18	Ibuprofen IV: echocardiography‐guided vs standard administration* 1.35, 95% CI 0.53 to 3.44; 1 RCT, N = 49	Very early treatment (≤ day 3) vs expectant management* 0.54, 95% CI 0.35 to 0.83; 2 RCTs, N = 124
			Ibuprofen IV: continuous infusion vs intermittent bolus* 1.1, 95% CI 0.55 to 2.2; 1 RCT, N = 111	Early treatment (≤ day 7) vs expectant management* 0.97, 95% CI 0.56 to 1.29; 2 RCTs, N = 171
	**Acetaminophen**	1.16, 95% CI 0.77 to 1.75; 2 RCTs, N = 94	*Supplemental oxygen at 36 weeks' PMA* 0.79, 95% CI 0.45 to 1.38; 2 RCTs, N = 141 *Moderate/severe chronic lung disease* 0.80, 95% CI 0.22 to 2.87; 1 RCT, N = 160 *Severe chronic lung disease* 0.62, 95% CI 0.32 to 1.23; 1 RCT, N = 90	*Late treatment (≥ day 14)* 1.04, 95% CI 0.07 to 15.76; 1 RCT, N = 55
	**Ibuprofen + Acetaminophen**	‐	0.80, 95% CI 0.28 to 2.27; 1 RCT, N = 24	‐
	**Surgical ligation**	1.28, 95% CI 0.83 to 1.98; 1 RCT, N = 154	‐	‐

**CI**: confidence interval; **IV**: intravenous; **PO:** per os; **PDA**: patent ductus arteriosus; **PMA:** post‐menstrual age; **RCT**: randomised controlled trials; **RD**: risk difference; **vs:** versus Reference is the listed comparison therapy, unless otherwise indicated by * Differences between intervention and comparison provided as risk ratio (RR), unless otherwise specified; certainty of evidence added when available from review Outcome of chronic lung disease refers to need for supplemental oxygen at 36 weeks' post‐menstrual age, unless otherwise specified

###### Indomethacin

Indomethacin versus placebo or no treatment: the review by Evans 2021 showed that there was no evidence of a difference between indomethacin and placebo or no treatment for CLD defined as the need for supplemental oxygen at 36 weeks' postmenstrual age (RR 0.80, 95% CI 0.41 to 1.55; 1 RCT, 92 infants; low‐certainty evidence), or for CLD defined as the need for supplemental oxygen at 28 days of age (RR 1.45, 95% CI 0.60 to 3.51; 1 RCT, 55 infants).

Prolonged versus short course of indomethacin: the review by Herrera 2007 showed that there was no evidence of a difference between prolonged and short course of indomethacin for CLD (RR 1.35, 95% CI 0.78 to 2.36; 2 RCTs, 201 infants).

Early treatment (initiated within seven days) versus expectant management: the review by Mitra 2020a showed that there was no evidence of a difference between early treatment with indomethacin and expectant management for CLD (RR 0.84, 95% CI 0.52 to 1.37; 2 RCTs, 168 infants).

Very early treatment (initiated within three days) versus expectant management: the review by Mitra 2020a showed that there was no evidence of a difference between very early treatment with indomethacin and expectant management for CLD (RR 1.06, 95% CI 0.61 to 1.83; 4 RCTs, 188 infants).

###### Ibuprofen

Intravenous ibuprofen versus placebo or no treatment: the review by Ohlsson 2020b showed that there was no evidence of a difference between IV ibuprofen and placebo or no treatment for CLD defined as the need for supplemental oxygen at 36 weeks' postmenstrual age (RR 0.99, 95% CI 0.88 to 1.11; 1 RCT, 98 infants), or for CLD defined as the need for supplemental oxygen at 28 days of age (RR 1.09, 95% CI 0.95 to 1.26; 1 RCT, 130 infants).

Ibuprofen versus indomethacin: the review by Ohlsson 2020b showed that there was no evidence of a difference between ibuprofen and indomethacin for CLD defined as the need for supplemental oxygen at 36 weeks' postmenstrual age (RR 1.12, 95% CI 0.77 to 1.61; 3 RCTs, 357 infants), or for CLD defined as the need for supplemental oxygen at 28 days of age (RR 1.20, 95% CI 0.93 to 1.55; 5 RCTs, 292 infants).

Oral ibuprofen versus indomethacin: the review by Ohlsson 2020b showed that there was no evidence of a difference between oral ibuprofen and indomethacin for CLD defined as the need for supplemental oxygen at 28 days of age (RD ‐0.07, 95% CI ‐0.42 to 0.29; 1 RCT, 30 infants), or for CLD (no definition specified for CLD; RD ‐0.00, 95% CI ‐0.44 to 0.44; 1 RCT, 18 infants).

Oral ibuprofen versus IV ibuprofen: the review by Ohlsson 2020b showed that there was no evidence of a difference between oral ibuprofen and IV ibuprofen for CLD (RR 0.82, 95% CI 0.56 to 1.20; 3 RCTs, 236 infants).

High‐dose ibuprofen versus standard‐dose ibuprofen: the review by Ohlsson 2020b showed that there was no evidence of a difference between high‐dose ibuprofen and standard‐dose ibuprofen for CLD (RR 1.60, 95% CI 0.85 to 3.02; 1 RCT, 70 infants).

Early treatment (initiated within seven days) versus expectant management: the review by Mitra 2020a showed that there was no evidence of a difference between early treatment with ibuprofen and expectant management for CLD (RR 0.97, 95% CI 0.56 to 1.29; 2 RCTs, 171 infants).

Very early treatment (initiated within three days) versus expectant management: the review by Mitra 2020a showed that compared to expectant management, very early treatment reduced CLD (RR 0.54, 95% CI 0.35 to 0.83; 2 RCTs, 124 infants).

Echocardiogram‐guided versus standard IV ibuprofen: the review by Ohlsson 2020b showed that there was no evidence of a difference between echocardiogram‐guided and standard IV ibuprofen for CLD (RR 1.35, 95% CI 0.53 to 3.44; 1 RCT, 49 infants).

Continuous infusion versus intermittent bolus of ibuprofen: the review by Ohlsson 2020b showed that there was no evidence of a difference between continuous infusion and intermittent bolus of ibuprofen for CLD (RR 1.1, 95% CI 0.55 to 2.2; 1 RCT, 111 infants).

###### Acetaminophen

Acetaminophen versus ibuprofen: the review by Jasani 2022 showed that there was no evidence of a difference between acetaminophen and ibuprofen for CLD defined as need for supplemental oxygen at 36 weeks' postmenstrual age (RR 0.79, 95% CI 0.45 to 1.38; 2 RCTs, 141 infants), for moderate/severe CLD (RR 0.80, 95% CI 0.22 to 2.87; 1 RCT, 160 infants), or for severe CLD (RR 0.62, 95% CI 0.32 to 1.23; 1 RCT, 90 infants).

Acetaminophen versus indomethacin: the review by Jasani 2022 showed that there was no evidence of a difference between acetaminophen and indomethacin for CLD (RR 1.16, 95% CI 0.77 to 1.75; 2 RCTs, 94 infants).

Late acetaminophen (initiated on day 14 or later) versus placebo: the review by Jasani 2022 showed that there was no evidence of a difference between late acetaminophen and placebo for CLD (RR 1.04, 95% CI 0.07 to 15.76; 1 RCT, 55 infants).

Acetaminophen and ibuprofen combination therapy versus ibuprofen alone: the review by Jasani 2022 showed that there was no evidence of a difference between acetaminophen and ibuprofen combination therapy and ibuprofen alone for CLD (RR 0.80, 95% CI 0.28 to 2.27; 1 RCT, 24 infants).

###### Surgical ligation

Surgical PDA ligation versus medical treatment with indomethacin: the review by Malviya 2013 showed that there was no evidence of a difference between surgical PDA ligation and medical therapy for CLD (RR 1.28, 95% CI 0.83 to 1.98; 1 RCT, 154 infants).

##### Pulmonary haemorrhage

Four Cochane Reviews reported on the outcome of pulmonary haemorrhage. They included the following interventions (Table 12).

**12:** Interventions for symptomatic PDA: pulmonary haemorrhage

		**Comparison**		
		**Indomethacin**	**Ibuprofen**	**Placebo/no treatment**
**Therapy**	**Indomethacin**	‐	‐	Indomethacin vs placebo/no treatment* 0.40, 95% CI 0.14 to 1.16; 1 RCT, N = 92
				Very early treatment (≤ day 3) vs expectant management* 0.59, 95% CI 0.22 to 1.53; 2 RCTs, N = 136
	**Ibuprofen**	Ibuprofen IV/PO vs Indomethacin IV/PO* 0.91, 95% CI 0.40 to 2.04; 4 RCTs, N = 303	Ibuprofen PO vs Ibuprofen IV* 0.14, 95% CI 0.01 to 2.52; 1 RCT, N = 70	Ibuprofen IV vs placebo/no treatment* 0.25, 95% CI 0.03 to 2.18; 1 RCT, N = 136
		Ibuprofen PO vs Indomethacin IV/PO* RD ‐0.22, 95% CI ‐0.51 to 0.07; 1 RCT, N = 21		Very early treatment (≤ day 3) vs expectant management* 0.59, 95% CI 0.24 to 1.49; 2 RCTs, N = 124
	**Acetaminophen**	0.77, 95% CI 0.28 to 2.10; 3 RCTs, N = 347	0.87, 95% CI 0.36 to 2.09; 5 RCTs, N = 442	*Late treatment (≥ day 14)* 2.07, 95% CI 0.20 to 21.56; 1 RCT, N = 55

**CI**: confidence interval; **IV**: intravenous; **PO:** per os; **PDA**: patent ductus arteriosus; **PMA:** post‐menstrual age; **RCT**: randomised controlled trials; **RD**: risk difference; **vs:** versus Reference is the listed comparison therapy, unless otherwise indicated by * Differences between intervention and comparison provided as risk ratio (RR), unless otherwise specified

###### Indomethacin

Indomethacin versus placebo or no treatment: the review by Evans 2021 showed that there was no evidence of a difference between indomethacin and placebo or no treatment for pulmonary haemorrhage (RR 0.40, 95% CI 0.14 to 1.16; 1 RCT, 92 infants).

Very early treatment (initiated within three days) versus expectant management: the review by Mitra 2020a showed that there was no evidence of a difference between very early treatment with indomethacin and expectant management for pulmonary haemorrhage (RR 0.59, 95% CI 0.22 to 1.53; 2 RCTs, 136 infants).

###### Ibuprofen

Intravenous ibuprofen versus placebo or no treatment: the review by Ohlsson 2020b showed that there was no evidence of a difference between IV ibuprofen and placebo or no treatment for pulmonary haemorrhage (RR 0.25, 95% CI 0.03 to 2.18; 1 RCT, 136 infants).

Ibuprofen versus indomethacin: the review by Ohlsson 2020b showed that there was no evidence of a difference between ibuprofen and indomethacin for pulmonary haemorrhage (RR 0.91, 95% CI 0.40 to 2.04; 4 RCTs, 303 infants).

Oral ibuprofen versus indomethacin: the review by Ohlsson 2020b showed that there was no evidence of a difference between oral ibuprofen and indomethacin for pulmonary haemorrhage (RD ‐0.22, 95% CI ‐0.51 to 0.07; 1 RCT, 21 infants).

Oral ibuprofen versus IV ibuprofen: the review by Ohlsson 2020b showed that there was no evidence of a difference between oral ibuprofen and IV ibuprofen for pulmonary haemorrhage (RR 0.14, 95% CI 0.01 to 2.52; 1 RCT, 70 infants).

Very early treatment (initiated within three days) versus expectant management: the review by Mitra 2020a showed that there was no evidence of a difference between very early treatment and expectant management for pulmonary haemorrhage (RR 0.59, 95% CI 0.24 to 1.49; 2 RCTs, 124 infants).

###### Acetaminophen

Acetaminophen versus ibuprofen: the review by Jasani 2022 showed that there was no evidence of a difference between acetaminophen and ibuprofen for pulmonary haemorrhage (RR 0.87, 95% CI 0.36 to 2.095 RCTs, 442 infants).

Acetaminophen versus indomethacin: the review by Jasani 2022 showed that there was no evidence of a difference between acetaminophen and indomethacin for pulmonary haemorrhage (RR 0.77, 95% CI 0.28 to 2.103 RCTs, 347 infants).

Late acetaminophen (initiated on or after day 14) versus placebo: the review by Jasani 2022 showed that there was no evidence of a difference between late acetaminophen and placebo for pulmonary haemorrhage (RR 2.07, 95% CI 0.20 to 21.56;1 RCT, 55 infants).

##### Severe intraventricular haemorrhage (grades III/IV)

Five Cochrane Reviews reported on the outcome of severe IVH. They include the following interventions (Table 13).

**13:** Interventions for symptomatic PDA: severe intraventricular haemorrhage (grade III/IV)

		**Comparison**		
		**Indomethacin**	**Ibuprofen**	**Placebo/no treatment**
**Therapy**	**Indomethacin**	Prolonged vs short course* 0.64, 95% CI 0.36 to 1.12; 4 RCTs, N = 310	‐	Indomethacin vs placebo/no treatment* 0.33, 95% CI 0.01 to 7.45; 1 RCT, N = 24
				Very early treatment (≤ day 3) vs expectant management* 1.00, 95% CI 0.07 to 15; 1 RCT, N = 44
	**Ibuprofen**	Ibuprofen IV/PO vs indomethacin IV/PO* 1.05, 95% CI 0.68 to 1.63 10 RCTs, N = 798	Ibuprofen high‐dose vs standard‐dose* 0.50, 95% CI 0.10 to 2.56; 1 RCT, N = 70	Ibuprofen IV vs placebo/no treatment* 1.00, 95% CI 0.47 to 2.15; 1 RCT, N = 134
		Ibuprofen PO vs indomethacin IV/PO* RD ‐0.04, 95% CI ‐0.14 to 0.05; 2 RCTs, N = 124	Ibuprofen IV: continuous infusion vs intermittent bolus* 0.34, 95% CI 0.01 to 8.15; 1 RCT, N = 111	Very early treatment (≤ day 3) vs expectant management* 0.67, 95% CI 0.11 to 3.98; 2 RCTs, N = 124
				Early treatment (≤ day 7) vs expectant management* 0.83, 95% CI 0.32 to 2.16; 2 RCTs, N = 171
	**Acetaminophen**	1.10, 95% CI 0.28 to 4.32; 2 RCTs, N = 112	0.63, 95% CI 0.28 to 1.43; 6 RCTs, N = 544	‐
	**Ibuprofen + acetaminophen**	‐	1.50, 95% CI 0.30 to 7.43; 1 RCT, N = 24	‐

**CI**: confidence interval; **IV**: intravenous; **PO:** per os; **PDA**: patent ductus arteriosus; **PMA:** post‐menstrual age; **RCT**: randomised controlled trials; **RD**: risk difference; **vs:** versus Reference is the listed comparison therapy, unless otherwise indicated by * Differences between intervention and comparison provided as risk ratio (RR), unless otherwise specified

###### Indomethacin

Indomethacin versus placebo or no treatment: the review by Evans 2021 showed that there was no evidence of a difference between indomethacin and placebo or no treatment for severe IVH (RR 0.33, 95% CI 0.01 to 7.45; 1 RCT, 24 infants).

Prolonged versus short course of indomethacin: the review by Herrera 2007 showed that there was no evidence of a difference between a prolonged and short course of indomethacin for severe IVH (RR 0.64, 95% CI 0.36 to 1.12; 4 RCTs, 310 infants).

Very early treatment (initiated within three days) versus expectant management: the review by Mitra 2020a showed that there was no evidence of a difference between very early treatment with indomethacin and expectant management for severe IVH (RR 1.00, 95% CI 0.07 to 15; 1 RCT, 44 infants).

###### Ibuprofen

Intravenous ibuprofen versus placebo or no treatment: the review by Ohlsson 2020b showed that there was no evidence of a difference between IV ibuprofen and placebo or no treatment for severe IVH (RR 1.00, 95% CI 0.47 to 2.15; 1 RCT, 134 infants).

Ibuprofen versus indomethacin: the review by Ohlsson 2020b showed that there was no evidence of a difference between ibuprofen and indomethacin for severe IVH (RR 1.05, 95% CI 0.68 to 1.63; 10 RCTs, 798 infants).

Oral ibuprofen versus indomethacin: the review by Ohlsson 2020b showed that there was no evidence of a difference between oral ibuprofen and indomethacin for severe IVH (RD ‐0.04, 95% CI ‐0.14 to 0.05; 2 RCT, 124 infants).

High‐dose ibuprofen versus standard‐dose ibuprofen: the review by Ohlsson 2020b showed that there was no evidence of a difference between high‐dose ibuprofen and standard‐dose ibuprofen for severe IVH (RR 0.50, 95% CI 0.10 to 2.56; 1 RCT, 70 infants).

Early treatment (initiated within seven days) versus expectant management: the review by Mitra 2020a showed that there was no evidence of a difference between early treatment with ibuprofen and expectant management for severe IVH (RR 0.83, 95% CI 0.32 to 2.16; 2 RCTs, 171 infants).

Very early treatment (initiated within three days) versus expectant management: the review by Mitra 2020a showed that there was no evidence of a difference between very early treatment with ibuprofen and expectant management for severe IVH (RR 0.67, 95% CI 0.11 to 3.98;2 RCTs, 124 infants).

Continuous infusion versus intermittent bolus of ibuprofen: the review by Ohlsson 2020b showed that there was no evidence of a difference between continuous infusion and intermittent bolus of ibuprofen for severe IVH (RR 0.34, 95% CI 0.01 to 8.15; 1 RCT, 111 infants).

###### Acetaminophen

Acetaminophen versus ibuprofen: the review by Jasani 2022 showed that there was no evidence of a difference between acetaminophen and ibuprofen for severe IVH (RR 0.63, 95% CI 0.28 to 1.43; 6 RCTs, 544 infants).

Acetaminophen versus indomethacin: the review by Jasani 2022 showed that there was no evidence of a difference between acetaminophen and indomethacin for severe IVH (RR 1.10, 95% CI 0.28 to 4.31; 2 RCTs, 112 infants).

Acetaminophen and ibuprofen combination therapy versus ibuprofen alone: the review by Jasani 2022 showed that there was no evidence of a difference between acetaminophen and ibuprofen combination therapy and ibuprofen alone for severe IVH (RR 1.50, 95% CI 0.30 to 7.43; 1 RCT, 24 infants).

##### Retinopathy of prematurity (ROP)

Six Cochrane Reviews reported on the outcome of ROP. They included the following interventions (Table 14).

**14:** Interventions for symptomatic PDA: retinopathy of prematurity

		**Comparison**		
		**Indomethacin**	**Ibuprofen**	**Placebo/no treatment**
**Therapy**	**Indomethacin**	Prolonged vs short course* Any stage ROP 1.04, 95% CI 0.57 to 1.88; 3 RCTs, N = 240	‐	Indomethacin vs placebo/no treatment* Any stage ROP 0.32, 95% CI 0.07 to 1.42; 1 RCT, N = 47 *Severe ROP* 0.96, 95% CI 0.06 to 14.43; 1 RCT, N = 47
				Very early treatment (≤ day 3) vs expectant management* Severe ROP 0.16, 95% CI 0.01 to 2.93; 2 RCTs, N = 136
				Early treatment (≤ day 7) vs expectant management* Severe ROP 0.30, 95% CI 0.02 to 5.34; 1 RCT, N = 41
	**Ibuprofen**	Ibuprofen IV/PO vs indomethacin IV/PO* Any stage ROP 0.81, 95% CI 0.60 to 1.10; 7 RCTs, N = 581	Ibuprofen PO vs Ibuprofen IV* ROP requiring laser treatment 0.59, 95% CI 0.26 to 1.34; 2 RCTs, N = 172	Ibuprofen IV vs placebo/no treatment* Any stage ROP 1.19, 95% CI 0.88 to 1.62; 1 RCT, N = 129 Severe ROP 1.18, 95% CI 0.38 to 3.68; 1 RCT, N = 129
			Ibuprofen high‐dose vs standard‐dose* Any stage ROP 1.00, 95% CI 0.27 to 3.69; 1 RCT, N = 70 *Severe ROP* 2.00, 95% CI 0.19 to 21.06; 1 RCT, N = 70	
		Ibuprofen PO vs indomethacin IV/PO* Any stage ROP RD 0.00, 95% CI ‐0.18 to 0.17; 2 RCTs, N = 71	Ibuprofen IV: echocardiography‐guided vs standard administration* ROP requiring laser treatment 2.25, 95% CI 0.50 to 10.05; 1 RCT, N = 49	Very early treatment (≤ day 3) vs expectant management* Severe ROP 0.80, 95% CI 0.24 to 2.69; 1 RCT, N = 60
			Ibuprofen IV: continuous infusion vs intermittent bolus* Any stage ROP 0.68, 95% CI 0.39 to 1.19; 1 RCT, N = 111 *Severe ROP* 0.34, 95% CI 0.04 to 3.16; 1 RCT, N = 111	Early treatment (≤ day 7) vs expectant management* Severe ROP 1.65, 95% CI 0.51 to 5.31; 1 RCT, N = 105
	**Acetaminophen**	*Severe ROP* 1.32, 95% CI 0.58 to 2.99; 2 RCTs, N = 96	*Severe ROP* 0.43, 95% CI 0.12 to 1.55; 2 RCTs, N = 191 *ROP requiring laser treatment* 0.94, 95% CI 0.48 to 1.85; 3 RCTs, N = 353	*Late treatment (≥ day 14)* *ROP requiring treatment* 3.11, 95% CI 0.34 to 28.09; 1 RCT, N = 55
	**Surgicalligation**	3.8, 95% CI 1.12 to 12.93; 1 RCT, N = 154	‐	‐

**CI** confidence interval; **IV**: intravenous; **PO:** per os; **PDA**: patent ductus arteriosus; **PMA:** post‐menstrual age; **RCT**: randomised controlled trials; **RD**: risk difference; **ROP:** retinopathy of prematurity; **vs:** versus Reference is the listed comparison therapy, unless otherwise indicated by * Differences between intervention and comparison provided as risk ratio (RR), unless otherwise specified Severe ROP refers to stage ≥ 3

###### Indomethacin

Indomethacin versus placebo or no treatment: the review by Evans 2021 showed that there was no evidence of a difference between indomethacin and placebo or no treatment for any stage of ROP (RR 0.32, 95% CI 0.07 to 1.42; 1 RCT, 47 infants), or for severe ROP (≥ stage 3; RR 0.96, 95% CI 0.06 to 14.43; 1 RCT, 47 infants).

Prolonged versus short course of indomethacin: the review by Herrera 2007 showed that there was no evidence of a difference between prolonged and short course of indomethacin for any stage of ROP (RR 1.04, 95% CI 0.57 to 1.88; 3 RCTs, 240 infants).

Early treatment (initiated within seven days) versus expectant management: the review by Mitra 2020a showed that there was no evidence of a difference between early treatment with indomethacin and expectant management for severe ROP (≥ stage 3; RR 0.30, 95% CI 0.02 to 5.34; 1 RCT, 41 infants).

Very early treatment (initiated within three days) versus expectant management: the review by Mitra 2020a showed that there was no evidence of a difference between very early treatment with indomethacin and expectant management for severe ROP (≥ stage 3; RR 0.16, 95% CI 0.01 to 2.93; 2 RCTs, 136 infants).

###### Ibuprofen

IV ibuprofen versus placebo or no treatment: the review by Ohlsson 2020b showed that there was no evidence of a difference between IV ibuprofen and placebo or no treatment for any stage of ROP (RR 1.19, 95% CI 0.88 to 1.62; 1 RCT, 129 infants), or for severe ROP (≥ stage 3; RR 1.18, 95% CI 0.38 to 3.68; 1 RCT, 129 infants).

Ibuprofen versus indomethacin: the review by Ohlsson 2020b showed that there was no evidence of a difference between ibuprofen and indomethacin for any stage of ROP (RR 0.81, 95% CI 0.60 to 1.10; 7 RCTs, 581 infants).

Oral ibuprofen versus indomethacin: the review by Ohlsson 2020b showed that there was no evidence of a difference between oral ibuprofen and indomethacin for any stage of ROP (RD 0.00, 95% CI ‐0.18 to 0.17; 2 RCTs, 71 infants).

Oral ibuprofen versus IV ibuprofen: the review by Ohlsson 2020b showed that there was no evidence of a difference between oral ibuprofen and IV ibuprofen for ROP that required laser treatment (RR 0.59, 95% CI 0.26 to 1.34; 2 RCTs, 172 infants).

High‐dose ibuprofen versus standard‐dose ibuprofen: the review by Ohlsson 2020b showed that there was no evidence of a difference between high‐dose ibuprofen and standard‐dose ibuprofen for any stage of ROP (RR 1.00, 95% CI 0.27 to 3.69; 1 RCT, 70 infants), or for severe ROP (≥ stage 3; RR 2.00, 95% CI 0.19 to 21.06; 1 RCT, 70 infants).

Early treatment (initiated within seven days) versus expectant management: the review by Mitra 2020a showed that there was no evidence of a difference between early treatment with ibuprofen and expectant management for severe ROP (≥ stage 3; RR 1.65, 95% CI 0.51 to 5.31; 1 RCT, 105 infants).

Very early treatment (initiated within three days) versus expectant management: the review by Mitra 2020a showed that there was no evidence of a difference between very early treatment with ibuprofen and expectant management for severe ROP (≥ stage 3; RR 0.80, 95% CI 0.24 to 2.69; 1 RCT, 60 infants).

Echocardiogram‐guided versus standard IV ibuprofen: the review by Ohlsson 2020b showed that there was no evidence of a difference between echocardiogram‐guided and standard IV ibuprofen for ROP that required laser treatment (RR 2.25, 95% CI 0.50 to 10.05; 1 RCT, 49 infants).

Continuous infusion versus intermittent bolus of ibuprofen: the review by Ohlsson 2020b showed that there was no evidence of a difference between continuous infusion and intermittent bolus of ibuprofen for any stage of ROP (RR 0.68, 95% CI 0.39 to 1.19; 1 RCT, 111 infants), or for severe ROP (≥ stage 3; RR 0.34, 95% CI 0.04 to 3.16: 1 RCT, 111 infants).

###### Acetaminophen

Acetaminophen versus ibuprofen: the review by Jasani 2022 showed that there was no evidence of a difference between acetaminophen and ibuprofen for severe ROP (≥ stage 3; RR 0.43, 95% CI 0.12 to 1.55; 2 RCTs, 191 infants), or for ROP that required laser treatment (RR 0.94, 95% CI 0.48 to 1.85; 3 RCTs, 353 infants).

Acetaminophen versus indomethacin: the review by Jasani 2022 showed that there was no evidence of a difference between acetaminophen and indomethacin for severe ROP that required treatment (RR 1.32, 95% CI 0.58 to 2.99; 2 RCTs, 96 infants).

Late acetaminophen versus placebo: the review by Jasani 2022 showed that there was no evidence of a difference between late acetaminophen and placebo for ROP that required treatment (RR 3.11, 95% CI 0.34 to 28.09; 1 RCT, 55 infants).

###### Surgical ligation

Surgical PDA ligation versus medical treatment with indomethacin: the review by Malviya 2013 showed that there was no evidence of a difference between surgical PDA ligation and medical therapy for severe ROP (≥ stage 3; RR 3.8, 95% CI 1.12 to 12.93; 1 RCT, 154 infants).

##### Duration of hospitalisation (days)

Five Cochrane Reviews reported on duration of hospitalisation. They included the following interventions (Table 15).

**15:** Interventions for symptomatic PDA: duration of hospitalisation

** **		**Comparison**		
		**Indomethacin**	**Ibuprofen**	**Placebo/no treatment**
**Therapy**	**Indomethacin**	Prolonged vs short course* 19.60 days, 95% CI ‐2.99 to 42.19; 1 RCT, N = 61	‐	Indomethacin vs placebo/no treatment* ‐14.30 days, 95% CI ‐51.36 to +22.76; 1 RCT, N = 44
				Early treatment (≤ Day 7) vs expectant management* ‐1.00 day, 95% CI ‐12.83 to +10.83; 1 RCT, N = 44
	**Ibuprofen**	Ibuprofen IV/PO vs indomethacin IV/PO* ‐0.69 days ‐4.54 to +3.16; 4 RCTs, N = 368, 95% CI	Ibuprofen high‐dose vs standard‐dose* 21.00 days, 95% CI ‐1.44 to 43.44; 1 RCT, N = 70	Very early treatment (≤ day 3) vs expectant management* ‐6.27 days, 95% CI ‐10.39 to ‐2.14; 2 RCTs, N = 124
		Ibuprofen PO vs indomethacin IV/PO* 4.55 days, 95% CI ‐3.61 to 12.71; 1 RCT, N = 83		
	**Acetaminophen**	‐	2.79 days, 95% CI 0.34 to 5.24; 4 RCTs, N = 361	‐

**CI** confidence interval; **IV**: intravenous; **PO:** per os; **PDA**: patent ductus arteriosus; **PMA:** post‐menstrual age; **RCT**: randomised controlled trials; **RD**: risk difference; **ROP:** retinopathy of prematurity; **vs:** versus Reference is the listed comparison therapy, unless otherwise indicated by * Differences between intervention and comparison provided as risk ratio (RR), unless otherwise specified

###### Indomethacin

Indomethacin versus placebo or no treatment: the review by Evans 2021 showed that there was no evidence of a difference between indomethacin and placebo or no treatment on the duration of hospitalisation (MD ‐14.30 days, 95% CI ‐51.36 to 22.76; 1 RCT, 44 infants).

Prolonged versus short course of indomethacin: the review by Herrera 2007 showed that there was no evidence of a difference between prolonged and short course of indomethacin on the duration of hospitalisation (MD 19.60 days, 95% CI ‐2.99 to 42.19; 1 RCT, 61 infants).

Very early treatment (initiated within three days) versus expectant management: the review by Mitra 2020a showed that there was no evidence of a difference between very early treatment with indomethacin and expectant management on the duration of hospitalisation (MD ‐1.00 day, 95% CI ‐12.83 to 10.83; 1 RCT, 44 infants).

###### Ibuprofen

Ibuprofen versus indomethacin: the review by Ohlsson 2020b showed that there was no evidence of a difference between ibuprofen and indomethacin on the duration of hospitalisation (MD ‐0.69 days, 95% CI ‐4.54 to 3.16; 4 RCTs, 368 infants).

Oral ibuprofen versus indomethacin: the review by Ohlsson 2020b showed that there was no evidence of a difference between oral ibuprofen and indomethacin on the duration of hospitalisation (MD 4.55 days, 95% CI ‐3.61 to 12.71; 1 RCT, 83 infants).

High‐dose ibuprofen versus standard‐dose ibuprofen: the review by Ohlsson 2020b showed that there was no evidence of a difference between high‐dose ibuprofen and standard‐dose ibuprofen on the duration of hospitalisation (MD 21.00 days, 95% CI ‐1.44 to 43.44; 1 RCT, 70 infants).

Very early treatment (initiated within three days) versus expectant management: the review by Mitra 2020a showed that compared to expectant management, very early treatment reduced the duration of hospitalisation (MD ‐6.27 days, 95% CI ‐10.39 to ‐2.14; 2 RCTs, 124 infants).

###### Acetaminophen

Acetaminophen versus ibuprofen: the review by Jasani 2022 showed that compared to ibuprofen, acetaminophen increased the duration of hospitalisation (MD 2.79 days, 95% CI 0.34 to 5.24; 4 RCTs, 361 infants).

##### Moderate/severe neurodevelopmental disability

Three Cochrane Reviews reported on moderate/severe neurodevelopmental disability. They included the following interventions (Table 16).

**16:** Interventions for symptomatic PDA: moderate/severe neurodevelopmental disability

		**Comparison**	
		**Ibuprofen**	**Placebo/no treatment**
**Therapy**	**Indomethacin**	‐	Early treatment (≤ day 7) vs expectant management* Moderate/severe cognitive delay 0.27, 95% CI 0.03 to 2.31; 1 RCT, N = 79; low‐certainty evidence *Moderate/severe motor delay* 0.54, 95% CI 0.05 to 5.71; 1 RCT, N = 79; low‐certainty evidence *Moderate/severe language delay* 0.54, 95% CI 0.10 to 2.78; 1 RCT, N = 79; low‐certainty evidence
	**Ibuprofen**	Ibuprofen PO vs ibuprofen IV* Moderate‐severe cerebral palsy 1.35, 95% CI 0.24 to 7.48; 1 RCT, N = 57	‐
	**Acetaminophen**	*Mental Developmental Index < 70* 1.03, 95% CI 0.41 to 2.59; 1 RCT, N = 61 *Psychomotor Developmental Index < 70* 1.03, 95% CI 0.33 to 3.21; 1 RCT, N = 61 *Moderate‐severe cerebral palsy* 2.07, 95% CI 0.41 to 10.46; 1 RCT, N = 61 *Deafness* 0.34, 95% CI 0.01 to 8.13; 1 RCT, N = 61 *Blindness* 0.34, 95% CI 0.01 to 8.13; 1 RCT, N = 61	‐

**CI** confidence interval; **IV**: intravenous; **PO:** per os; **PDA**: patent ductus arteriosus; **PMA:** post‐menstrual age; **RCT**: randomised controlled trials; **RD**: risk difference; **ROP:** retinopathy of prematurity; **vs:** versus Reference is the listed comparison therapy, unless otherwise indicated by * Differences between intervention and comparison provided as risk ratio (RR), unless otherwise specified; certainty of evidence added when available from review

###### Indomethacin

Very early treatment (initiated within three days) versus expectant management: the review by Mitra 2020a showed that there was no evidence of a difference between very early treatment with indomethacin and expectant management for moderate/severe cognitive delay (RR 0.27, 95% CI 0.03 to 2.31; 1 RCT, 79 infants; low‐ certainty evidence); or moderate/severe motor delay (RR 0.54, 95% CI 0.05 to 5.71; 1 RCT, 79 infants; low‐certainty evidence); or moderate/severe language delay (RR 0.54, 95% CI 0.10 to 2.78; 1 RCT, 79 infants; low‐certainty evidence); when assessed at 18 to 24 months.

###### Ibuprofen

Oral ibuprofen versus IV ibuprofen: the review by Ohlsson 2020b showed that there was no evidence of a difference between oral ibuprofen and IV ibuprofen for moderate/severe cerebral palsy at 18 to 24 months (RR 1.35, 95% CI 0.24 to 7.48; 1 RCT, 57 infants).

###### Acetaminophen

Acetaminophen versus ibuprofen: the review by Jasani 2022 showed that there was no evidence of a difference between acetaminophen and ibuprofen on the Mental Developmental Index (MDI < 70; RR 1.03, 95% CI 0.41 to 2.59; 1 RCT, 61 infants); on the Psychomotor Developmental Index (MDI < 70; RR 1.03, 95% CI 0.33 to 3.21; 1 RCT, 61 infants); for moderate to severe cerebral palsy (RR 2.07, 95% CI 0.41 to 10.46; 1 RCT, 61 infants); for deafness (RR 0.34, 95% CI 0.01 to 8.13; 1 RCT, 61 infants); or for blindness (RR 0.34, 95% CI 0.01 to 8.13; 1 RCT, 61 infants).

##### All‐cause mortality

Seven Cochrane Reviews reported on the outcome of mortality. They included the following interventions (Table 17).

**17:** Interventions for symptomatic PDA: mortality

** **		**Comparison**		
		**Indomethacin**	**Ibuprofen**	**Placebo/no treatment**
**Therapy**	**Indomethacin**	Continuous vs Intermittent bolus* 3.95, 95% CI 0.20 to 76.17; 1 RCT, N = 32	‐	Indomethacin vs placebo/no treatment* 0.78, 95% CI 0.46 to 1.33; 8 RCTs, N = 314; moderate‐certainty evidence
		Prolonged vs short course* 1.36, 95% CI 0.86 to 2.15 ; 5 RCTs, N = 431		Very early treatment (≤ day 3) vs expectant management* 0.92, 95% CI 0.47 to 1.80; 3 RCTs, N = 188
				Early treatment (≤ day 7) vs expectant management* 0.95, 95% CI 0.45 to 1.99; 3 RCTs, N = 195
	**Ibuprofen**	Ibuprofen IV/PO vs indomethacin IV/PO* All‐cause mortality 0.79, 95% CI 0.54 to 1.17; 10 RCTs, N = 697 *Mortality during first 28/30 days after birth* 1.12, 95% CI 0.59 to 2.11; 4 RCTs, N = 333	Ibuprofen PO vs Ibuprofen IV* All‐cause mortality 0.83, 95% CI 0.38 to 1.82; 2 RCTs, N = 188 *Mortality during first 28/30 days after birth* 1.13, 95% CI 0.5 to 2.55; 1 RCT, N = 64	Ibuprofen IV vs placebo/no treatment* 0.80, 95% CI 0.34 to 1.90 ; 1 RCT, N = 136
			Ibuprofen high‐dose vs standard‐dose* 1.02, 95% CI 0.58 to 1.79; 2 RCTs, N = 155	
		Ibuprofen PO vs indomethacin IV/PO* All‐cause mortality RD ‐0.10, 95% CI ‐0.20 to ‐0.0; 4 RCTs, N = 165	*Ibuprofen IV:* echocardiography‐guided vs standard administration* 0.56, 95% CI 0.14 to 2.25; 1 RCT, N = 49	Very early treatment (≤ day 3) vs expectant management* 0.65, 95% CI 0.28 to 1.50; 3 RCTs, N = 305
		Ibuprofen PO vs indomethacin IV/PO* Mortality during first 28/30 days after birth RD ‐0.03, 95% CI ‐0.12 to 0.18; 2 RCTs, N = 66	Ibuprofen IV: continuous infusion vs Intermittent bolus* 1.02, 95% CI 0.07 to 15.87; 1 RCT, N = 111	Early treatment (≤ day 7) vs expectant management* 1.46, 95% CI 0.58, 3.67; 2 RCTs, N = 124
	**Acetaminophen**	0.86, 95% CI 0.39 to 1.92; 2 RCTs, N = 114	*All‐cause mortality* 1.09, 95% CI 0.80 to 1.48; 8 RCTs, N = 734 *Mortality during the first 28 days after birth* 1.17, 95% CI 0.43 to 3.20; 1 RCT, N = 90	
	**Surgical Ligation**	0.67, 95% CI 0.34 to 1.31; 1 RCT, N = 154	‐	‐

**CI** confidence interval; **IV**: intravenous; **PO:** per os; **PDA**: patent ductus arteriosus; **PMA:** post‐menstrual age; **RCT**: randomised controlled trials; **RD**: risk difference; **ROP:** retinopathy of prematurity; **vs:** versus Reference is the listed comparison therapy, unless otherwise indicated by * Differences between intervention and comparison provided as risk ratio (RR), unless otherwise specified Outcomes presented are all‐cause mortality, unless otherwise specified; certainty of evidence added when available from review

###### Indomethacin

Indomethacin versus placebo or no treatment: the review by Evans 2021 showed that there was no evidence of a difference between indomethacin and placebo or no treatment for all‐cause mortality before hospital discharge (RR 0.78, 95% CI 0.46 to 1.33; 8 RCTs, 314 infants; moderate‐certainty evidence).

Prolonged versus short course of indomethacin: the review by Herrera 2007 showed that there was no evidence of a difference between prolonged and short course of indomethacin for mortality (RR 1.36, 95% CI 0.86 to 2.15; 5 RCTs, 431 infants).

Continuous infusion versus intermittent bolus of indomethacin: the review by Görk 2008 showed that there was no evidence of a difference between continuous infusion and intermittent bolus of indomethacin on death during the first 28 days of life (RR 3.95, 95% CI 0.20 to 76.17; 1 RCT, 32 infants).

Early treatment (initiated within seven days) versus expectant management: the review by Mitra 2020a showed that there was no evidence of a difference between early treatment with indomethacin and expectant management for all‐cause mortality during the hospital stay (RR 0.95, 95% CI 0.45 to 1.99; 3 RCTs, 195 infants).

Very early treatment (initiated within three days) versus expectant management: the review by Mitra 2020a showed that there was no evidence of a difference between very early treatment with indomethacin and expectant management for all‐cause mortality during the hospital stay (RR 0.92, 95% CI 0.47 to 1.80; 3 RCTs, 188 infants).

###### Ibuprofen

Intravenous ibuprofen versus placebo or no treatment: the review by Ohlsson 2020b showed that there was no evidence of a difference between IV ibuprofen and placebo or no treatment for mortality (RR 0.80, 95% CI 0.34 to 1.90; 1 RCT, 136 infants).

Ibuprofen versus indomethacin: the review by Ohlsson 2020b showed that there was no evidence of a difference between ibuprofen and indomethacin for all‐cause mortality (RR 0.79, 95% CI 0.54 to 1.17; 10 RCTs, 697 infants); or for neonatal mortality during the first 28 or 30 days of life (RR 1.12, 95% CI 0.59 to 2.11; 4 RCTs, 333 infants). 

Oral ibuprofen versus indomethacin: the review by Ohlsson 2020b showed that there was no evidence of a difference between oral ibuprofen and indomethacin for all‐cause mortality (RD ‐0.10, 95% CI ‐0.20 to ‐0.0; 4 RCTs, 165 infants); and for neonatal mortality during the first 28 or 30 days of life (RD ‐0.03, 95% CI ‐0.12 to 0.18; 2 RCTs, 66 infants).

Oral ibuprofen versus IV ibuprofen: the review by Ohlsson 2020b showed that there was no evidence of a difference between oral ibuprofen and IV ibuprofen for neonatal mortality during the first 28 or 30 days of life (RR 1.13, 95% CI 0.5 to 2.55; 1 RCT, 64 infants); or for mortality during the hospital stay (RR 0.83, 95% CI 0.38 to 1.82; 2 RCTs, 188 infants).

High‐dose ibuprofen versus standard‐dose ibuprofen: the review by Ohlsson 2020b showed that there was no evidence of a difference between high‐dose ibuprofen and standard‐dose ibuprofen for mortality during the hospital stay (RR 1.02, 95% CI 0.58 to 1.79; 2 RCTs, 155 infants).

Early treatment (initiated within seven days) versus expectant management: the review by Mitra 2020a showed that there was no evidence of a difference between early treatment with ibuprofen and expectant management for mortality during the hospital stay (RR 0.65, 95% CI 0.28 to 1.50; 3 RCTs, 305 infants).

Very early treatment (initiated within three days) versus expectant management: the review by Mitra 2020a showed that there was no evidence of a difference between very early treatment with ibuprofen and expectant management for mortality during the hospital stay (RR 1.46, 95% CI 0.58 to 3.67; 2 RCTs, 124 infants).

Echocardiogram‐guided versus standard IV ibuprofen: the review by Ohlsson 2020b showed that there was no evidence of a difference between echocardiogram‐guided and standard IV ibuprofen for mortality during the hospital stay (RR 0.56, 95% CI 0.14 to 2.25; 1 RCT, 49 infants).

Continuous infusion versus intermittent bolus of ibuprofen: the review by Ohlsson 2020b showed that there was no evidence of a difference between continuous infusion and intermittent bolus of ibuprofen for mortality during the hospital stay (RR 1.02, 95% CI 0.07 to 15.87; 1 RCT, 111 infants).

###### Acetaminophen

Acetaminophen versus ibuprofen: the review by Jasani 2022 showed that there was no evidence of a difference between acetaminophen and ibuprofen for mortality during the hospital stay (RR 1.09, 95% CI 0.80 to 1.48; 8 RCTs, 734 infants), or for deaths during the first 28 days of life (RR 1.17, 95% CI 0.43 to 3.20; 1 RCT, 90 infants).

Acetaminophen versus indomethacin: the review by Jasani 2022 showed that there was no evidence of a difference between acetaminophen and indomethacin for mortality during the hospital stay (RR 0.86, 95% CI 0.39 to 1.92; 2 RCTs, 114 infants).

###### Surgical ligation

Surgical PDA ligation versus medical treatment with indomethacin: the review by Malviya 2013 showed that there was no evidence of a difference between surgical PDA ligation and medical therapy for mortality during the hospital stay (RR 0.67, 95% CI 0.34 to 1.31; 1 RCT, 154 infants).

##### Necrotising enterocolitis (NEC)

Seven Cochrane Reviews reported on the outcome of NEC. They included the following interventions (Table 18).

**18:** Interventions for symptomatic PDA: necrotising enterocolitis

** **		**Comparison**		
		**Indomethacin**	**Ibuprofen**	**Placebo/no treatment**
**Therapy**	**Indomethacin**	Continuous vs intermittent bolus* 0.56, 95% CI 0.03 to 12.23; 1 RCT, N = 22	‐	Indomethacin vs placebo/no treatment* 1.27, 95% CI 0.36 to 4.55; 2 RCTs, N = 147; low‐certainty evidence
		Prolonged vs short course* Any‐stage NEC 1.87, 95% CI 1.07 to 3.27; 4 RCTs, N = 310		Very early treatment (≤ day 3) vs expectant management* 0.80, 95% CI 0.18 to 3.49; 2 RCTs, N = 188
				Early treatment (≤ day 7) vs expectant management* 1.56, 95% CI 0.28 to 8.80; 2 RCTs, N = 168
	**Ibuprofen**	Ibuprofen IV/PO vs indomethacin IV/PO* Any‐stage NEC 0.68, 95% CI 0.49 to 0.94; 18 RCTs, N = 1292; moderate‐certainty evidence	Ibuprofen PO vs Ibuprofen IV* Any‐stage NEC 0.86, 95% CI 0.35 to 2.15; 3 RCTs, N = 236	Ibuprofen IV vs placebo/no treatment* Any‐stage NEC 1.84, 95% CI 0.87 to 3.90; 2 RCTs, N = 264; moderate‐certainty evidence
			Ibuprofen high‐dose vs standard‐dose* Any‐stage NEC 1.00, 95% CI 0.40 to 2.50; 2 RCTs, N = 130; low‐certainty evidence	
		Ibuprofen PO vs indomethacin IV/PO* Any‐stage NEC 0.41, 95% CI 0.23 to 0.73; 7 RCTs, N = 249; low‐certainty evidence	Ibuprofen IV: echocardiography‐guided vs standard administration* Any‐stage NEC 0.38, 95% CI 0.08 to 1.86; 1 RCT, N = 49	Very early treatment (≤ day 3) vs expectant management* 1.01, 95% CI 0.42 to 2.44; 1 RCT, N = 49
			Ibuprofen IV: continuous infusion vs Intermittent bolus* Any‐stage NEC 0.44, 95% CI 0.12 to 1.60; 1 RCT, N = 111	Early treatment (≤ day 7) vs expectant management* 2.89, 95% CI 0.84 to 9.95; 3 RCTs, N = 305
	**Acetaminophen**	0.42, 95% CI 0.19 to 0.96; 4 RCTs, N = 384; low‐certainty evidence	1.30, 95% CI 0.87 to 1.94; 10 RCTs, N = 1015; moderate‐certainty evidence	*Late treatment (≥ day 14)* 1.04, 95% CI 0.07 to 15.76; 1 RCT, N = 55; low‐certainty evidence
	**Ibuprofen + Acetaminophen**	‐	0.33, 95% CI 0.01 to 7.45; 1 RCT, N = 24; low‐certainty evidence	‐
	**Surgical Ligation**	0.95, 95% CI 0.29 to 3.15; 1 RCT, N = 154	‐	‐

**CI** confidence interval; **IV**: intravenous; **NEC**: necrotizing enterocolitis; **PO:** per os; **PDA**: patent ductus arteriosus; **PMA:** post‐menstrual age; **RCT**: randomised controlled trials; **RD**: risk difference; **ROP:** retinopathy of prematurity; **vs:** versus Reference is the listed comparison therapy, unless otherwise indicated by * Differences between intervention and comparison provided as risk ratio (RR), unless otherwise specified; certainty of evidence added when available from review Outcomes presented are necrotizing enterocolitis (≥ Bell stage 2), unless otherwise specified

###### Indomethacin

Indomethacin versus placebo or no treatment: the review by Evans 2021 showed that there was no evidence of a difference between indomethacin and placebo or no treatment for NEC (≥ Bell stage 2; RR 1.27, 95% CI 0.36 to 4.55; 2 RCTs, 147 infants; low‐certainty evidence).

Prolonged versus short course of indomethacin: the review by Herrera 2007 showed that compared to a short course of indomethacin, a prolonged course increased the risk of any stage of NEC (RR 1.87, 95% CI 1.07 to 3.27; 4 RCTs, 310 infants).

Continuous infusion versus intermittent bolus of indomethacin: the review by Görk 2008 showed that there was no evidence of a difference between continuous infusion and intermittent bolus of indomethacin for NEC (≥ Bell stage 2; RR 0.56, 95% CI 0.03 to 12.23; 1 RCT, 22 infants).

Early treatment (initiated within seven days) versus expectant management: the review by Mitra 2020a showed that there was no evidence of a difference between early treatment with indomethacin and expectant management for NEC (≥ Bell stage 2; RR 1.56, 95% CI 0.28 to 8.80; 2 RCTs, 168 infants).

Very early treatment (initiated within three days) versus expectant management: the review by Mitra 2020a showed that there was no evidence of a difference between very early treatment with indomethacin and expectant management for NEC (≥ Bell stage 2; RR 0.80, 95% CI 0.18 to 3.49; 2 RCTs, 188 infants).

###### Ibuprofen

IV ibuprofen versus placebo or no treatment: the review by Ohlsson 2020b showed that there was no evidence of a difference between IV ibuprofen and placebo or no treatment for any stage of NEC (RR 1.84, 95% CI 0.87 to 3.90; 2 RCTs, 264 infants; moderate‐certainty evidence).

Ibuprofen versus indomethacin: the review by Ohlsson 2020b showed that compared to indomethacin, ibuprofen reduced the risk of any stage of NEC (RR 0.68, 95% CI 0.49 to 0.94; 18 RCTs, 1292 infants; moderate‐certainty evidence).

Oral ibuprofen versus indomethacin: the review by Ohlsson 2020b showed that compared to indomethacin, oral ibuprofen reduced the risk of any stage of NEC (RR 0.41, 95% CI 0.23 to 0.73; 7 RCTs, 249 infants; low‐certainty evidence).

Oral ibuprofen versus IV ibuprofen: the review by Ohlsson 2020b showed that there was no evidence of a difference between oral ibuprofen and IV ibuprofen for any stage of NEC (RR 0.86, 95% CI 0.35 to 2.15; 3 RCTs, 236 infants).

High‐dose ibuprofen versus standard‐dose ibuprofen: the review by Ohlsson 2020b showed that there was no evidence of a difference between high‐dose ibuprofen and standard‐dose ibuprofen for any stage of NEC (RR 1.00, 95% CI 0.40 to 2.50; 2 RCTs, 130 infants; low‐certainty evidence).

Early treatment (initiated within seven days) versus expectant management: the review by Mitra 2020a showed that there was no evidence of a difference between early treatment with ibuprofen and expectant management for NEC (≥ Bell stage 2; RR 2.89, 95% CI 0.84 to 9.95; 3 RCTs, 305 infants).

Very early treatment (initiated within three days) versus expectant management: the review by Mitra 2020a showed that there was no evidence of a difference between very early treatment with ibuprofen and expectant management for NEC (≥ Bell stage 2; RR 1.01, 95% CI 0.42 to 2.44; 2 RCTs, 124 infants).

Echocardiogram‐guided versus standard IV ibuprofen: the review by Ohlsson 2020b showed that there was no evidence of a difference between echocardiogram‐guided and standard IV ibuprofen for any stage of NEC (RR 0.38, 95% CI 0.08 to 1.86; 1 RCT, 49 infants).

Continuous infusion versus intermittent bolus of ibuprofen: the review by Ohlsson 2020b showed that there was no evidence of a difference between continuous infusion and intermittent bolus of ibuprofen for any stage of NEC (RR 0.44, 95% CI 0.12 to 1.60; 1 RCT, 111 infants).

###### Acetaminophen

Acetaminophen versus ibuprofen: the review by Jasani 2022 showed that there was no evidence of a difference between acetaminophen and ibuprofen for NEC (by radiological diagnosis; RR 1.30, 95% CI 0.87 to 1.94; 10 RCTs, 1015 infants; moderate‐certainty evidence).

Acetaminophen versus indomethacin: the review by Jasani 2022 showed that compared to indomethacin, acetaminophen reduced the risk of NEC (by radiological diagnosis; RR 0.42, 95% CI 0.19 to 0.96; 4 RCTs, 384 infants; low‐certainty evidence).

Late acetaminophen (initiated on or later than day 14) versus placebo: the review by Jasani 2022 showed that there was no evidence of a difference between late acetaminophen and placebo for NEC (by radiological diagnosis; RR 1.04, 95% CI 0.07 to 15.76; 1 RCT, 55 infants; low‐certainty evidence).

Acetaminophen and ibuprofen combination therapy versus ibuprofen alone: the review by Jasani 2022 showed that there was no evidence of a difference between acetaminophen and ibuprofen combination therapy and ibuprofen alone for NEC (by radiological diagnosis; RR 0.33, 95% CI 0.01 to 7.45; 1 RCT, 24 infants; low‐certainty evidence).

###### Surgical ligation

Surgical PDA ligation versus medical treatment with indomethacin: the review by Malviya 2013 showed that there was no evidence of a difference between surgical PDA ligation and medical therapy for NEC (by radiological diagnosis; RR 0.95, 95% CI 0.29 to 3.15; 1 RCT, 154 infants).

##### Gastrointestinal bleeding

Three Cochrane Reviews reported on gastrointestinal bleeding. They included the following interventions (Table 19).

**19:** Interventions for symptomatic PDA: gastrointestinal bleeding

** **		**Comparison**		
		**Indomethacin**	**Ibuprofen**	**Placebo/no treatment**
**Therapy**	**Indomethacin**	‐	‐	Indomethacin vs placebo/no treatment* 0.33, 95% CI 0.01 to 7.58; 2 RCTs, N = 119
	**Ibuprofen**	Ibuprofen IV/PO vs indomethacin IV/PO* 0.94, 95% CI 0.55 to 1.61; 7 RCTs, N = 514	Ibuprofen PO vs ibuprofen IV* 2.89, 95% CI 0.12 to 69.24; 2 RCTs, N = 172	‐
			Ibuprofen high‐dose vs standard‐dose* 1.50, 95% CI 0.58 to 3.86; 2 RCTs, N = 120	
		Ibuprofen PO vs indomethacin IV/PO* RD 0.07, 95% CI ‐0.05 to 0.18; 3 RCTs, N = 85	Ibuprofen IV: continuous infusion vs intermittent bolus* 0.51, 95% CI 0.16 to1.59; 1 RCT, N = 111	
	**Acetaminophen**	0.63, 95% CI 0.32 to 1.25; 3 RCTs, N = 347	RD ‐0.05, 95% CI ‐0.09 to ‐0.02; 7 RCTs, N = 693	‐

**CI** confidence interval; **IV**: intravenous; **PO:** per os; **PDA**: patent ductus arteriosus; **PMA:** post‐menstrual age; **RCT**: randomised controlled trials; **RD**: risk difference; **ROP:** retinopathy of prematurity; **vs:** versus Reference is the listed comparison therapy, unless otherwise indicated by * Differences between intervention and comparison provided as risk ratio (RR), unless otherwise specified

###### Indomethacin

Indomethacin versus placebo or no treatment: the review by Evans 2021 showed that there was no evidence of a difference between indomethacin and placebo or no treatment for gastrointestinal bleeding (RR 0.33, 95% CI 0.01 to 7.58; 2 RCTs, 119 infants; low‐certainty evidence).

###### Ibuprofen

Ibuprofen versus indomethacin: the review by Ohlsson 2020b showed that there was no evidence of a difference between ibuprofen and indomethacin for gastrointestinal bleeding (RR 0.94, 95% CI 0.55 to 1.61; 7 RCTs, 514 infants).

Oral ibuprofen versus indomethacin: the review by Ohlsson 2020b showed that there was no evidence of a difference between oral ibuprofen and indomethacin for gastrointestinal bleeding (RD 0.07, 95% CI ‐0.05 to 0.18; 3 RCTs, 85 infants).

Oral ibuprofen versus IV ibuprofen: the review by Ohlsson 2020b showed that there was no evidence of a difference between oral ibuprofen and IV ibuprofen for gastrointestinal bleeding (RR 2.89, 95% CI 0.12 to 69.24; 2 RCTs, 172 infants).

High‐dose ibuprofen versus standard‐dose ibuprofen: the review by Ohlsson 2020b showed that there was no evidence of a difference between high‐dose ibuprofen and standard‐dose ibuprofen for gastrointestinal bleeding (RR 1.50, 95% CI 0.58 to 3.86; 2 RCTs, 120 infants).

Continuous infusion versus intermittent bolus of ibuprofen: the review by Ohlsson 2020b showed that there was no evidence of a difference between continuous infusion and intermittent bolus of ibuprofen for gastrointestinal bleeding (RR 0.51, 95% CI 0.16 to 1.59; 1 RCT, 111 infants).

###### Acetaminophen

Acetaminophen versus ibuprofen: the review by Jasani 2022 showed that compared to ibuprofen, acetaminophen reduced gastrointestinal bleeding (RD ‐0.05, 95% CI ‐0.09 to ‐0.02; 7 RCTs, 693 infants).

Acetaminophen versus indomethacin: the review by Jasani 2022 showed that there was no evidence of a difference between acetaminophen and indomethacin for gastrointestinal bleeding (RR 0.63, 95% CI 0.32 to 1.25; 3 RCTs, 347 infants).

##### Gastrointestinal perforation

Four Cochrane Reviews reported on gastrointestinal perforation. They included the following interventions (Table 20).

**20:** Interventions for symptomatic PDA: gastrointestinal perforation

		**Comparison**		
		**Indomethacin**	**Ibuprofen**	**Placebo/no treatment**
**Therapy**	**Indomethacin**	‐	‐	Indomethacin vs placebo/no treatment* 0.98, 95% CI 0.06 to 15.40; 1 RCT, N = 127
	**Ibuprofen**	Ibuprofen IV/PO vs indomethacin IV/PO* 0.48, 95% CI 0.20 to 1.14; 5 RCTs, N = 255	Ibuprofen PO vs ibuprofen IV* 0.32, 95% CI 0.01 to 7.48; 2 RCTs, N = 134	Very early treatment (≤ day 3) vs expectant management* 0.50, 95% CI 0.05 to 5.24; 1 RCT, N = 64
		Ibuprofen PO vs indomethacin IV/PO* RD ‐0.01, 95% CI ‐0.25 to 0.04; 2 RCTs, N = 62	Ibuprofen IV: continuous infusion vs intermittent bolus* 2.04, 95% CI 0.19 to 21.82; 1 RCT, N = 111	Early treatment (≤ day 7) vs expectant management* 0.47, 95% CI 0.09 to 2.47; 2 RCTs, N = 171
	**Acetaminophen**	‐	2.83, 95% CI 0.12 to 67.87; 2 RCTs, N = 191	‐

**CI** confidence interval; **IV**: intravenous; **NEC**: necrotizing enterocolitis; **PO:** per os; **PDA**: patent ductus arteriosus; **PMA:** post‐menstrual age; **RCT**: randomised controlled trials; **RD**: risk difference; **ROP:** retinopathy of prematurity; **vs:** versus Reference is the listed comparison therapy, unless otherwise indicated by * Differences between intervention and comparison provided as risk ratio (RR), unless otherwise specified

###### Indomethacin

Indomethacin versus placebo or no treatment: the review by Evans 2021 showed that there was no evidence of a difference between indomethacin and placebo or no treatment for gastrointestinal perforation (RR 0.98, 95% CI 0.06 to 15.40; 1 RCT, 127 infants).

###### Ibuprofen

Ibuprofen versus indomethacin: the review by Ohlsson 2020b showed that there was no evidence of a difference between ibuprofen and indomethacin for gastrointestinal perforation (RR 0.48, 95% CI 0.20 to 1.14; 5 RCTs, 255 infants).

Oral ibuprofen versus indomethacin: the review by Ohlsson 2020b showed that there was no evidence of a difference between oral ibuprofen and indomethacin for gastrointestinal perforation (RD ‐0.01, 95% CI ‐0.25 to 0.04; 2 RCTs, 62 infants).

Oral ibuprofen versus IV ibuprofen: the review by Ohlsson 2020b showed that there was no evidence of a difference between oral ibuprofen and IV ibuprofen for gastrointestinal perforation (RR 0.32, 95% CI 0.01 to 7.48; 2 RCTs, 134 infants).

Early treatment (initiated within seven days) versus expectant management: the review by Mitra 2020a showed that there was no evidence of a difference between early treatment with ibuprofen and expectant management for gastrointestinal perforation (RR 0.47, 95% CI 0.09 to 2.47; 2 RCTs, 171 infants).

Very early treatment (initiated within three days) versus expectant management: the review by Mitra 2020a showed that there was no evidence of a difference between very early treatment with ibuprofen and expectant management for gastrointestinal perforation (RR 0.50, 95% CI 0.05 to 5.24; 1 RCT, 64 infants).

Continuous infusion versus intermittent bolus of ibuprofen: the review by Ohlsson 2020b showed that there was no evidence of a difference between continuous infusion and intermittent bolus of ibuprofen for gastrointestinal perforation (RR 2.04, 95% CI 0.19 to 21.82; 1 RCT, 111 infants).

###### Acetaminophen

Acetaminophen versus ibuprofen: the review by Jasani 2022 showed that there was no evidence of a difference between acetaminophen and ibuprofen for gastrointestinal perforation (RR 2.83, 95% CI 0.12 to 67.87; 2 RCTs, 191 infants).

##### Oliguria

Five Cochrane Reviews reported on the outcome of oliguria. They included the following interventions (Table 21).

**21:** Interventions for symptomatic PDA: oliguria

		**Comparison**		
		**Indomethacin**	**Ibuprofen**	**Placebo/no treatment**
**Therapy**	**Indomethacin**	Prolonged vs short course* 0.27, 95% CI 0.13 to 0.60; 2 RCTs, N = 197	‐	Very early treatment (≤ day 3) vs expectant management* 5.00, 95% CI 0.63 to 39.39; 1 RCT, N = 44
				Early treatment (≤ day 7) vs expectant management* 4.59, 95% CI 1.39 to 15.21; 1 RCT, N = 127
	**Ibuprofen**	Ibuprofen IV/PO vs indomethacin IV/PO* 0.28, 95% CI 0.14 to 0.54; 6 RCTs, N = 576; moderate‐certainty evidence	Ibuprofen PO vs ibuprofen IV* 0.14, 95% CI 0.01 to 2.66; 4 RCTs, N = 304; low‐certainty evidence	Ibuprofen IV vs placebo/no treatment* 39.00, 95% CI 2.40 to 633.01; 1 RCT, N = 134
			Ibuprofen high‐dose vs standard‐dose* Urine output < 0.5 mL/kg/hour 1.57, 95% CI 0.44 to 5.63; 2 RCTs, N = 120; low‐certainty evidence *Urine output < 1 mL/kg/hour* 1.50, 95% CI 0.27 to 8.43; 1 RCT, N = 70	
		Ibuprofen PO vs indomethacin IV/PO* RD 0.00, 95% CI ‐0.10 to 0.10; 1 RCT, N = 36	Ibuprofen IV: echocardiography‐guided vs standard administration* 5.31, 95% CI 0.29 to 97.57; 1 RCT, N = 49	Early treatment (≤ day 7) vs expectant management* 39.00, 95% CI 2.40 to 633.01; 1 RCT, N = 134
			Ibuprofen IV: continuous infusion vs intermittent bolus* 0.51, 95% CI 0.05 to 5.45; 1 RCT, N = 111	
	**Acetaminophen**	‐	0.47, 95% CI 0.30 to 0.76; 5 RCTs, N = 608	‐
	**Ibuprofen + Acetaminophen**	‐	0.50, 95% CI 0.05 to 4.81; 1 RCT, N = 24	‐
	**Indomethacin + dopamine**	0.73, 95% CI 0.35 to 1.54; 1 RCT, N = 33	‐	‐

**CI** confidence interval; **IV**: intravenous; **PO:** per os; **PDA**: patent ductus arteriosus; **PMA:** post‐menstrual age; **RCT**: randomised controlled trials; **RD**: risk difference; **ROP:** retinopathy of prematurity; **vs:** versus Reference is the listed comparison therapy, unless otherwise indicated by * Differences between intervention and comparison provided as risk ratio (RR), unless otherwise specified; certainty of evidence added when available from review Oliguria defined as urine output < 1 mL/kg/hour, unless otherwise specified

###### Indomethacin

Prolonged versus short course of indomethacin: the review by Herrera 2007 showed that compared to a short course of indomethacin, a prolonged course reduced oliguria (urine output < 1 mL/kg/hour; RR 0.27, 95% CI 0.13 to 0.60; 2 RCTs, 197 infants).

Early treatment (initiated within seven days) versus expectant management: the review by Mitra 2020a showed that compared to expectant management, early treatment with indomethacin increased oliguria (urine output < 1 mL/kg/hour; RR 4.59, 95% CI 1.39 to 15.21; 1 RCT, 127 infants).

Very early treatment (initiated within three days) versus expectant management: the review by Mitra 2020a showed that there was no evidence of a difference between very early treatment with indomethacin and expectant management for oliguria (urine output < 1 mL/kg/hour; RR 5.00, 95% CI 0.63 to 39.39; 1 RCT, 44 infants).

###### Ibuprofen

IV ibuprofen versus placebo or no treatment: the review by Ohlsson 2020b showed that compared to placebo or no treatment, IV ibuprofen increased oliguria (urine output < 1 mL/kg/hour; RR 39.00, 95% CI 2.40 to 633.01; 1 RCT, 134 infants).

Ibuprofen versus indomethacin: the review by Ohlsson 2020b showed that compared to indomethacin, ibuprofen reduced oliguria (urine output < 1 mL/kg/hour; RR 0.28, 95% CI 0.14 to 0.54; 6 RCTs, 576 infants; moderate‐certainty evidence).

Oral ibuprofen versus indomethacin: the review by Ohlsson 2020b showed that there was no evidence of a difference between oral ibuprofen and indomethacin for oliguria (RD 0.00, 95% CI ‐0.10 to 0.10; 1 RCT, 36 infants).

Oral ibuprofen versus IV ibuprofen: the review by Ohlsson 2020b showed that there was no evidence of a difference between oral ibuprofen and IV ibuprofen for oliguria (urine output < 1 mL/kg/hour; RR 0.14, 95% CI 0.01 to 2.66; 4 RCTs, 304 infants; low‐certainty evidence).

High‐dose ibuprofen versus standard‐dose ibuprofen: the review by Ohlsson 2020b showed that there was no evidence of a difference between high‐dose ibuprofen and standard‐dose ibuprofen for oliguria defined as urine output < 0.5 mL/kg/hour (RR 1.57, 95% CI 0.44 to 5.63; 2 RCTs, 120 infants; low‐certainty evidence); or oliguria defined as urine output < 1 mL/kg/hour (RR 1.50, 95% CI 0.27 to 8.43; 1 RCT, 70 infants).

Early treatment (initiated within seven days) versus expectant management: the review by Mitra 2020a showed that compared to expectant management, early treatment with ibuprofen increased oliguria (urine output < 1 mL/kg/hour; RR 39.00, 95% CI 2.40 to 633.01; 1 RCT, 134 infants).

Echocardiogram‐guided versus standard IV ibuprofen: the review by Ohlsson 2020b showed that there was no evidence of a difference between echocardiogram‐guided and standard IV ibuprofen for oliguria (urine output < 1 mL/kg/hour; RR 5.31, 95% CI 0.29 to 97.57; 1 RCT, 49 infants).

Continuous infusion versus intermittent bolus of ibuprofen: the review by Ohlsson 2020b showed that there was no evidence of a difference between continuous infusion and intermittent bolus of ibuprofen for oliguria (urine output < 1 mL/kg/hour; RR 0.51, 95% CI 0.05 to 5.45; 1 RCT, 111 infants).

###### Acetaminophen

Acetaminophen versus ibuprofen: the review by Jasani 2022 showed that compared to ibuprofen, acetaminophen reduced oliguria (urine output < 1 mL/kg/hour; RR 0.47, 95% CI 0.30 to 0.76; 5 RCTs, 608 infants).

Acetaminophen and ibuprofen combination therapy versus ibuprofen alone: the review by Jasani 2022 showed that there was no evidence of a difference between acetaminophen and ibuprofen combination therapy and ibuprofen alone for oliguria (RR 0.50, 95% CI 0.05 to 4.81; 1 RCT, 24 infants).

###### Adjunct therapies

Dopamine versus control: the review by Barrington 2002 showed that there was no evidence of a difference between the combination of dopamine and indomethacin versus indomethacin alone for oliguria (RR 0.73, 95% CI 0.35 to 1.54; 1 RCT, 33 infants).

##### Serum/plasma levels of creatinine after treatment

Two Cochrane Reviews reported on this outcome. They included the following interventions (Table 22).

**22:** Interventions for symptomatic PDA: serum/plasma levels of creatinine after treatment

** **		**Comparison**		
		**Indomethacin**	**Ibuprofen**	**Placebo/no treatment**
	**Ibuprofen**	Ibuprofen IV/PO vs indomethacin IV/PO* ‐8.12 µmol/L, 95% CI ‐10.81 to ‐5.43; 11 RCTs, N = 918; low‐certainty evidence	Ibuprofen PO vs ibuprofen IV* ‐22.47 µmol/L, 95% CI ‐32.40 to ‐12.53; 2 RCTs, N = 170; low‐certainty evidence	Ibuprofen IV vs placebo/no treatment* 29.17 µmol/L, 95% CI 12.60 to 45.74; 1 RCT, N = 134
			Ibuprofen high‐dose vs standard‐dose* 8.84 µmol/L, 95% CI ‐4.41 to 22.09; 1 RCT, N = 60	
		Ibuprofen PO vs indomethacin IV/PO* ‐0.51 µmol/L, 95% CI ‐6.04 to 5.01; 5 RCTs, N = 190; very low‐certainty evidence	Ibuprofen IV: echocardiography‐guided vs standard administration* ‐11.49 µmol/L, 95% CI ‐29.88 to 6.90; 1 RCT, N = 49	
			Ibuprofen IV: continuous infusion vs intermittent bolus* 2.10 µmol/L, 95% CI ‐4.92 to 9.12; 1 RCT, N = 111	
			Ibuprofen PR vs PO* ‐6.18 µmol/L, 95% CI ‐7.22 to ‐5.14; 1 RCT, N = 72	
	**Indomethacin + dopamine**	2.04 µmol/L, 95% CI ‐17.90 to +21.97; 2 RCTs, N = 59	‐	‐

**CI** confidence interval; **IV**: intravenous; **PO:** per os; **PDA**: patent ductus arteriosus; **PMA:** post‐menstrual age; **PR**: per rectum; **RCT**: randomised controlled trials; **RD**: risk difference; **ROP:** retinopathy of prematurity; **vs:** versus Reference is the listed comparison therapy, unless otherwise indicated by * Differences between intervention and comparison provided as risk ratio (RR), unless otherwise specified; certainty of evidence added when available from review

###### Ibuprofen

IV ibuprofen versus placebo or no treatment: the review by Ohlsson 2020b showed that compared to placebo or no treatment, IV ibuprofen increased serum creatinine post‐treatment (MD 29.17 µmol/L, 95% CI 12.60 to 45.741 RCT, 134 infants).

Ibuprofen versus indomethacin: the review by Ohlsson 2020b showed that compared to indomethacin, ibuprofen reduced serum creatinine post‐treatment (MD ‐8.12 µmol/L, 95% CI ‐10.81 to ‐5.43; 11 RCTs, 918 infants; low‐certainty evidence).

Oral ibuprofen versus indomethacin: the review by Ohlsson 2020b showed that there was no evidence of a difference between oral ibuprofen and indomethacin for serum creatinine post‐treatment (MD ‐0.51 µmol/L, 95% CI ‐6.04 to 5.01; 5 RCTs, 190 infants; very low‐certainty evidence).

Oral ibuprofen versus IV ibuprofen: the review by Ohlsson 2020b showed that compared to IV ibuprofen, oral ibuprofen reduced serum creatinine post‐treatment (MD ‐22.47 µmol/L, 95% CI ‐32.40 to ‐12.53; 2 RCTs, 170 infants; low certainty evidence).

High‐dose ibuprofen versus standard‐dose ibuprofen: the review by Ohlsson 2020b showed that there was no evidence of a difference between high‐dose ibuprofen and standard‐dose ibuprofen for serum creatinine post‐treatment (MD 8.84 µmol/L, 95% CI ‐4.41 to 22.09; 1 RCT, 60 infants).

Echocardiogram‐guided versus standard IV ibuprofen: the review by Ohlsson 2020b showed that there was no evidence of a difference between echocardiogram‐guided and standard IV ibuprofen for serum creatinine post‐treatment (MD ‐11.49 µmol/L, 95% CI ‐29.88 to 6.90; 1 RCT, 49 infants).

Continuous infusion versus intermittent bolus of ibuprofen: the review by Ohlsson 2020b showed that there was no evidence of a difference between continuous infusion and intermittent bolus of ibuprofen for serum creatinine post‐treatment (MD 2.10 µmol/L, 95% CI ‐4.92 to 9.12; 1 RCT, 111 infants).

Rectal ibuprofen versus oral ibuprofen: the review by Ohlsson 2020b showed that compared to oral ibuprofen, rectal ibuprofen reduced serum creatinine post‐treatment (MD ‐6.18 µmol/L, 95% CI ‐7.22 to ‐5.14; 1 RCT, 72 infants).

###### Adjunct therapies

Dopamine versus control: the review by Barrington 2002 showed that there was no evidence of a difference between the combination of dopamine and indomethacin versus indomethacin alone for serum creatinine post‐treatment (MD 2.04 µmol/L, 95% CI ‐17.90 to 21.97; 2 RCTs, 59 infants).

##### Increase in serum/plasma levels of creatinine after treatment

Three Cochrane Reviews reported on this outcome. They included the following interventions (Table 23).

**23:** Interventions for symptomatic PDA: increase in serum/plasma levels of creatinine after treatment

		**Comparison**	
		**Indomethacin**	**Ibuprofen**
	**Ibuprofen**	Ibuprofen IV/PO vs indomethacin IV/PO* ‐15.91 µmol/L, 95% CI ‐31.78 to ‐0.04; 1 RCT, N = 21	‐
	**Acetaminophen**	‐32.71 µmol/L, 95% CI ‐35.36 to ‐30.06; 2 RCTs, N = 270	‐10.61 µmol/L, 95% CI ‐11.49 to ‐8.84; 6 RCTs, N = 557
	**Furosemide + indomethacin**	‐0.88 µmol/L, 95% CI ‐12.38 to 10.61; 3 RCTs, N = 70	‐

**CI** confidence interval; **IV**: intravenous; **PO:** per os; **PDA**: patent ductus arteriosus; **PMA:** post‐menstrual age; **PR**: per rectum; **RCT**: randomised controlled trials; **RD**: risk difference; **ROP:** retinopathy of prematurity; **vs:** versus Reference is the listed comparison therapy, unless otherwise indicated by * Differences between intervention and comparison provided as mean difference (MD), unless otherwise specified

###### Ibuprofen

Ibuprofen versus indomethacin: the review by Ohlsson 2020b showed that compared to indomethacin, ibuprofen led to a lower increase in serum creatinine post‐treatment (MD ‐15.91 µmol/L, 95% CI ‐31.78 to ‐0.04; 1 RCT, 21 infants).

###### Acetaminophen

Acetaminophen versus ibuprofen: the review by Jasani 2022 showed that compared to ibuprofen, acetaminophen led to a lower increase in serum creatinine post‐treatment (MD ‐10.61 µmol/L, 95% CI ‐11.49 to ‐8.84; 6 RCTs, 557 infants).

Acetaminophen versus indomethacin: the review by Jasani 2022 showed that compared to indomethacin, acetaminophen led to a lower increase in serum creatinine post‐treatment (MD ‐32.71 µmol/L, 95% CI ‐35.36 to ‐30.06; 2 RCTs, 270 infants).

###### Adjunct therapies

Furosemide versus control: the review by Brion 2001 showed that there was no evidence of a difference between the combination of furosemide and indomethacin versus indomethacin alone for increase in serum creatinine post‐treatment (MD ‐0.88 µmol/L, 95% CI ‐12.38 to 10.61; 3 RCTs, 70 infants).

##### Serum/plasma levels of bilirubin after treatment

Two Cochrane Reviews reported on this outcome. They included the following interventions (Table 24).

**24:** Interventions for symptomatic PDA: serum/plasma levels of bilirubin after treatment

		**Comparison**	
		**Indomethacin**	**Ibuprofen**
	**Ibuprofen**	Ibuprofen IV/PO vs indomethacin IV/PO* 12.65 µmol/L, 95% CI 9.96 to 15.34; 1 RCT, N = 200	Ibuprofen PR vs PO* 7.01 µmol/L, 95% CI ‐11.23 to 25.25; 1 RCT, N = 72
	**Acetaminophen**	1.03 µmol/L, 95% CI 0.13 to 1.93; 1 RCT, N = 200	‐10.56 µmol/L, 95% CI ‐13.16 to ‐7.96; 4 RCTs, N = 400

**CI** confidence interval; **IV**: intravenous; **PO:** per os; **PDA**: patent ductus arteriosus; **PMA:** post‐menstrual age; **PR**: per rectum; **RCT**: randomised controlled trials; **RD**: risk difference; **ROP:** retinopathy of prematurity; **vs:** versus Reference is the listed comparison therapy, unless otherwise indicated by * Differences between intervention and comparison provided as mean difference (MD), unless otherwise specified

###### Ibuprofen

Ibuprofen versus indomethacin: the review by Ohlsson 2020b showed that compared to indomethacin, ibuprofen increased serum bilirubin levels post‐treatment (MD 12.65 µmol/L, 95% CI 9.96 to 15.34; 1 RCT, 200 infants).

Rectal ibuprofen versus oral ibuprofen: the review by Ohlsson 2020b showed that there was no evidence of a difference between rectal ibuprofen and oral ibuprofen for serum bilirubin levels post‐treatment (MD 7.01 µmol/L, 95% CI ‐11.23 to 25.25; 1 RCT, 72 infants).

###### Acetaminophen

Acetaminophen versus ibuprofen: the review by Jasani 2022 showed that compared to ibuprofen, acetaminophen reduced serum bilirubin levels post‐treatment (MD ‐10.56 µmol/L, 95% CI ‐13.16 to ‐7.96; 4 RCTs, 400 infants).

Acetaminophen versus indomethacin: the review by Jasani 2022 showed that compared to indomethacin, acetaminophen increased serum bilirubin levels post‐treatment (MD 1.03 µmol/L, 95% CI 0.13 to 1.93; 1 RCT, 200 infants).

##### Increase in serum/plasma levels of bilirubin after treatment

No review reported on this outcome.

#### Subgroup Analyses

None of the reviews provided data on any of our pre‐specified subgroups.

## Discussion

### Summary of main results

We included 16 Cochrane Reviews (138 randomised controlled trials (RCTs), 11,856 preterm infants) on the management of patent ductus arteriosis (PDA) in preterm infants. The number of trials included in each review ranged from none to 39. Six reviews (N = 4976) reported on prophylactic interventions for the prevention of PDA, and included pharmacological prophylaxis with prostaglandin inhibitor drugs (indomethacin, ibuprofen, and acetaminophen) and prophylactic surgical PDA ligation and non‐pharmacologic interventions (chest shielding during phototherapy and restriction of fluid intake). One review (N = 97) reported on the use of indomethacin for the management of asymptomatic PDA. Nine reviews (N = 6783) reported on interventions for the management of symptomatic PDA, and included pharmacotherapy with prostaglandin inhibitor drugs (indomethacin, ibuprofen and acetaminophen) in various routes and dosages; surgical PDA ligation; and adjunct therapies (use of furosemide and dopamine in conjunction with indomethacin). The certainty of the evidence, when reported by the respective reviews for the primary outcomes for prevention of PDA, ranged from moderate to low, while those for the primary outcomes for treatment of PDA, ranged from high to low.

#### Interventions for prevention of PDA and related complications in preterm infants

Prophylactic indomethacin probably reduces severe intraventricular haemorrhage (IVH), while it does not appear to affect the composite outcome of death or moderate/severe neurodevelopmental disability. Prophylactic ibuprofen probably marginally reduces severe IVH (moderate‐certainty evidence), while the evidence is very uncertain on the effect of prophylactic acetaminophen on severe IVH. There is no evidence on the effect of either prophylactic ibuprofen or acetaminophen on the composite outcome of death or moderate/severe neurodevelopmental disability. There is a paucity of evidence for any other prophylactic intervention on the primary outcomes of severe IVH and the composite of death or moderate/severe neurodevelopmental disability.

With respect to other patient‐important outcomes, both prophylactic indomethacin and ibuprofen (moderate‐certainty evidence) reduced the need for invasive PDA closure. Necrotising enterocolitis (NEC) appeared to be lower with both prophylactic surgical ligation and fluid restriction. There was no effect of the other prophylactic interventions on any other clinically relevant outcomes, such as mortality or chronic lung disease (CLD).

#### Interventions for management of asymptomatic PDA in preterm infants

Overall evidence is limited (3 RCTs, 97 infants) for the management of asymptomatic PDA. Treatment of asymptomatic PDA with indomethacin appears to reduce the development of symptomatic PDA post‐treatment. There is no evidence on the effect of asymptomatic PDA treatment on the composite outcome of death or moderate/severe neurodevelopmental disability.

#### Interventions for management of symptomatic PDA in preterm infants

All available prostaglandin inhibitor drugs appear to be more effective in PDA closure when compared to placebo or no treatment (high‐certainty evidence for indomethacin; moderate‐certainty evidence for ibuprofen; low‐certainty evidence for early administration of acetaminophen). Oral ibuprofen appears to be more effective in PDA closure compared to ibuprofen (moderate‐certainty evidence); and high‐dose ibuprofen appears to be more effective in PDA closure compared to standard‐dose ibuprofen (moderate‐certainty evidence). There was no evidence of any difference in PDA closure effectiveness between the three available prostaglandin inhibitor drugs (low‐ to moderate‐certainty evidence). There is no evidence on the effect of treatment of symptomatic PDA on the composite outcome of death or moderate/severe neurodevelopmental disability.

From a safety perspective, compared to indomethacin administration, NEC appears to be lower with ibuprofen (any route; moderate‐certainty evidence), exclusive oral administration of ibuprofen (low‐certainty evidence), and with acetaminophen (low‐certainty evidence). On the contrary, NEC appears to be more common with a prolonged course of indomethacin versus a shorter course. Oliguria is also higher with use of indomethacin versus ibuprofen (moderate‐certainty evidence), the use of ibuprofen versus either placebo or acetaminophen, and with early pharmacological treatment of PDA, initiated within the first seven days of life versus later treatment.

### Overall completeness and applicability of evidence

We found reviews for all our prespecified objectives. However, there was substantial variation in the certainty of available evidence for the different interventions for patient‐important outcomes. For prophylactic interventions, the precision of the estimate of effects is best with indomethacin, while the evidence is limited for ibuprofen and sparse for acetaminophen. Evidence from RCTs does suggest a definite benefit with prophylactic indomethacin, and a probable benefit with prophylactic ibuprofen with a reduction in severe IVH. However, the results should be interpreted with caution, as several of the RCTs contributing to the reviews on prophylactic indomethacin and ibuprofen were conducted more than 20 years ago, when NICU practices were vastly different, including the use of antenatal corticosteroids, approaches to mechanical ventilation, and the use of surfactant. It is unclear whether the treatment effects shown in these trials still apply today to extremely preterm infants at higher risk of severe IVH. From a safety perspective, neither indomethacin nor ibuprofen prophylaxis was shown to increase patient‐important adverse outcomes, such as NEC or gastrointestinal perforation. However, the trials included in the respective reviews did not consider the effect of co‐administration of other drugs that might cause harm. This might be an important consideration for clinicians, especially with the emergence of newer prophylactic therapies, such as prophylactic hydrocortisone. A recent individual patient data (IPD) meta‐analysis of RCTs showed that prophylactic low‐dose hydrocortisone can improve survival without CLD (adjusted odds ratio (OR) 1.48, 95% CI 1.02 to 2.16), however, the concomitant use of prophylactic indomethacin and hydrocortisone increases the risk of gastrointestinal perforation (OR 2.50; 95% CI, 1.33 to 4.69; Shaffer 2019). The largest trial contributing to the said IPD meta‐analysis, the PREMILOC trial (N = 1072), failed to demonstrate similar harm in the subgroup of infants who were co‐administered hydrocortisone and ibuprofen (47% of infants enroled in the intervention arm of the trial received ibuprofen (Baud 2016)). Therefore, clinicians should weigh the current applicability of existing evidence for benefit against the potential for harm with concomitant use of other medications, while considering the use of prophylactic non‐steroidal anti‐inflammatory drugs (NSAIDs) in preterm infants. Similarly, clinicians should exercise caution while considering non‐pharmacologic interventions, such as prophylactic fluid restriction to prevent a symptomatic PDA, given the trials were conducted between 1980 and 2000 in moderately preterm infants, and therefore, may not be applicable to extremely preterm infants in the current context. Further, clinicians should refrain from extrapolating this evidence to using fluid restriction as a therapeutic option for treatment of symptomatic PDA, given there is no evidence to support the latter.

With respect to treatment of asymptomatic or symptomatic PDA, the availability of RCT evidence is substantially variable, depending on the intervention used. Overall, RCT evidence consistently demonstrates that the use of prostaglandin inhibitor drugs is effective in closing a PDA. Despite effective PDA closure, current evidence fails to demonstrate a benefit of prostaglandin inhibitor drugs for patient‐important clinical outcomes, such as need for invasive PDA closure, CLD, or mortality. However, several study limitations prevent us from drawing firm conclusions on the lack of efficacy of the prostaglandin inhibitor drugs for clinical outcomes. First, there was wide variation in PDA definitions in the included trials, especially the trials for treatment of symptomatic PDA. Symptomatic PDA was defined in most trials based on characteristic clinical signs, along with echocardiographic evidence of an increased PDA shunt volume. However, the trials did not have consistent eligibility criteria, from either a clinical or an echocardiographic standpoint. Further, the most used echocardiographic criteria, the PDA size, and the left atrium to aortic root ratio, have been shown to have poor inter‐rater reliability, and therefore, may represent suboptimal inclusion criteria (de Freitas Martins 2018; Zonnenberg 2012). In addition, the trials did not attempt to differentiate between PDAs with moderate versus high shunt volume, based on any clinical or echocardiographic criteria. These drawbacks of existing RCTs may have led us to include a highly heterogeneous population in the meta‐analyses, especially, more mature infants with smaller PDA shunt volumes, in whom spontaneous PDA closure was highly likely to occur. Second, as evident from their wide confidence intervals, the effect estimates for the most important clinical outcomes were imprecise, which failed to provide convincing evidence for an absence of effect on such outcomes. Third, a substantial proportion of infants in the placebo or no treatment group ended up receiving open‐label medical therapy, thereby, likely pulling the effect estimate towards the null. The latter, especially, might be an important reason why effective PDA closure did not necessarily translate into improved longer‐term clinical benefit.

The need for subsequent open‐label therapy, including definitive surgical PDA closure, also highlights the fact that medical therapy, though better than placebo, is by no means a highly effective option for PDA closure. Therefore, most RCTs of medical treatment were essentially trials of drug therapy, rather than the elimination of the PDA shunt. Therefore, despite growing calls for accepting the null hypothesis and abandoning further clinical trials on PDA management, the current evidence underscores the need to clearly establish which PDA shunts, if any, are associated with worse clinical outcomes, and pursue further clinical trials that include only those infants at the highest risk of PDA‐attributable morbidities, and explore highly effective and safe shunt elimination strategies for such clinically important PDA shunts.

### Quality of the evidence

The quality of reviews as assessed by the AMSTAR 2 criteria was variable. We only judged two reviews to be of high quality, while five were of low quality, and two of critically low quality (Table 2). Reviews that we judged as critically low quality failed to use a satisfactory technique for assessing the risk of bias in individual studies, in addition to omissions in other critical domains of the AMSTAR 2 criteria. Of note, none of the included reviews provided a rationale for including only randomised controlled trials in their review. This may be associated with Cochrane Neonatal's approach of traditionally including only RCTs in reviews of interventions to obtain the most unbiased estimates of treatment effects. However, in the absence of well‐done RCTs, other study designs, such as large observational studies, may be an important source of evidence, especially related to the safety of the interventions. Further, the majority of the reviews did not explicitly include information on funding sources for the trials. This did have an impact on the quality of the reviews as per the AMSTAR 2 criteria, as full disclosure of any funding is important to ensure that no financial incentive introduced bias (Lundh 2017).

Only five of the Cochrane Reviews assessed the overall certainty of the evidence using GRADE methodology (Evans 2021; Jasani 2022; Mitra 2020a; Ohlsson 2020a; Ohlsson 2020b). We did not reassess the certainty of evidence, but summarised the certainty assessed by the respective review authors. With regard to the primary outcomes defined in this overview, the certainty of the evidence was not reported for all available interventions. For PDA prevention, the certainty of the evidence, which was available only for prophylactic ibuprofen for the primary outcome of severe IVH, was deemed to be moderate. The certainty of the evidence was not assessed for interventions for the management of asymptomatic PDA. While for symptomatic PDA treatment, the certainty of the evidence for the primary outcome of failure of PDA closure was available for all available prostaglandin inhibitor drugs. The overall certainty for symptomatic PDA closure was high for indomethacin, moderate for ibuprofen, and moderate‐low for acetaminophen. The most common reason for downgrading the certainty of the evidence was serious risk of bias, followed by imprecision in effect estimates.

### Potential biases in the overview process

We are confident that this overview is a comprehensive summary of all currently available Cochrane Reviews on the management of the PDA in preterm infants. We did not apply any date restrictions to the search. Five of the 16 reviews were either first published or updated in the past two years, making this a comprehensive summary of the best available evidence. One potential source of bias is that two of the overview authors are first authors or co‐authors on three of the included reviews. However, quality assessment of the reviews, using the AMSTAR 2 criteria, was carried out in duplicate to minimise any intellectual bias (Table 2).

### Agreements and disagreements with other studies or reviews

With respect to prophylactic therapies, the results of this overview largely align with the recently published Cochrane network meta‐analysis by Mitra 2022. Both the overview and the network meta‐analysis showed that prophylactic indomethacin reduces the risk of severe IVH and the need for surgical PDA closure, increases the risk of oliguria, and likely does not increase the risk of NEC or gastrointestinal perforation. In addition, both the overview and the network meta‐analysis demonstrated that prophylactic ibuprofen also reduces the need for surgical PDA closure, and likely does not increase the risk of NEC or gastrointestinal perforation. However, the network meta‐analysis by Mitra 2022 failed to demonstrate a difference for severe IVH and oliguria with prophylactic ibuprofen, unlike the Ohlsson 2020a review, which showed a marginal reduction in severe IVH, in addition to a definite increase in oliguria. These observed differences in results may be related to corresponding differences in the datasets analysed in the two reviews, as the search for the Ohlsson 2020a review was updated in October 2018, while the search for the Mitra 2022 review was updated in December 2021. However, given the considerable overlap of studies included in the network meta‐analysis by Mitra 2022 and the Ohlsson 2020a review, the more likely rationale for the observed differences in results could be related to differences in analytical methods. While the Ohlsson 2020a review used the traditional Cochrane Neonatal approach of using fixed‐effect meta‐analysis, thereby, generally obtaining more precise estimate of effects, the network meta‐analysis used a Bayesian random‐effects model, which was likely to produce more conservative estimates, especially in the absence of a substantial contribution from the indirect comparisons, thereby, failing to establish differences for the said outcomes. With respect to prophylactic acetaminophen, both reviews failed to draw meaningful conclusions due to overall paucity of evidence. With regard to treatment of symptomatic PDA, the results of this overview align with two previous network meta‐analyses, which both demonstrated that prostaglandin inhibitor drugs were effective in closing a PDA, which however, failed to translate into a clinically meaningful benefit (Jones 2011; Mitra 2018).

Overall, our findings generally support the current recommendations from the Canadian Pediatric Society (CPS (Mitra 2022a)), and the American Academy of Pediatrics (AAP (Hamrick 2020)), that include: considering prophylactic indomethacin to prevent severe IVH in high risk extremely preterm infants, and refraining from pharmacotherapy for PDA closure in clinically stable preterm infants, given the lack of clear evidence for benefit, while judiciously weighing the benefits and harms of PDA treatment in clinically unstable, extremely preterm infants, given the overall lack of RCT evidence in this vulnerable population. However, it is important to note that both the CPS and AAP statements suggest considering invasive PDA closure (surgical ligation or percutaneous transcatheter closure) if the PDA remains persistently symptomatic, despite limited RCT evidence on the benefit of invasive PDA closure on clinically relevant outcomes.

## Authors' conclusions

**Implications for practice**

Prophylactic indomethacin probably reduces severe intraventricular haemorrhage (IVH), while it does not appear to affect the composite outcome of death or moderate/severe neurodevelopmental disability. Prophylactic ibuprofen probably marginally reduces severe IVH (moderate‐certainty evidence), while the evidence is very uncertain on the effect of prophylactic acetaminophen on severe IVH.

All available prostaglandin inhibitor drugs appear to be effective in closing a symptomatic patent ductus arteriosus (PDA) compared to no treatment (high‐certainty evidence for indomethacin; moderate‐certainty evidence for ibuprofen; low‐certainty evidence for early administration of acetaminophen). Oral ibuprofen appears to be more effective in PDA closure than intravenous ibuprofen (moderate‐certainty evidence). High‐dose ibuprofen appears to be more effective in PDA closure than standard‐dose ibuprofen (moderate‐certainty evidence). There is no evidence of a difference in PDA closure effectiveness between the three available prostaglandin inhibitor drugs (low‐ to moderate‐certainty evidence). There is limited evidence on the effect of invasive PDA closure on clinical outcomes.

**Implications for research**

From a PDA prophylaxis perspective, any future clinical trial should only include extremely preterm infants at the highest risk of mortality and major morbidity. Given the low rate of adverse clinical outcomes in older preterm infants, lack of clear benefit, and potential for harm with routine use, there is no clinical equipoise for further clinical trials on prophylactic interventions for PDA in older preterm infants, especially those born after 28 weeks of gestation.

Regarding PDA treatment, future research should focus on defining the infant population, including the PDA characteristics that would benefit most from the elimination of the PDA shunt. Future clinical trials should exclusively enrol this high‐risk population to explore and describe the safest and most effective shunt elimination strategy that leads to meaningful improvement in infant and family important clinical outcomes.

## History

Protocol first published: Issue 4, 2020

## Differences between protocol and review

We made the following changes to the protocol (Mitra 2020b).

1. In this overview, we only included reviews that specifically reported on interventions primarily intended to prevent or treat a PDA, and not all interventions that reported PDA as an outcome. We clarified this under the Types of Interventions section, by adding the following sentence: “In this overview, we specifically included reviews of therapies primarily intended to prevent or treat a PDA”.
